# Triboelectric Nanogenerators for Thermal Management Application: Current Progress and Future Prospects

**DOI:** 10.1007/s40820-026-02110-1

**Published:** 2026-03-05

**Authors:** Jia-Qi Lang, Lei Chen, Xing-Xiang Ji, Qi Liu, Ming-Guo Ma

**Affiliations:** 1 MOE Engineering Research Center of Forestry Biomass Materials and Bioenergy, Research Center of Biomass Clean Utilization, College of Materials Science and Technology, Beijing Forestry University, Beijing, 100083 People’s Republic of China; 2 State Key Laboratory of Green Papermaking and Resource Recycling, Qilu University of Technology (Shandong Academy of Sciences), Jinan, 250353 People’s Republic of China

**Keywords:** Triboelectric nanogeneratorsl, Thermal management, Energy harvesting, Phase change material

## Abstract

The rapid development of wearable electronics, self-powered systems, and the Internet of Things urgently requires efficient thermal management (TM) technologies and sustainable energy solutions. However, triboelectric nanogenerator (TENG) and their integrated electronic components inevitably generate or accumulate Joule thermal. It led to performance degradation or even device failure. This review focuses on the research progress in integrating advanced TM technologies into TENGs, aiming to provide comprehensive insights for constructing high-performance and highly stable self-powered systems. The scope of this review encompasses: (i) systematically summarizing the design and development of TM-TENG systems based on key materials, such as graphene, carbon nanotubes, MXene, cellulose, and phase change materials, (ii) elucidating the bidirectional coupling mechanism between triboelectric charge generation and thermal management, along with a critical analysis of existing theoretical models, and (iii) detailing the multifunctional integration and applications potential of TM-TENGs in the fields including thermal conversion, thermal energy harvesting, storage, actuation, and conduction. The bidirectional coupling mechanisms between triboelectric charge generation and thermal management are thoroughly dissected at a theoretical level. The predominant physical models explaining the interaction phenomena between frictional heating and thermal management are reviewed, with critical analysis of their applicability and limitations. Furthermore, this work discusses the current challenges and future directions in this field and proposes strategic recommendations for realizing more advanced TM-TENG systems. The primary objectives of this review are to synthesize existing knowledge, clarify interaction mechanisms, and promote interdisciplinary development at the intersection of thermal management and energy harvesting.

## Highlights

- A systematic review of recent advances of the process of friction nanogenerator participates in the different thermal management of materials through contact and non-contact thermal sensing.
- Triboelectric materials participated in the application of the whole process of thermal management are reviewed based on the up-to-date works.
- Prospects and challenges for future need for advanced and thermoelectric device designs and integration with existing energy technologies are discussed.

## Graphical abstract

**Figure:**
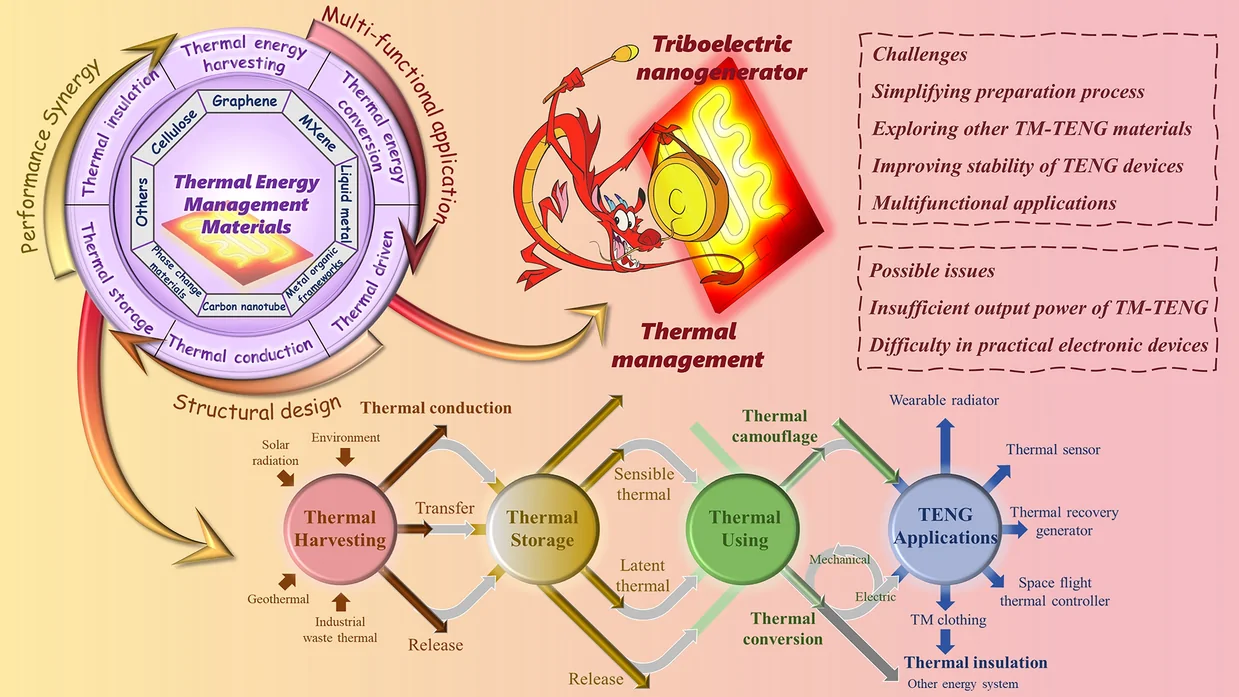

## Introduction

As ecological challenges are increasingly attributed to the unrestrained use of traditional fossil fuels, developing renewable energy has become particularly crucial. Against the backdrop of the global pursuit of “Carbon Neutrality,” it is imperative that we maximize the utilization of all potential renewable energy sources. Conventional energy management technologies often run counter to the overarching trend of green and sustainable development, prompting the emergence of micro-energy sources [1]. Triboelectric nanogenerator (TENG) emerges as a revolutionary technology that efficiently converts disordered, low-frequency, and distributed mechanical energy (including human motion, wind, and water waves) into electricity, based on the coupling of contact electrification and electrostatic induction [2, 3]. Its unique material adaptability enables deployment across diverse scenarios, positioning it as a core enabling technology propelling the Internet of Things (IoT) and sensor networks into a new era [4]. In 2012, Wang et al. [5] had done the pioneering work on TENG, which pioneered a new field of harvesting mechanical energy from the environment, leading to paradigm revolution in the field of micro/nanoenergy and self-powered systems. At its core, it functions by varying a capacitor’s capacitance to create a changing voltage at constant charge, thereby driving electrons through an external circuit. Consequently, the motivation for developing TENG stems from a seamless integration of exploratory energy harvesting and applied technological pursuits [6]. Among these, the dielectric constant directly determines the intrinsic capacitance of this variable capacitor. Researchers can design and control the dielectric constant through advanced methods. The selection of dielectric constants is not a simplistic pursuit of “the higher, the better” or vice versa. It demands a nuanced consideration of the specific application [7]. The objective is to engineer triboelectric materials through precise trade-offs, culminating in an ideal substance that exhibits both high charge generation capacity and favorable electrical matching properties. However, the development of TENG is not to supplant the power grid, but to empower a myriad of microelectronic devices with energy autonomy and application diversity. The prevailing trend lies in a paradigm shift driven by the multifunctional integration of TENGs within the processes of energy acquisition, storage, conversion, and utilization. This integration constitutes the physical foundation for realizing the Internet of everything [8]. However, the output performance of TENGs is significantly influenced by temperature. Elevated temperatures can accelerate the aging and deformation of triboelectric materials, as well as hasten charge dissipation. Conversely, lower temperatures may embrittle these materials, thereby compromising the efficiency of the contact-separation process.

Research and development of renewable energy sources remain immature. Technological constraints also cap the energy efficiency rate at just 43% in developed countries [9]. Throughout energy usage, substantial amount of thermal energy is invariably lost as waste thermal. Therefore, effective thermal management (TM) is of paramount importance for maintaining stable and efficient output from TENGs operating in complex environments. The integration of TENGs with TM capabilities paves the way for a new generation of intelligent systems. It offered a holistic, self-sustaining solution that simultaneously addresses the dual challenges of power supply and thermal comfort for distributed electronics. Hu et al. [10] prepared cellulose nanofiber (CNF)-based composite aerogels with biomimetic layered structure resembling mother-of-pearl. The aerogel exhibits outstanding thermal insulation properties, a wide operating temperature range (− 196 ~ 20 °C). The ability to assemble into TENG for generating triboelectric output could be utilized for super-insulating windows in buildings as efficient TM materials. Jiao et al. [11] developed a multifunctional TENG with the device comprising a triboelectric layer, a silver electrode, a windproof outer textile, and an inner textile lining. The composite possesses both thermal insulation properties and energy harvesting capabilities, paving a new research direction for multifunctional wearable TM device. In summary, the integration of TENG and TM technologies signifies a paradigm shift toward more self-sufficient, intelligent, and comfortable wearable electronics. By enabling the acquisition, storage, conversion, and multifunctional utilization of thermal energy, the synergy is poised to play an increasingly vital role in fields, such as wearable devices and industrial IoT.

**Fig. 1:**
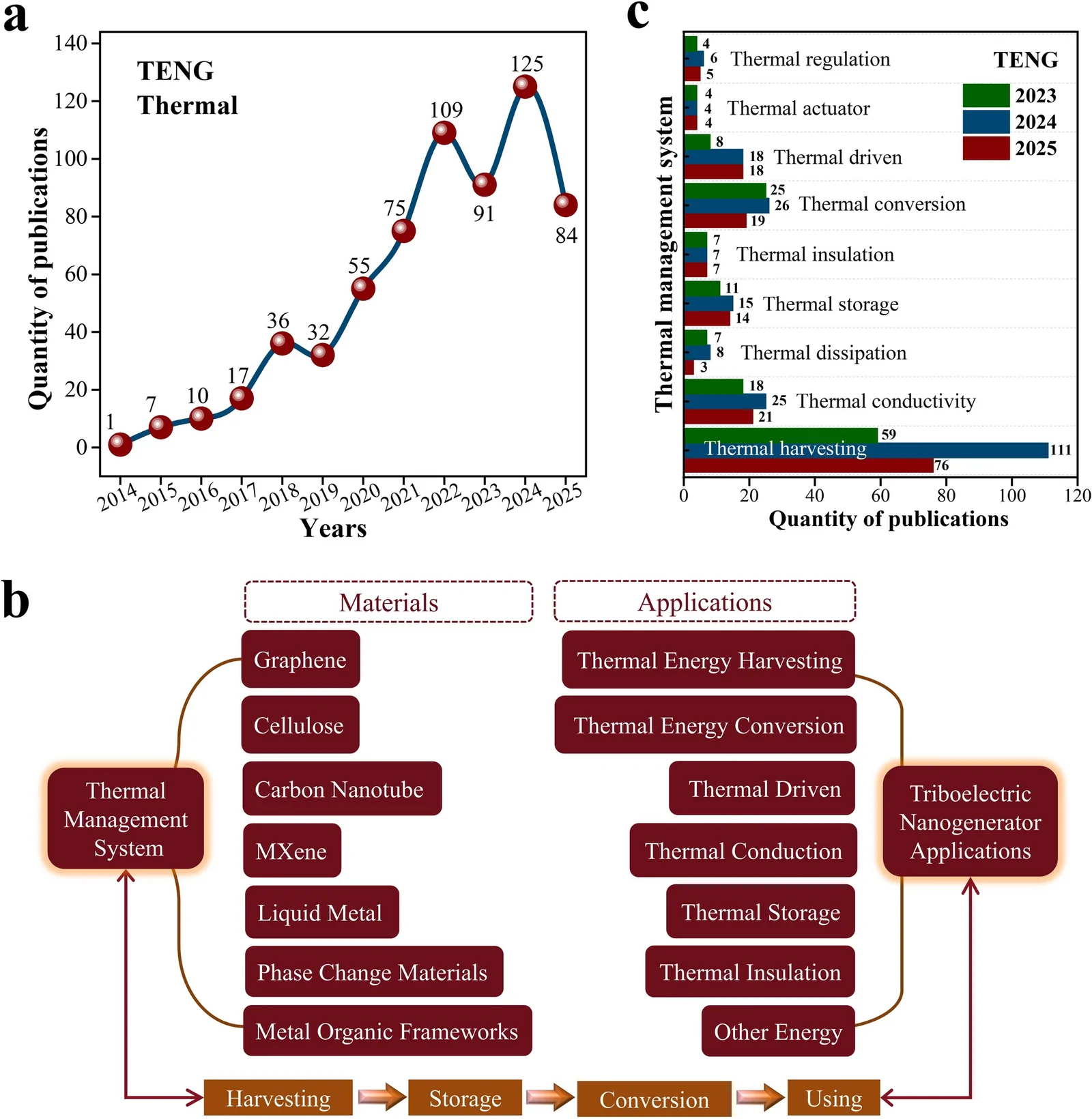
**a** Numbers of publications in the field of thermal energy for TENGs (data collected from the Web of Science, as of September 22, 2025; search term: “triboelectric nanogenerator” and “thermal”). **b** Types of TM systems integrated with TENGs. **c** Number of publications in different TM applications combined with TENG in the past three years

Over the past decade, driven by the global momentum for sustainable energy development and the proliferation of numerous emerging applications, research interest in the fields of TENG and TM has surged significantly. As illustrated in Fig. 1a, the number of publications concerning TENG and thermal energy from 2014 to 2025 demonstrates a fluctuating yet overall upward trajectory. These data unequivocally indicate that research integrating TENG with TM has emerged as a pivotal focus in contemporary academic and technological landscapes. The integration of TENG can be achieved across all facets of thermal energy, including its acquisition, storage, conversion, and utilization. Specifically, thermal energy harvesting and thermal conductivity pertain to the acquisition and transfer of thermal energy. Thermal storage and thermal insulation fall under the domain of thermal energy storage (TES) and regulation. Thermal energy conversion, thermal-driven systems, and thermal actuators represent the transformation of thermal energy (Fig. 1b). Figure 1c presents the total number of publications related to TENG materials for various TM applications over the past three years. A comparative analysis of the publication volume indicates that research on thermal energy harvesting, thermal conversion, and thermal conductivity is relatively the most extensive. The studies on the integration of thermal stimulation actuation with TENGs remain in their nascent stages.

In this review article, we highlight the multifunctional applications of TENG in thermal energy management systems (TEMS). This review begins with a detailed introduction to the role of triboelectric materials in the field of TM, providing a comprehensive overview and classification of the latest research findings and their underlying mechanisms. Subsequently, we summarize and evaluate the preparation strategies, along with their respective advantages and disadvantages, for different representative materials used in both TM and TENG applications. Summary and critical assessment are provided based on the integration of TENG with prominent research directions in thermal energy. Finally, the challenges and future prospect pertaining to this advanced strategy are outlined. The review systematically reviews the fundamental coupling principles, key materials, structural designs, and representative potential applications of TENGs in TM system.

## Introduction of Triboelectric Nanogenerator and Thermal management

### Advantages of Triboelectric Nanogenerator for Thermal Management

**Fig. 2:**
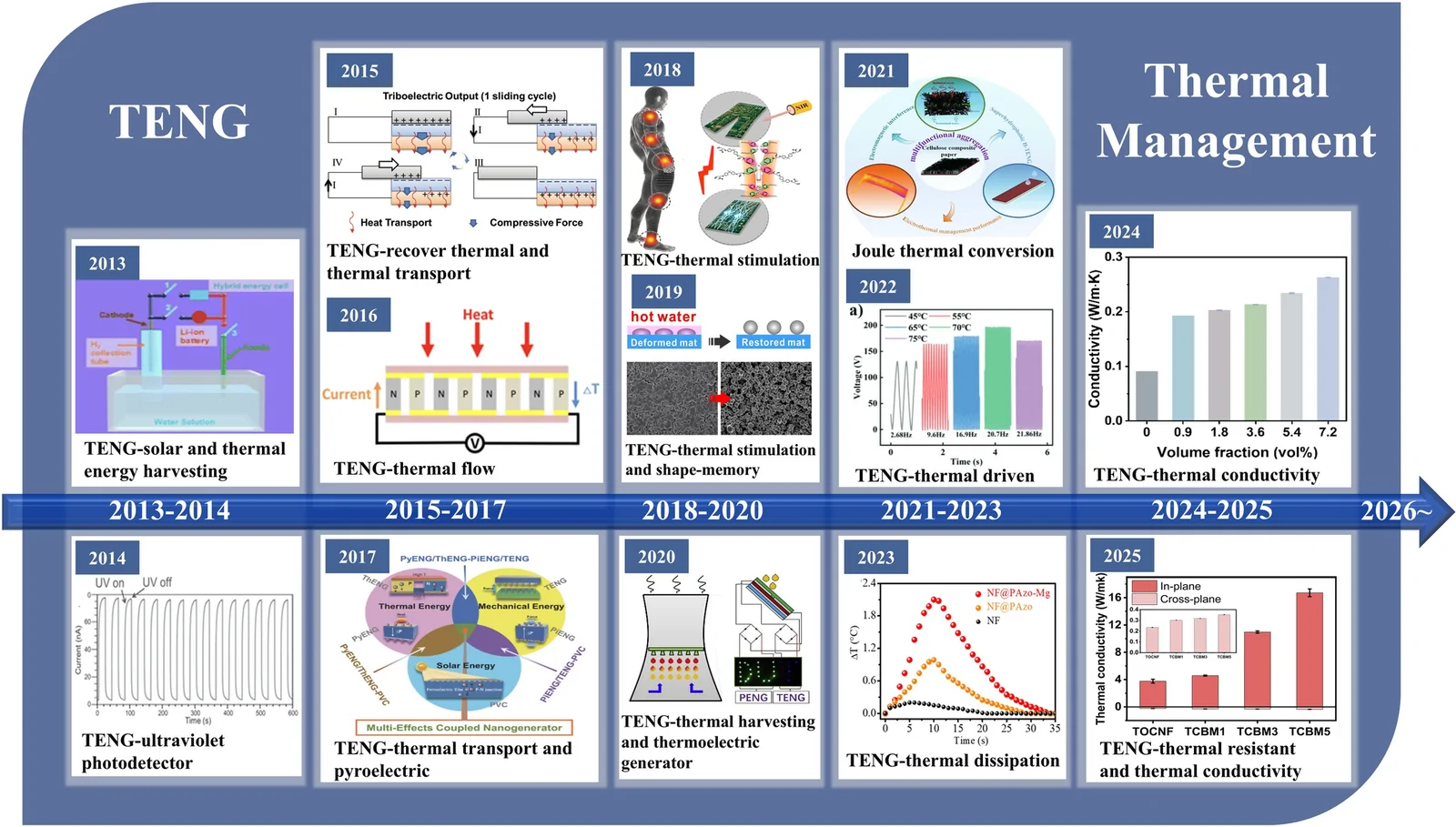
Current trend of application research on TENG combined with TM system in the past decade (from left to right: 1–10). (1) A hybrid energy cell of TENG for harvesting thermal and solar energies [14]. Copyright 2013, The Royal Society of Chemistry. (2) TENG as an active UV photodetector [15]. Copyright 2014, Wiley–VCH. (3) Triboelectric–pyroelectric–piezoelectric hybrid materials for thermal energy harvesting [16]. Copyright 2015, Wiley–VCH. (4) TENG as an active UV photodetector [17]. Copyright 2016, Elsevier Ltd. (5) Hybridized nanogenerator for thermal energies by electromagnetic–triboelectric–thermoelectric effects [18]. Copyright 2017, Elsevier Ltd. (6) Piezo-tribo-pyro-photoelectric effects coupled nanogenerator for simultaneously scavenging mechanical, thermal, and solar energies [19]. Copyright 2018, Wiley–VCH. (7) Near-infrared irradiation induced remote and efficient self-healable TENG [20]. Copyright 2019, Elsevier Ltd. (8) TENG for self-powered thermal temperature sensor [21]. Copyright 2020, Elsevier Ltd. (9) TENG and PENG hybrid energy harvester for kinetic and thermal energy harvesting from thermal fluids [22]. Copyright 2021, Elsevier Ltd. (10) A smart device for photothermal energy utilization [23]. Copyright 2022, Wiley–VCH. (11) Thermal-driven soft-contact TENG for energy harvesting [24]. Copyright 2023, Wiley–VCH. (12) Composite films with thermal conductivity for TENG under high temperature [25]. Copyright 2024, Multidisciplinary digital publishing institute. (13) Composites with thermal conductivity for TENG under high temperature [26]. Copyright 2025, Elsevier Ltd

As electronic devices evolve toward high integration, miniaturization, and increased power density, thermal flux density has risen sharply. Thermal failure has become the primary cause of device performance degradation, reduced reliability, and even damage [12]. From smartphone chips and LED lights to electric vehicle battery packs and data center server clusters, efficient TM is critical to ensuring their safe, stable, and efficient operation [13]. Over the past decade, representative applications integrating TENGs with TEMS have emerged, as illustrated in Fig. 2. The initial impetus for the research stemmed from the thermal environmental challenges faced by TENGs in practical applications. Early efforts primarily focused on utilizing materials with photothermal conversion properties for thermal energy harvesting applications, where TM and TENG applications remained in the disjointed state. Subsequently, research endeavors shifted toward integrating these two domains to achieve synergistic enhancement capabilities. In 2015, a hybrid cell composed of triboelectric–pyroelectric–piezoelectric components was developed. The composites demonstrated remarkable performance in significantly improving energy harvesting efficiency and enabling self-powered sensing and prediction. It integrated a TENG with thermoelectric–piezoelectric nanogenerator. Following this, in 2016, the utilization of thermal flow within triboelectric nanogenerator structures was explored to further enhance performance. A multi-effect coupled nanogenerator based on ferroelectric barium titanate was reported, which improved the capability to simultaneously harness thermal, solar, and mechanical energy. By 2018, researchers began incorporating thermally responsive materials into TENG designs. In 2022, thermal actuation was integrated into the operational systems of TENGs. The following year, researches attention turned toward the thermal dissipation capacity of triboelectric materials. By 2024, multiple studies had concentrated on developing triboelectric materials with high thermal conductivity. Notably, since 2015, the stability of TENGs has remained a significant focus of research. To date, the cyclic durability of most TENG devices has achieved over 10,000 cycles. Current research trends are rapidly advancing beyond the optimization of individual components, evolving toward integrated TM-energy generation-utilization systems. The convergence of TENG and TEMS successfully interconnects the challenges of “waste mechanical energy” and thermal energy, thereby establishing an innovative paradigm that simultaneously addresses the dual demands of power supply and TM. This integration not only constitutes a significant branch within the application spectrum of TENGs, but also represents a pivotal development direction across multiple fields. It includes self-powered intelligent sensing systems, new energy batteries, green electronics, and advanced TM technologies. By transforming the ubiquitous yet often wasted mechanical energy around us into a power source for the digital world that drives myriad devices, its ultimate manifestation will be integrated into our environment seamlessly and imperceptibly. It serves as a vital component in providing sustainable energy for intelligent terminals.

**Fig. 3:**
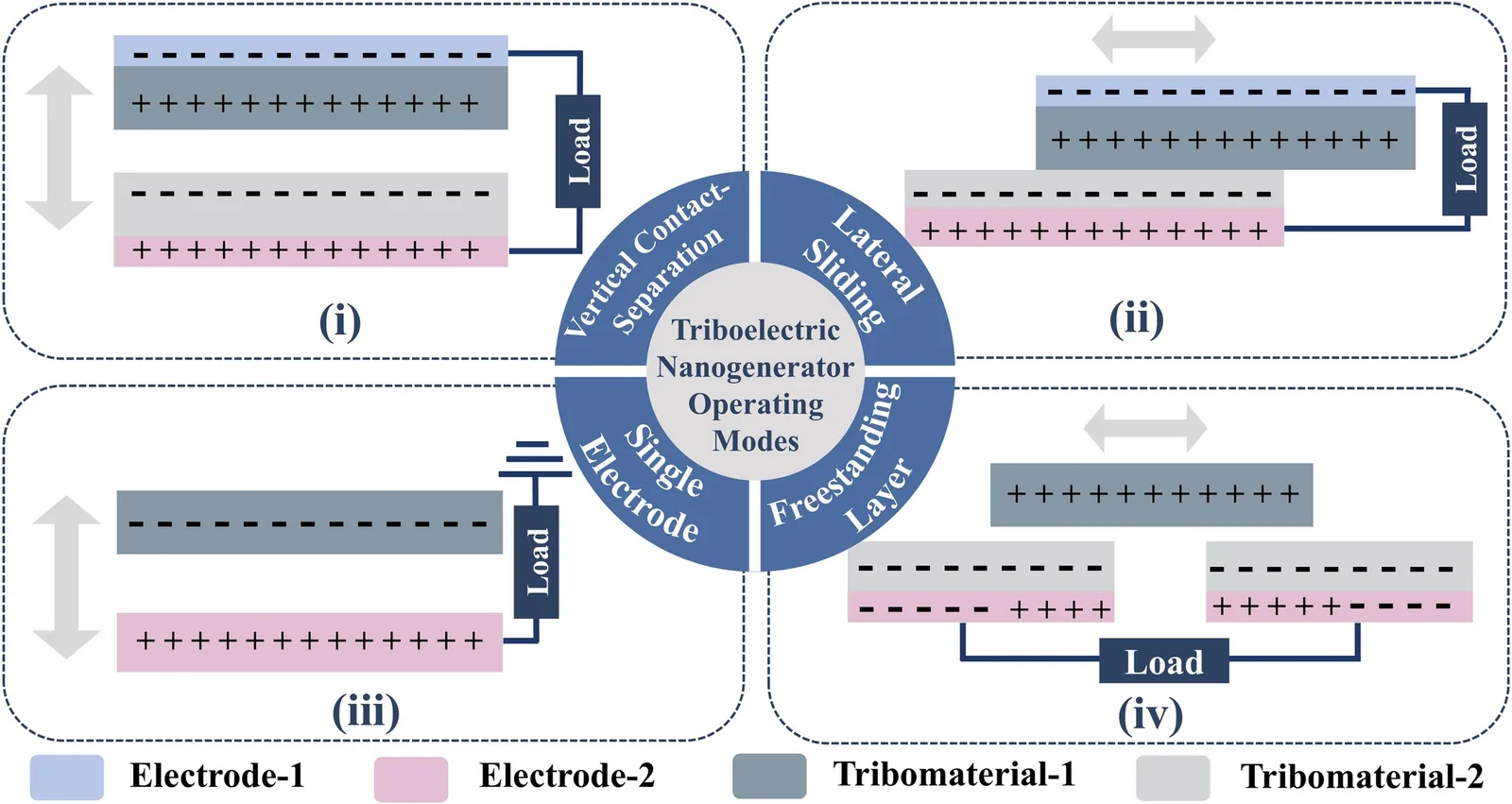
Working mechanisms of TENGs

Based on charge transfer mechanisms and device architectures, TENG systems are categorized into four typical operating modes [27]. These include the contact-separation mode (CS), sliding mode (LS), single-electrode mode (SE), freestanding triboelectric layer mode (FS), and a droplet-based electricity generator proposed in 2020 that can significantly enhance the current and voltage generated by friction [1]. Each configuration has been meticulously optimized to meet energy harvesting demands across diverse scenarios, fully leveraging its unique structural design and charge characteristics. Hybrid modes are sometimes regarded as another significant research direction or extension. Currently, devices utilizing friction-electromagnetic hybrid modes have emerged, capable of simultaneously harvesting multiple energy sources to enhance output efficiency and power [28]. The CS-TENG stands as one of the most frequently employed configurations, representing the most versatile and thoroughly investigated TENG design to date (Fig. 3a). It operates by harvesting energy through the periodic vertical contact and separation of two materials possessing distinct electron affinities. Electron transfer during the contact phase between the positively and negatively charged materials triggers surface charge accumulation. The results in charges being established on the respective surfaces, which in turn drives electrons to flow through an external circuit, thereby generating alternating current [29]. The potential variation and current output of CS-TENG are governed by the distance between its constituent materials [30]. This configuration is particularly well suited for harvesting energy from vertical motions, including vibrations and mechanical impacts. While the CS-TENG remains the preferred choice for harvesting most static or vibrational energy, its stability under extreme environmental conditions or during prolonged operation continues to be a major research focus [31]. To enhance the conversion efficiency and operational durability of sliding-mode TENGs, three-dimensional (3D) multilayer sliding-mode TENG designs, alternating magnetic stripe array TENG architectures. The introduction of lubricants between solid–solid friction layers are being employed [32, 33]. The LS-TENG enables energy transfer during the sliding process, which is driven by the triboelectric properties of the materials (Fig. 3b). This configuration is exceptionally well suited for harvesting mechanical energy from planar motions, including sliding, translation, or rotation, while the LS-TENG achieves excellent triboelectric output charge density and exhibits relatively high energy conversion efficiency. However, its structural design is comparatively complex. Ensuring material stability during repeated sliding cycles presents a significant challenge, rendering it less suitable for long-term, high-speed energy harvesting applications [34]. Leveraging the structural characteristics of LS-TENG, researchers have successfully developed various novel configurations, such as grid-like electrode structures [35], rotating disk structures [36], and rotating cylindrical structures [37]. These innovations have significantly enhanced TENG performance and expanded its applications potential.

However, establishing reliable electrical connections via wires to certain freely moving components within TENG structures presents a significant challenge in practical deployment. The limitation has consequently spurred the development of two operational modes, which is the SE-TENG and the FT-TENG. These modes facilitate electron flow between a single triboelectric electrode and a reference electrode, circumventing the need for direct wiring to moving parts [38, 39]. The SE-TENG is characterized by a configuration where only one active triboelectric electrode is connected to the external circuit, while the other electrode typically serves as bottom conductive layer or is directly grounded (Fig. 3c). The SE-TENG represents the most convenient and integration-friendly operational mode. When mechanical stress induces contact between the triboelectric material and a target object, the disparity in their triboelectric property triggers electron transfer, leading to the accumulation of opposite charges on their respective surfaces [40]. The charge imbalance generated during the subsequent separation process creates a potential difference, thereby driving electrical current. SE-TENG becomes a strategic choice for powering wearable electronics and IoT sensors. However, its triboelectric output performance is comparatively weaker than that of other TENG operational modes [38]. The three before mentioned TENG modes are all significantly susceptible to environmental interference. To mitigate the influence of external factors, the FT-TENG can be employed with superior comprehensive performance (Fig. 3d). Owing to its charge residing on a freestanding layer, this mode exhibits exceptional environmental adaptability, remaining unaffected by factors such as humidity or temperature. A freely moving triboelectric layer and two stationary electrodes constitute an interactive system. The triboelectric layer moves reciprocally between the two electrodes without being permanently adhered to either surface. During the process of contact, charge separation induced by the materials’ triboelectric properties generates a voltage potential between the electrodes, driving electron flow through the external circuit to achieve charge equilibrium. A notable advantage of this configuration is its capability to efficiently harvest energy from irregular, multi-directional mechanical motions. Moreover, tailored to the operational characteristics of FT-TENG, various structural TENG designs have been developed for mechanical energy harvesting, such as rotating disks [41], and rolling spheres [42]. The FT-TENG requires additional mechanical structures to drive the motion of its freestanding layer, resulting in the highest system complexity, yet concurrently offering exceptional durability. The configuration is well suited as an ultimate solution for harvesting ambient mechanical energy on a large scale. It is not ideally applicable for capturing minor or high-frequency vibrations. Instead, its primary suitability lies in harnessing large-scale natural energy sources such as ocean wave energy and wind power. In practical research, hybrid energy harvesters combining multiple modes frequently emerge to balance output performance, durability, and the specific requirements of application scenarios. TENG can also be combined with other power generation methods, such as integrating triboelectric power generation with electromagnetic power generation [43].

Regardless of the operational mode, TENGs exhibit a high degree of customization and tunability in their applications. From a macroscopic perspective, parameters such as contact area, operational frequency, separation distance, and resultant potential difference can be precisely regulated during device assembly, enabling output performance tuning across orders of magnitude [44]. With further research, it has been discovered that specialized processing of the triboelectric layer surface can lead to additional performance optimization. On the microscopic scale, surface microstructures such as microspheres and the arrays can be engineered. The surface modification technique significantly increases the effective contact area beyond the apparent geometric dimensions, thereby markedly enhancing charge generation and output power [45]. Wang et al. [46] prepared a mixture of graphene oxide (GO) and polydimethylsiloxane (PDMS) precursor solution. This mixture was then spin-coated onto a glass substrate measuring 5 cm × 5 cm to form a liquid film with 500 μm thick. Then, a blend of toluene, PDMS precursor, and carbon-encapsulated iron particles was spray-coated onto the surface of the glass substrate. The process yielded a triboelectric layer featuring a fibrous, hair-like microstructure. The unique structural design and fabrication methodology demonstrate significant potential for broad application within the field of TENG.

Besides, guided by theoretical models, the highly precise design and prediction of contact area, operational frequency, and potential output represent one of the most critical aspects of materials engineering in the TENG field. In industrial processes, approximately 20% ~ 50% of energy consumption is dissipated as waste thermal across various gas–liquid interfaces [47]. The thermal energy present at these interfaces holds significant potential for clean energy harvesting [48]. However, conventional TM technologies face significant limitations in practical application, including low efficiency, high cost, and constrained feasibility. It poses considerable challenges to efficient energy harvesting and utilization [49]. The advantage of integrating TENG into TEMS lies not in supplanting traditional heating or cooling capacity, but in introducing a transformative approach through its unique operational mechanism. TENG can effectively capture otherwise wasted low-frequency mechanical and thermal energy from systems or the ambient environment, then converting it into electricity. The generated power may then be directly utilized to drive various TM components [26]. Furthermore, TENGs inherently possess self-powered sensing capabilities, a feature unattainable by conventional TEMS [50]. The output properties of TENGs are directly correlated with the mechanical stimulus, including open-circuit voltage (Voc), short-circuit current (Isc), short-circuit transferred charge (Qsc), and operational frequency. The mechanical parameters are often intrinsically coupled with the thermal generation and dissipation states of the system.

### Coupled Physical Mechanisms of Triboelectricity and Thermal Management

#### Thermomechanical Effects

The electrical output performance of TENG is intrinsically linked to its interfacial friction process [51]. The process itself constitutes strongly coupled thermomechanical phenomenon. Within it, the contact area and material phase transitions induced by frictional heating emerge as two dynamic and interrelated core variables. By altering tribological properties, they significantly influence the thermomechanical behavior and the electrical output of the TENG. In the field of polymer materials, significant advancements have been made in the tribological properties of most composites. Researchers employ a variety of testing methods to comprehensively evaluate the mechanical, thermal, chemical, and aging characteristics of these materials [52]. The friction and wear properties of mechanical components directly impact the service life and reliability of equipment. Investigations into the frictional heating generation mechanisms and friction studies of high-energy materials primarily rely on friction sensitivity testing. Key testing instruments include Kozlov friction pendulum, the large-scale friction pendulum, and the BAM friction sensitivity apparatus [53]. Herein, the influence of friction conditions on friction sensitivity was investigated, revealing that increases in frictional load, coefficient of friction, initial material temperature, and sliding velocity all elevate sensitivity [53]. Pinedo et al. [52] examined the impact of frictional heating on the tribological behavior of thermoplastic polyethylene and nitrile rubber elastomers. Their experimental studies under ambient conditions confirmed the close relationship between frictional heating and friction behavior. However, current research inadequately characterizes the mechanical behavior of materials under external friction, largely overlooking the influence of ambient temperature on friction sensitivity. Furthermore, under identical sliding velocities, an increase in the real contact area augments the total frictional thermal generation. The thermal generated between contacting surfaces may lead to severe deformation, cracking, thermal fatigue, and wear phenomena. Nevertheless, due to surface roughness or porous structures of materials, actual contact may occur only at locally distributed micro-areas during engagement. Consequently, imperfect thermal contact issues must be considered. Oleksii Nosko et al. [54] have addressed the thermal conduction phenomenon in sliding semi-spaces, encompassing the non-stationary thermal conduction problem between two sliding layers with generalized thermal contact conditions. In summary, a larger contact area also provides a more efficient pathway for thermal diffusion from frictional hot spots into the bulk material. It helps reduce the local flash temperature, enhances more sufficient triboelectrification and higher charge transfer quantities, thereby favoring an increase in instantaneous current and power output. However, the concomitant increase in thermal generation may lead to thermal softening of the polymer dielectric layer, alterations in work function, or accelerated charge dissipation. The effects can diminish surface charge density and Voc. Elevated temperatures may also induce material phase transitions, further altering the system’s characteristics.

The accumulation of frictional thermal may induce phase transitions in contact materials (such as crystalline–amorphous transitions or glass transitions), which would exert a significant influence on TENG [26]. When the PCMs are integrated into TENGs as thermal management units, their phase transition process (solid–liquid transformation) absorbs a substantial amount of latent heat. This enables them to function as an intelligent thermal buffer during operation, effectively suppressing rapid temperature increases at the interface and protecting triboelectric materials from thermal failure. Consequently, the phase transition process is regarded as the intelligent TM mechanism. Therefore, designing materials with adaptive tribological properties and controllable phase transition temperatures, allowing TENGs to achieve performance self-optimization within specific temperature ranges, has become increasingly important. Establishing fully coupled models that incorporate frictional thermal generation, contact mechanics, phase transition kinetics, and charge dynamics to predict and optimize these complex interactions is considered highly promising research direction. Some studies have focused on inferring the interrelationships between triboelectric and TM performance through modeling. The finite element method is widely employed to analyze frictional thermal generation in sliding systems. Dittmann et al. [55] integrated isogeometric analysis with mortar contact to enhance thermal analysis and extended phase-field fracture for nonlinear thermoelastic solids. Despite these advancements, analytical predictive models frequently encounter challenges in practical applications due to their inherent assumptions and simplifications. Xia et al. [56] indicated that the analysis using fixed half-plane/rectangle models, such as the Charron model, in friction test machines for thermal analysis is inaccurate. It highlights the inherent complexity in selecting an appropriate thermal model for the microscopic friction interface analysis of TENGs. For flexible, thin-film, or micro-structured TENG devices, the TM design cannot currently rely on the simplistic application of traditional analytical models based on the semi-infinite body assumption. Since most analytical formulas are derived from simplified geometries and linearized problems, accurately estimating contact temperatures during friction remains significant challenge. TENGs offer unique experimental platform to overcome the limitation. Their contact interface can be directly observed under infrared thermography during operation, enabling in situ visualization and measurement of the actual temperature distribution. The intrinsic advantage positions TENGs not only as energy harvesters but also as valuable tools for probing fundamental tribological phenomena.

#### Triboelectric Electron Emission

Temperature variations induce lattice expansion and alterations in electron distribution within triboelectric materials, thereby finely modulating their work function and surface state density. As the surface temperature increases, electrons near the material gain sufficient kinetic energy to potentially overcome the surface potential barrier and enter the vacuum or another material, resulting in thermionic emission [57]. The operation of TENG manifests as the flow of charge between positive and negative electrodes during contact-separation cycles. When the rate of frictional heat generation per unit time exceeds the material’s thermal loss, heat accumulation at the friction interface leads to temperature rise, consequently accelerating chemical reactions. High-temperature interface can enhance electron emission current, which may increase the initial charge transfer quantity. The surface temperature of a triboelectric material is not merely a passive environmental parameter but rather an active key variable regulating the interfacial electron transfer process [58]. The TM encompasses capabilities in thermal conduction, thermal dissipation, and high-temperature resistance. Xi et al. [59] developed power generation device, assembled with rationally designed piezoelectric ceramics, which can rapidly charge commercial electrolytic capacitors at 250 °C with high energy conversion efficiency (*η* = 11.43%). However, within TENGs, multiple mechanisms coexist, including thermionic emission, ion transfer (particularly through adsorbed water layers), and trap-state capture/release. These mechanisms often occur simultaneously, making it difficult to isolate the contribution of any single one in experiments. Attributing changes in TENG output solely to thermionic emission is likely oversimplified, necessitating consideration of the synergistic or antagonistic effects of multiple mechanisms. It opens new avenues for developing self-powered wireless sensors operating in high-temperature environments. Nevertheless, future research on TM-TENGs should transcend the simplistic paradigms of mere thermal dissipation and conduction, advancing toward a new paradigm of precise temperature control to optimize charge dynamics.

## TENGs as Actuators or Sensors for Thermal Management

### TENG in Thermal Management Systems

**Fig. 4:**
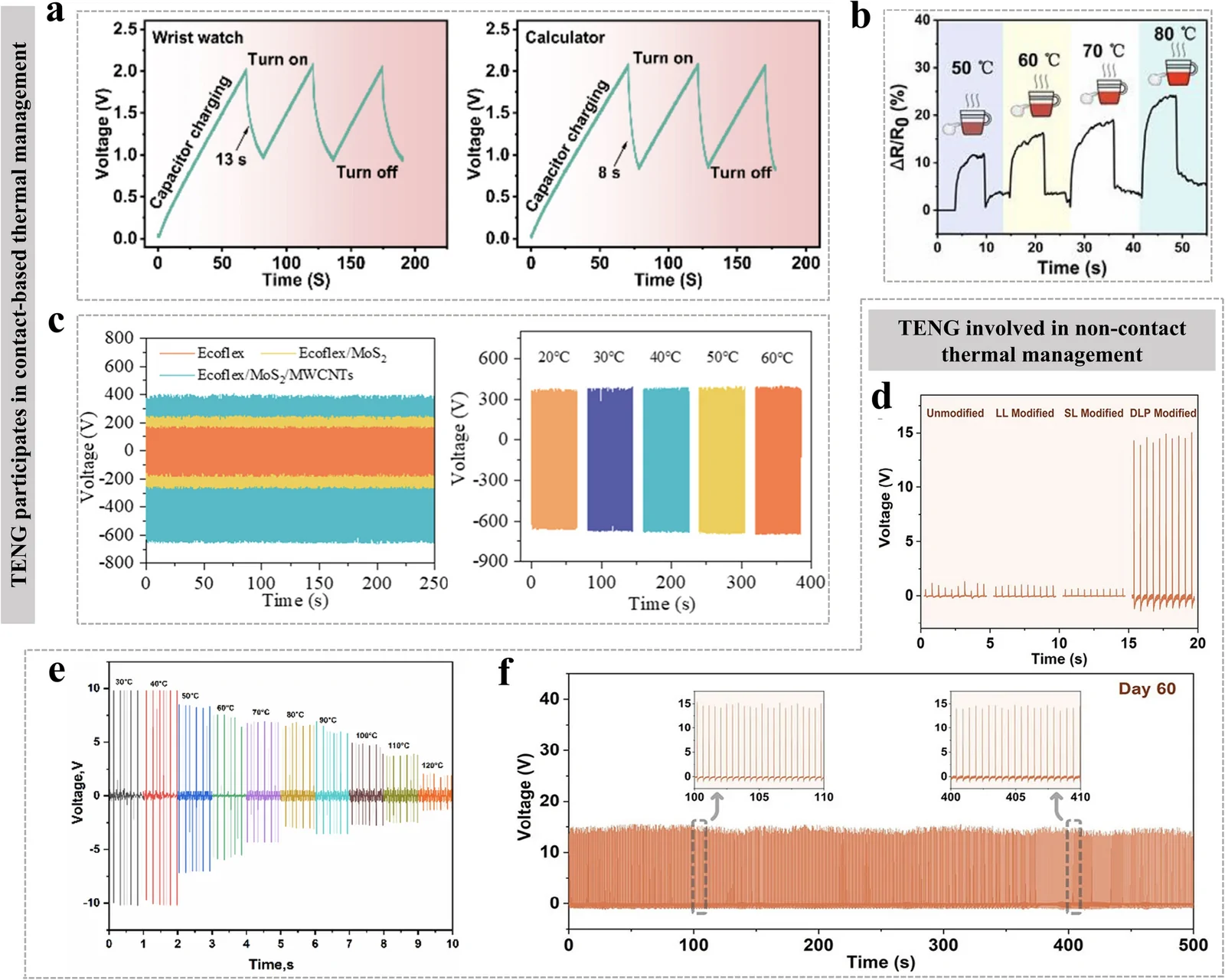
TENG proactively participates in the TM system. **a** TENG for charging and discharging watches and calculators [26]. Copyright 2025, Elsevier Ltd. **b** Electrical response of TENG for different temperatures of thermal stimulation [78]. Copyright 2023, Elsevier Ltd. **c** Effect of material doping on the Voc of the TENG (left). Effect of temperature of thermal stimulation on Voc of the TENG (right) [85]. Copyright 2026, Elsevier Ltd. **d** Voc comparison of TENG based on the four types of triboelectric layers [92]. Copyright 2025, Wiley–VCH. **e** Thermal-dependent in-operando voltage output [73]. Copyright 2022, Elsevier Ltd. **f** Long-term stability test of the TENG, showing Voc after 2 months [92]. Copyright 2025, Wiley–VCH

As a form of energy widely present in nature and daily life, thermal energy continues to be the subject of ongoing research regarding its application methods. Traditional steam engines catalyzed industrial progress and ushered in the first Industrial Revolution [60]. However, a significant amount of waste thermal is dissipated into the environment during the operation of numerous machines. The collection of this low-grade thermal energy, which exhibits a small temperature difference relative to the ambient environment, remains a challenging issue [61]. The characteristic of small temperature difference with the environment precisely provides TENG with unique role. Conventional thermoelectric technologies exhibit limited efficiency under such minimal thermal gradients, whereas TENG demonstrates high sensitivity to subtle thermally induced mechanical motions or temperature variations. It enables TENG not only to harvest waste thermal but also to directly convert the presence and intensity variations of waste thermal into real-time electrical signals. Currently, the concept of TENG’s active involvement in TM systems elevates its role from mere energy harvesting to the intelligent core for energy perception and control. Different methods of utilizing thermal energy according to TENG, thermal energy management can be categorized into passive regulation and active participation in TM-TENG systems [62]. Friction-based materials can utilize environmental and frictional thermal by processing thermal energy through means such as thermal insulation, thermal conduction, thermal storage, and thermal dissipation. This process falls under the passive TM system, wherein much thermal energy is eventually dissipated into the environment. These represent two distinct pathways for TM and thermal dissipation. Passive TM aims to maintain the TENG within stable operating temperature and environment, typically involving materials with ultra‑high or ultra‑low thermal conductivity, PCMs, and related solutions [63]. In contrast, active TM is characterized by thermal energy harvesting, thermal energy conversion, thermal driven, and pyroelectric effects, constituting thermal capture and conversion pathway [64]. The approach can provide additional energy output, and its performance can be evaluated in terms of electrical energy utilization efficiency. The essence of TENG-driven active TM lies in harnessing dispersed mechanical energy from the environment or around the human body through TENG, and directly utilizing generated electricity to power the TM system. Research has already demonstrated the conversion of energy harvested by TENGs into electrical power, eliminating the need for external power sources or batteries [26, 65]. However, most research in this field remains at the stage of passive integration. The concept of active participation represents a more cutting-edge exploration. TENG can actively charge wristwatches and calculators and operate (Fig. 4a). However, there are still relatively few mature cases of deep integration with TM, which presents significant opportunity for the future development.

Theoretically speaking, any TENG device capable of generating electrical energy can be utilized to drive TM system. It embodies the principle of utilizing energy precisely where it is harvested. TENG ingeniously employs insulating materials to generate and confine electrostatic charges, while simultaneously collecting and transmitting energy through conductive electrodes [66]. Previously wasted mechanical energy sources are effectively harvested by TENG, then transforming waste into valuable resources. The alternating current generated is converted to direct current using low-loss rectifier bridges. It is temporarily stored in supercapacitors or micro-batteries [67]. Supercapacitors are particularly suitable for integration with TENG due to their rapid charging–discharging rates and extended cycle life [68]. The electrical energy generated by TENG can directly respond to the state of the human body. When physical activity commences, leading to increased thermal production. The output of TENG simultaneously rises, enabling the system to automatically initiate enhanced TM functionality and achieve intelligent feedback. In practical applications, the inherent characteristics of TENG limit its capacity to directly power most electronic components, such as high voltage, low current, and pulsed output [69]. Key challenges in adopting TENG as the TM device include designing highly integrated, stable, and reliable system architectures, minimizing energy transmission losses, improving the efficiency of energy conversion and utilization, and achieving sufficient output power to drive practical devices. Integrating the TENG, power management circuit, energy storage unit, and TM actuator onto a compact module or flexible substrate offers a promising pathway toward fully self-powered wearable devices and IoT sensors. Each research breakthrough could enhance the output power of TENG devices or improving the efficiency of power management circuits and bring us a step closer to the future realization of this self-powered TM paradigm.

### TENG as Contact Thermal Sensor

The utilization of TENG as a contact-mode thermal sensor epitomizes its intrinsic self-powered sensing capability [70]. Such sensors require no external power supply, performing temperature measurement by detecting variations in their own output signals resulting from temperature changes. Researchers typically fabricate TENG materials for contact thermal sensors through two primary approaches. Firstly, selecting thermally sensitive materials to serve either as TENG electrodes or as triboelectric layers, whose charge generation capacity exhibits enhancement with rising temperature [71]. The preparation of thermosensitive composites currently represents the most effective method for achieving superior performance. Thermosensitive fillers are incorporated into a polymer matrix to form a composite friction layer. TENG thermal sensors require no external power source, deriving energy from perceived mechanical energy while simultaneously performing sensing functions [72]. The same TENG device can simultaneously achieve mechanical energy harvesting, tactile sensing, and temperature sensing [73].

However, the friction polarity and conductivity of the material limit its application in flexible TENG [74]. To address these limitations, contemporary research has focused on incorporating conductive fillers, including metal nanoparticles/nanowires [75], conductive polymers [76], and carbon materials [77]. Furthermore, the conductive properties of composites have been modified by integrating materials with high dielectric constants and polar functional groups, thereby enhancing their ability to dissipate electrons during contact electrochemical processes. Conductive polymers such as polyaniline (Fig. 4b) [78], polypyrrole (PPy) [79], MXene [80], and polydopamine (PDA) [81], which not only enhance the conductivity and triboelectric polarity of composites but also confer exceptional TM performance. Under the assistance of conductive properties in composites, TENG can generate alternating or direct current signals based on the coupling effect between triboelectrification and electrostatic induction. However, it does not imply that all materials within a TENG device must be conductive [82]. When two dissimilar materials undergo contact and separation, charge transfer occurs on their surfaces due to differences in electron affinity, resulting in the acquisition of equal but opposite charges. The “static charges” remain bound to the dielectric surface and do not move freely. Commonly used triboelectric layer materials are all insulators, including polytetrafluoroethylene (PTFE), PDMS, nylon, and silk. Nevertheless, the electrodes must be conductive. The primary function of the electrodes is not to directly generate triboelectric charges, but to facilitate the flow of charges in the external circuit through electrostatic induction. As the static charges on the triboelectric layer surface vary with the device’s motion, opposite charges are induced in the back electrode.

With the rapid advancement of TENG toward multifunctional operational modes and versatile domain-specific designs, it has emerged as a leading power solution for self-powered sensors. The progress has catalyzed transformative research at the frontier of self-powered sensing systems, effectively addressing the dual challenges of external power dependency and frequent battery replacement [83]. However, optimizing the performance of such systems remains a formidable challenge. Current approaches primarily focus on two main methodologies: enhancing the output stability of the energy harvester and optimizing its sensitivity [84]. Ji et al. [85] proposed a high-performance self-powered nitrogen dioxide sensing system. The TENG was assembled using silicone rubber, molybdenum disulfide (MoS_2_), multi-walled carbon nanotubes (MWCNTs), and thermoplastic polyurethane (TPU) lint as triboelectric materials. The TENG demonstrates a peak-to-peak voltage of 1072 V and a power density of 4.07 W m^−2^. Leveraging the impedance matching effect, the developed sensing system exhibits a broad detection range for nitric oxide. The self-powered sensing system operates independently of external energy sources. However, the lint material is susceptible to environmental humidity, which can reduce output stability or compromise triboelectric performance (Fig. 4c). The output capability of this TENG increases as the temperature rises from 20 to 60 °C. For developing highly sensitive gas or chemical sensors, the immense surface area of aerogels offers incomparable advantages [86]. Compared to other forms, films are the most reliable and simplest choice for TENG devices. Fabrics and hydrogels are mostly used to meet the comfort of wearable [87]. Long et al. [88] fabricated an anti-freezing gelatin/polyacrylamide/lithium chloride hydrogel with exceptional electrical conductivity. The composite hydrogel can be effectively applied in strain sensors, pressure sensors, and hydrogel-based TENG. The TENG demonstrates outstanding output characteristics and long-term stability. Many conductive hydrogels exhibit remarkable stretchability and self-healing properties, enabling them to withstand repeated deformation and autonomously repair damage, making them an ideal form for assembling TENGs [89]. Hydrogels represent a highly suitable material platform for sensing applications, yet they are not the optimal choice for TENGs. Containing significant amounts of water, hydrogels undergo continuous evaporation in open environments. It leads to phenomena such as decreased ionic conductivity and altered mechanical properties, ultimately resulting in the failure of TENG functionality. When employed as triboelectric layers, the surface wear resistance of hydrogels is considerably inferior to that of conventional polymer films (e.g., PTFE, fluorinated ethylene propylene, PDMS). During continuous contact-separation cycles, the hydrogel surface is susceptible to wear and scratching, resulting in rapid performance degradation [90]. While research on hydrogels is progressively focusing on the development of multifunctional and high-performance composite hydrogel materials, hydrogel-based sensors for TENG applications are still undergoing steady development. Several representative material configurations in self-powered sensor research each possess distinct advantages and face specific challenges, necessitating the selection of appropriate TENG device architectures according to different application scenarios.

### TENG as Non-contact Thermal Sensor

Scientists have developed non-contact and intermittent contact modes to reduce the contact time between triboelectric materials [91]. Research has demonstrated that preparing flexible contact materials and incorporating liquid lubricants between triboelectric layers can reduce wear, effectively extending the lifespan of equipment [32]. However, reducing the contact time between triboelectric materials leads to degraded TENG output performance. Liquid lubricants suffer from limitations such as poor chemical and physical stability, along with environmental concerns. The output of this TENG is significantly higher than that of other samples (Fig. 4d). Lubricating particles enhance droplet slip rate and separation efficiency while ensuring effective interfacial charge transfer between droplets and the triboelectric layer. Solid lubricants effectively compensate for the shortcomings of liquid lubricants (Fig. 4f). It is the ideal choice for building a long-life TENG [92]. Although research foundations exist for achieving high-sensitivity direct mechanical to electrical signal conversion through contact sensing technology, the evolution of functional requirements has catalyzed the development of non-contact sensing paradigms.

**Fig. 5:**
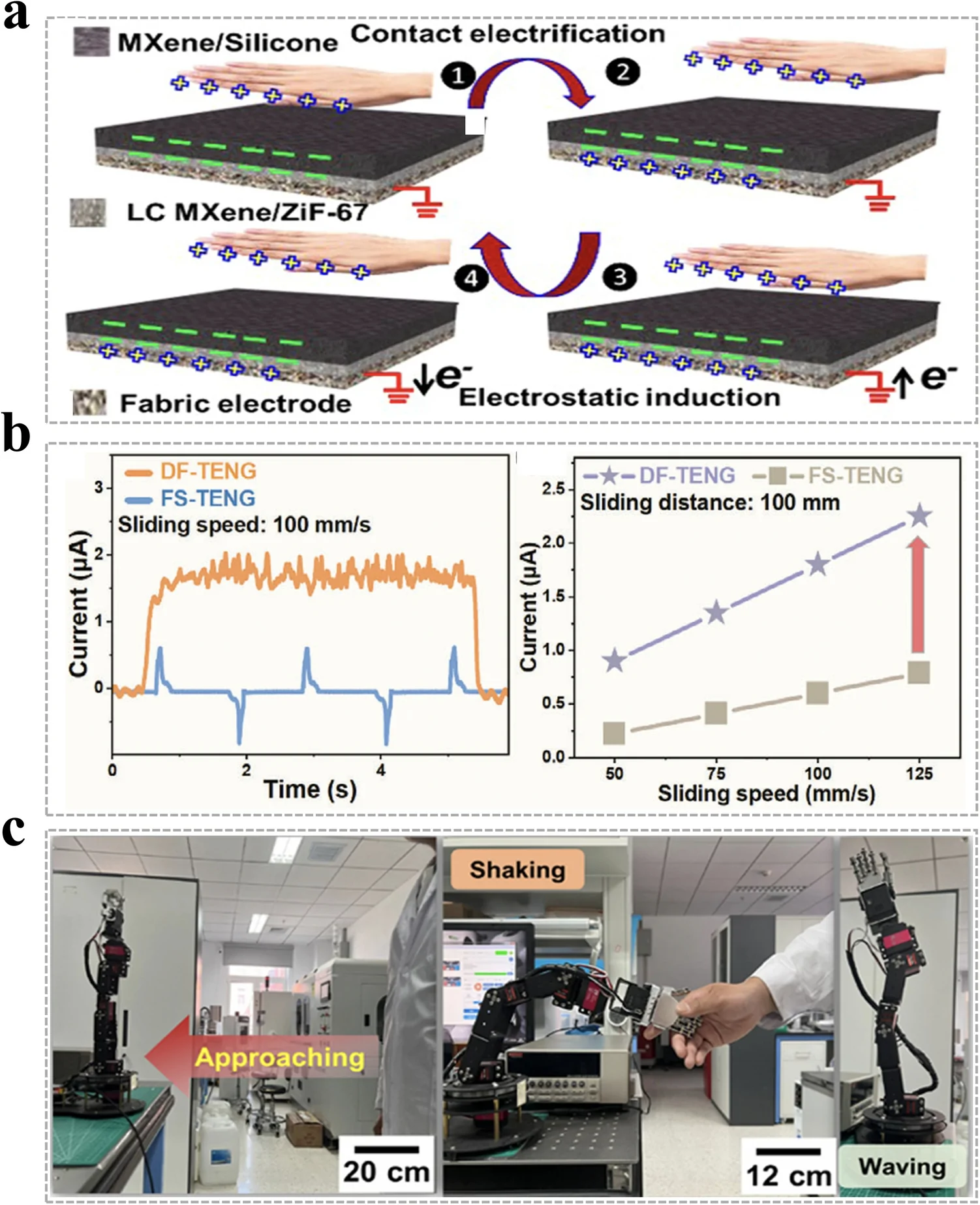
TENG as non-contact thermal sensor. **a** Diagram of the working mechanism of the non-contact nanocomposite TENG [101]. Copyright 2022, Elsevier Ltd. **b** Current characteristics and charge variations of TENG in two modes [102]. Copyright 2024, Wiley–VCH. **c** TENG-based contact/non-contact robotic arm [103]. Copyright 2024, American Association for the Advancement of Science

In 2018, the transformation expanded the sensing dimension from surface physical contact to spatial electric field disturbances, enabling precise perception and functional expansion for intelligent interactive terminals such as vehicle monitoring systems, smart healthcare devices, spatial positioning technologies, and smart screens [93]. By 2024, significant advancements have emerged in remote sensing technologies [94]. The evolution of functional requirements has propelled the development of non-contact sensing modalities. TENG-based non-contact sensing systems have garnered extensive attention for their capability to achieve high-resolution detection of motion, proximity, and position without requiring physical contact [95]. In smart screens, non-contact TENG (NC-TENG) interfaces facilitate gesture recognition and proximity sensing, paving the way for next-generation human–machine interaction interfaces [96]. By leveraging charge capture modulation, extended sensing fields, and integration with machine learning networks, the systems demonstrate capabilities for long-range, multi-target, non-contact environmental perception and event recognition, substantially enhancing their generalizability and intelligence. As an emerging technology in the sensing domain, NC-TENG possess unique advantages [97]. Since their surface charges are not continuously replenished during contactless operation, NC-TENG generates electrical signals without relying on contact friction. This approach avoids device degradation caused by triboelectric materials (dielectric layers) and eliminates instability resulting from sustained friction, thereby significantly enhancing device stability, operational lifespan, and overall system robustness [98]. NC-TENGs enable more convenient, precise, and secure data acquisition and control. However, the power generation efficiency may decline due to charge attenuation. Through optimized structural designs incorporating dielectric constant modulation and charge-trapping interlayers, electrostatic discharge issues can be effectively mitigated, leading to improved charge retention in NC-TENGs. Through functionalization of co-nanoporous carbon [99] and GO-based NC-TENG continuous operation were achieved for 4320 and 600 min, respectively [100]. NC-TENG represents an advanced frictionless sensing strategy, offering enhanced durability and environmental robustness. Yusuf et al. [73] designed self-powered thermal and fire hazard sensors by coupling gravitational potential energy with the triboelectric effect to generate electrical signals. The research leverages the mechanical integrity and phase change characteristics of paraffin wax (PW), which serves as both a mechanical load support and thermal receptor in the sensor (Fig. 4e). The integrated sensor also incorporates multiple communication interfaces and demonstrates an immediate response within the temperature range of 80–89 °C. Some studies have also explored combining contact-type and NC-TENG technologies. This research proposed the potential for materials to enable autonomous, perpetual monitoring without wiring or maintenance. It opened an imaginative technological pathway for designing safety systems in future smart buildings, industrial security, and new energy sectors. Park et al. [101] proposed a contact/NC-TENG by incorporating laser-carbonized MXene/ZIF-67 nanocomposite as an intermediate charge-enhancing layer (Fig. 5a). The porous structure effectively enhances charge density and retention capacity, enabling outstanding output performance in both operational modes. Under optimal conditions, the TENG achieves a power density of 65 W m^−2^ and maintains stable non-contact charge output at distance of 2 cm (~ 15.3 μC m^−2^). This research has successfully laid a robust technological foundation for the future development of ubiquitous self-powered electronic devices, intelligent sensor networks, and human–machine interfaces.

In recent years, NC-TENG has garnered increasing attention due to its unique advantages of high dexterity, comfort, and hygiene [98]. It demonstrated tremendous application potential in fields such as intelligent robotics, virtual or augmented reality, and medical facilities. Tan et al. [102] made a triboelectric non-contact charge injection method utilizing a dual-function TENG (DF-TENG). The DF-TENG generates high voltage and constant current during unidirectional motion, enabling continuous corona discharge for non-contact charge injection into liquid droplets. This method can control the volume of the droplets (3000 µL). In traditional contact-mode TENGs, repeated material friction tends to cause wear of surface microstructures, leading to a decline in charge density over time. Non-contact charge injection fundamentally eliminates mechanical wear, thereby maintaining long-term stability of high charge density. This approach eliminates the need to consider material affinity in friction pairings, allowing for independent optimization of materials for both charge injection electrodes and friction layers. The non-contact charge injection method not only addresses the durability and performance bottlenecks of traditional TENGs but also brings innovation to the design paradigm of TENGs by balancing the charge source and the mechanical system. The momentum of the TENG reaches 115.2 g mm s^−1^, which is five times the highest value recorded by conventional methods (Fig. 5b). Moreover, Du et al. [103] proposed for the first time the concept of remote perception and have designed a highly sensitive electronic skin with multimodal remote sensing capabilities (Fig. 5c). By implementing structurally arrayed doping of inorganic nanoparticles, the precision of remote perception in this electronic skin has been significantly enhanced. The sensor surpasses the performance of conventional NC-sensors, achieving a sensitivity of 14.2 (ΔV/Δ*d*). The research substantially improves the accuracy of remote interaction and robotic manipulation, while opening new avenues for the development of multimodal perceptual systems. Overall, the current integration of TENG with thermal technology primarily focuses on two directions, which is thermal energy integration and thermal energy harvesting for power generation [48]. Thermal energy integration represents a relatively mature approach, with its core emphasis on utilizing photothermal effects to provide active heating and other TM functions for wearable devices. It ensures optimal operational temperature while simultaneously achieving energy harvesting and sensing capabilities [104]. In contrast, the strategy of synergistic thermal energy harvesting for power generation concentrates on directly converting ambient thermal energy into electricity. The triboelectric–thermoelectric synergistic nanogenerator employs thermoelectric modules to capture temperature gradients, complementing the mechanical energy harvested by TENG [105]. It jointly powers monitoring equipment, making them ideal for industrial settings where both vibration and temperature variations are present. However, both contact and non-contact thermal sensors inherently require certain power consumption for operation [106]. When integrated into TENG systems, it leads to relatively low net output power. Furthermore, research on combining thermal sensors with TENG falls within an interdisciplinary domain, demanding deep integration of materials science, electronics, thermal physics, and other fields. This area remains in the early stages of exploration.

## Materials for TM-TENGs

During high-speed and high-frequency tribological processes, TENGs generate significant interfacial thermal accumulation [107]. It not only accelerates material aging and performance degradation but also causes discomfort in wearable applications. In recent years, TM materials have evolved beyond traditional single-function “thermal insulation” or “thermal conduction” toward dynamic, intelligent, and adaptive capabilities [108]. As evident from the operational principle of TENGs described above, the quantity of charges generated through contact electrification between substrate surface materials is intrinsically related to the triboelectric pair selection, which directly influences the final output performance and characteristics of TENGs [109]. Currently, the negative electrode materials for TENGs are transitioning from conventional polymers to various fresh composites. The core objective is to enhance output performance, environmental stability, and additional functionalities. The material selection for TENGs encompasses a wide range, including natural materials such as glass, wood, fur, and paper, as well as polymeric materials like nylon, PTFE, and polyvinyl chloride [110]. The electron gain/loss capability of triboelectric materials directly determines key performance parameters such as Voc, Isc, Qsc, and surface charge density. When designing TENGs, selecting the optimal triboelectric material pair constitutes the crucial first step [111]. Considerable research has been conducted to identify and understand triboelectric characteristics and their relative rankings. Researchers typically select two dielectric materials with significant electron affinity differences from the triboelectric series to achieve higher charge transfer efficiency and surface charge density [112]. Nevertheless, comprehensively mapping and quantifying the triboelectric characteristics of all possible material combinations remains a significant challenge. Early researchers utilized fundamental triboelectric series to qualitatively rank materials based on their relative donor or acceptor tendencies, thereby facilitating initial pairing selection. Scientists are also actively pursuing the discovery of entirely revolutionary materials with exceptional triboelectric properties. Materials developed for TM and TENGs address critical bottlenecks in their respective fields while enabling innovative self-powered and environmentally adaptive TEMS. To facilitate the translation of the performance of these innovative materials into practical applications and assist researchers in selecting appropriate materials for specific scenarios, Table 1 provides a systematic comparison and summary of the existing major triboelectric material systems based on key parameters such as output power density, operational temperature range, and technical challenges. These advancements are poised to exert profound and far-reaching impacts on the IoT, wearable devices, green building technologies, and future energy systems. Herein, we focus on reviewing the current status of several popular and representative applications of TM materials for TENG.

**Table 1:** Summary of the major triboelectric material systems based on key parameters

Materials		Voc (V)	Power density	Temperature range (°C)	Cyclic	Refs.
Graphene	PVDF/GN foam	270	2.78 W m^−2^	RT	–	[113]
	10 wt% RGO/PVDF	23,000	4.09 mW cm^−2^	~ 25	–	[114]
	RGO/polyimide	58	3.5 mW cm^−2^	RT	8,013	[115]
	Non-woven fabric/polyimide/flash GO	300	17 mW cm^−2^	RT	–	[116]
	LIG/PDMS	190	20.2 μW cm^−2^	RT	3,000	[117]
Cellulose	Bacterial cellulose composite	128	4.44 W cm^−2^	RT	5,000	[118]
	CNF/MXene/PEG	155	–	RT	15,000	[119]
	Carboxylate CNF/GN/citric acid	177	68.45 mW cm^−2^	RT	15,300	[120]
	Cellulose/CNT/polyvinylidene	130	0.61 W cm^−2^	RT	100,000	[121]
	PVA/AlCl_3_/ZnCl_2_/CNF-C/borax	4	–	~ 60	1,500	[122]
CNT	Fluorocarbon plasma-etched PDMS–CNT	78	3.29 W cm^−2^	RT	100,000	[123]
	Hydroxypropyl cellulose/chitosan/CNT	18	–	RT	10,000	[124]
	MWCNTs/PVDF-TrFE	34	0.01 W cm^−2^	RT	15,000	[125]
	PDA/MWCNT/PANI/DTMS	200	2.5 W cm^−2^	45.1 ~ 65.5	3,000	[126]
MXene	CNF/BNNS/MXene	80	0.27 W cm^−2^	~ 270	8,000	[26]
	Poly(VS-co-HEA)/phytic acid/BNNS/MXene	88	0.14 W cm^−2^	~ 363	4,000	[127]
	β-Ni(OH)_2_/MXene	428	482.9 mW cm^−2^	~ 26	7,000	[128]
	MXene-treated cotton	40	2.02 mW cm^−2^	RT	100,000	[129]
LM	LM inner-encapsulated all-nanofiber TENG	179	176 mW cm^−2^	RT	10,000	[130]
	Liquid metal	53	–	RT	10,000	[131]
	Cd-In-Sn–Pb-Bi eutectic alloy	940	45.1 mW cm^−2^	~ 25	10,000	[132]
	Thermoacoustic heat engine and LM-TENG	19	0.1 W cm^−2^	RT	–	[133]
PCMs	Photo-responsive liquid paraffin/Fe_3_O_4_	24	50 W cm^−2^	RT	–	[134]
	CNT/PEG/α-cyclodextrin/poly(ethylene oxide)	136	385 mW cm^−2^	RT	–	[135]
	PEG/PDMS/PA-PE	306	236.67 W cm^−2^	RT	–	[136]
MOFs	ZIF-67/Zylon	71	0.11 W cm^−2^	RT	10,000	[137]
	UiO-66 (Zr)-F4 MOF	22	0.78 W cm^−2^	-21 ~ 25	5,000	[89]
	PILs/MOF-LDH-Zr	460	795 mW cm^−2^	~ 34	13,000	[138]

*Voc* open-circuit voltage, *RGO* reduced graphene oxide, *RT* room temperature, *PVDF* polyvinylidene fluoride, *GN* graphene, *LIG* laser-induced graphene, *PDMS* polydimethylsiloxane, *CNF* cellulose nanofiber, *PEG* polyethylene glycol, *MWCNTs* multi-walled carbon nanotubes, *PVA* polyvinyl alcohol, *PDA* ]olydopamine, *PANI* polyaniline, *DTMS* dodecyltrimethoxysilane, *BNNS* boron nitride nanosheet, *LM* liquid metal, *PCMs* phase change materials, *MOFs* metal–organic frameworks, *PA* polyamide, *PE* polyethylene, *LDH* layered double hydroxide

### Graphene

**Fig. 6:**
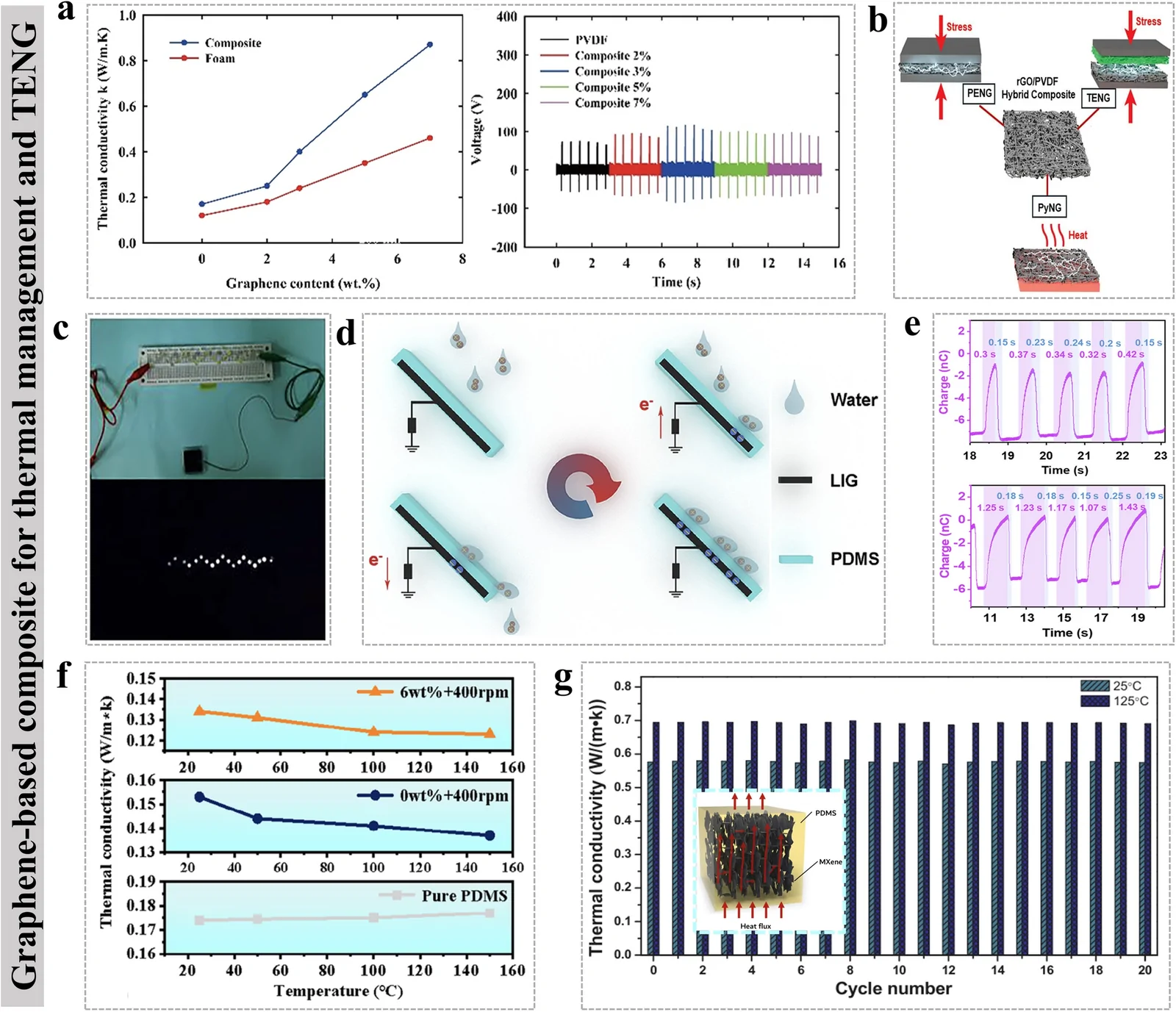
TM-TENG application of GO-based composites. **a** Variations of the thermal conductivity of the in situ 3D-printed PVDF nanocomposite foams (left). The output voltage curves of the nanocomposites (right) [113]. Copyright 2025, Elsevier Ltd. **b** Thermoelectric nanogenerator based on the composite fibers for energy harvesting and multifunctional applications [114]. Copyright 2022, Elsevier Ltd. **c** GO-CC-TENG lighting LED [152]. **d** Working mechanism of the M-TENG at watering mode [152]. Copyright 2020, Elsevier Ltd. **e** Real-time output of a CGC-TENG with (up) rapid gesture triggering and (down) slow gesture triggering [120]. Copyright 2025, Elsevier Ltd. **f** Thermal conductivity of the composites under different preparation methods and temperatures [155]. Copyright 2025, Wiley–VCH. **g** Thermal conductivity of nanocomposites upon multiple heating and cooling cycles (Illustration: Schematic illustration of the enhanced through-plane thermal transfer of nanocomposites) [156]. Copyright 2020, Elsevier Ltd

Wearable technology is driving current research into self-powered TENG devices, with new high-performance materials like GO and low-cost manufacturing processes gaining significant attention [139]. GO is a two-dimensional (2D) material formed by a single layer of carbon atoms tightly packed in a hexagonal honeycomb structure, and it is also the world’s hardest nanomaterial [140]. The studies of Wang et al. [141] and Liu et al. [142] in 2010 demonstrated the ability of GO to store charge, increasing the applicability of GO in TENG. GO typically exhibits excellent electron mobility, high transparency, and outstanding electrical and thermal conductivity. GO is one of the materials with the highest known thermal conductivity [143]. Highly ordered GO films exhibit excellent thermal conductivity (~ 1800 W m^−1^ K^−1^). However, due to the weak interface interactions between GO layers, structural reliability and processability remain challenging [144]. Incorporating GO as a filler or coating in the friction layer or electrodes of TENGs can significantly enhance the overall thermal conductivity of the device. It prevents localized overheating caused by friction-induced thermal or Joule heating, ensuring long-term, efficient, and stable operation of TENGs (Fig. 6a) [113]. Furthermore, the preparation of TENG materials enables modulation of GO’s electrical conductivity through strategies such as impurity/dopant introduction, multilayer stacking, application of physical strain, and environmental condition control [145]. The oxygen functional groups decorated on both the basal planes and edges of reduced GO facilitate electron acquisition, making it suitable for application as a triboelectric material in TENGs [146]. Due to its carbon network substrate and edges being decorated with oxygen functional groups, it is rich in negative charge. Guo et al. [147] reported a SE-TENG based on GO. The SE-TENG not only exhibits higher energy harvesting efficiency but also demonstrates excellent mechanical durability and lightweight properties. Sukumaran et al. [114] developed a multifunctional reduced GO (rGO)/polyvinylidene fluoride (PVDF) nanofiber generator capable of converting mechanical and thermal energy into electricity via piezoelectric, triboelectric, and pyroelectric effects (Fig. 6b). The 7 wt% rGO/PVDF composite exhibits a remarkable triboelectric power density of 3.37 ± 0.72 mW cm^−2^ and multifunctionality under thermal fluctuations. The synergistic triboelectric and pyroelectric currents reach up to 33 nA. This study represents the first systematic integration and explicit differentiation of three distinct energy conversion mechanisms (piezoelectric, triboelectric, and pyroelectric), which overcame the previous research limitation of multiple effects being conflated.

Moreover, GO imparts enhanced output performance and mechanical durability to TENGs due to its excellent flexibility, high elastic modulus, large specific surface area, and superior capacitance [148]. With its nanoscale dimensions, exceptionally high specific surface area, and outstanding mechanical properties, rGO has emerged as an ideal reinforcing phase for polymer nanocomposites. Cai et al. [149] synthesized flexible cellulose/collagen/oxidized GO films for TENG applications. At an external mechanical energy motion frequency of 1 Hz, the composite film exhibited optimal output performance with Isc, Voc, and Qsc values of 0.75 μA, 91.4 V, and 28.7 nC, respectively. The TENG can be used to illuminate LED lights (Fig. 6c). When the load resistance is 400 MΩ, the maximum power and power density of the GO-CC-TENG are 196 μW and 31.36 W m^−2^. However, the maximum output power density of the vertical LS-TENG with rGO is 30 times higher than that of the device without rGO sheets [150]. GO and rGO both play a significant role in the fabrication of precision electrodes for TENG devices. They could enhance the power density and charge storage capacity of the electrodes, thereby improving the output performance of TENGs.

Low-cost and rapid fabrication methods for TENG flexible electrodes have become a current research hot spot. Laser-induced GO (LIG) technology enables transient ablation of carbon-containing precursors, thereby reducing production costs [151]. The LIG technique enables the one-step, mask-free conversion of low-cost carbon precursors into porous graphene through direct laser writing. The process requires no catalysts, vacuum conditions, or complex chemical procedures, significantly reducing both equipment investment and raw material costs. As solvent-free and chemical-waste-free “dry” processing method, LIG aligns with the principles of green manufacturing. This approach simultaneously addresses three critical challenges: reproducibility, cost-effectiveness, and environmental sustainability. Based on this ablation method, it can be observed that the GO-TENG obtained through the approach exhibits excellent thermal resistance properties. The research lays a solid foundation and provides a reference framework for the future development of GO-TENG as a TM material. Li et al. [152] developed an integrated self-cleaning and self-charging device based on LIG. The system has capable of harvesting energy from swaying crop leaves and raindrops (Fig. 6d). The lotus-leaf-inspired microstructure enhances the self-cleaning capability of the TENG device while maintaining excellent electrical conductivity in humid environments. The continuous refinement of these properties establishes GO-electrode-based TENGs as a versatile and highly efficient energy harvesting solution. Wei et al. [120] fabricated a conductive film with exceptional acid and alkali resistance using carboxylic CNF/GO nanoplatelets (GN)/citric acid. Assembled into a SE-TENG, the composite functions as both the electrode and the triboelectric layer. The TENG demonstrates capabilities as a mechanical stimulus sensor with rapid response of 0.35 s and short recovery time of 0.25 (Fig. 6e). Owing to its extraordinary intrinsic thermal conductivity and unique 2D structure, GN exhibits disruptive application potential in the field of TM. It positioned as a critical material for constructing next-generation high-efficiency TM systems.

While GO electrodes have demonstrated satisfactory performance in TENGs during small-scale experiments, ensuring the technology’s consistency and stability across diverse environmental conditions remains inadequate for commercial applications. The commercialization of this technology requires careful consideration of market demand and acceptance, necessitating comprehensive market research. Advances in GO processing techniques are propelling TENGs toward self-powered wearable sensing systems with high energy harvesting efficiency, extended lifespan, and enhanced stability [153]. The charge transfer mechanism and contact area between GO-based TENG electrodes and triboelectric layers are critical factors for enhancing energy harvesting efficiency. The development prospects of GO-based TENGs in TM position them as an ideal platform for constructing a new generation of self-powered, intelligent, and integrated TM systems [154]. Such materials should maximize and balance GO’s outstanding electrical, thermal, and mechanical properties to achieve closed-loop intelligent control from energy harvesting to TM. By purposefully functionalizing the electrode surfaces of the device, system robustness can be enhanced, thereby extending the service life and maintaining high stability of GO-based TENG systems. Wang et al. [155] synthesized a core–shell structured composite through high-temperature carbonization, which was subsequently incorporated into PDMS to form a robust composite foam (EPGP) with exceptional thermal insulation and flame-retardant properties. The EPGP-TENG demonstrates Voc of 186 V and Isc of 0.3 μA cm^−2^ at room temperature. Even when subjected to 200 °C, it maintains remarkable thermal resistance while sustaining stable outputs of 106 V and 0.16 μA cm^−2^. The TENG achieves reliable operation under high-temperature conditions, thereby advancing the development of thermal-insulating composites for TENG-based microelectronic devices in extreme environments (Fig. 6f). Wang et al. [156] proposed a straightforward method for constructing 3D MXene architectures without using adhesives. After incorporation into PDMS matrix, the resulting 3D MXene/PDMS nanocomposite exhibits an exceptional electrical conductivity of 5.5 S cm^−1^, nearly 14 orders of magnitude higher than that of pure PDMS. The nanocomposite demonstrates an approximately 220% enhancement in thermal conductivity even at low MXene loading (Fig. 6g). This study employs an original 3D structural construction method to achieve a synergistic leap in electrical, thermal, mechanical, and energy harvesting performance simultaneously at extremely low filler content.

### Cellulose and Its Derivatives

Against the backdrop of sustainable development, cellulose and its derivative materials are gaining prominence due to their eco-friendliness, biodegradability, and surface modifiability [157]. Cellulose-based TENGs offer advantages such as wearability, flexibility, environmental compatibility, and scalability, demonstrating considerable application potential in wearable energy and self-powered sensing domains [158]. Benefiting from the dense hydrogen bond network and tunable molecular structure of cellulose, cellulose-based triboelectric materials exhibit outstanding mechanical properties and charge storage capacity [159]. However, current cellulose-based TENGs face challenges including low output power density, complex structural designs, poor environmental adaptability, and limited durability. High-porosity materials, represented by cellulose, can significantly increase the effective contact area of TENG, promoting interfacial charge generation and transfer. It contributes to enhancing the output strength of the triboelectric effect. The microscale concave–convex interfaces formed by the porous structure help establish more active charge sites, thereby increasing the charge accumulation density per unit area, improving energy conversion efficiency, and optimizing the intensity and stability of the triboelectric effect. The pore size of triboelectric materials determines the charge distribution pattern during friction, which in turn influences the efficiency of charge transfer and the stability of charge accumulation. Larger pore sizes provide more space for the friction process, accommodating greater charge accumulation areas and thus enhancing charge buildup. However, excessively large pores may lead to charge loss and reduce accumulation stability. Smaller pore sizes, while possibly resulting in weaker triboelectric effect, feature denser pore structure that facilitates the formation of finer micro-contact points. It allows for more precise charge accumulation during friction, strengthens localized surface charge accumulation capacity, and thereby improves the stability and durability of the triboelectric effect. In the design of triboelectric materials, some studies have fabricated composites into Janus structures [160]. The approach involves introducing high thermal conductivity layer as an independent thermal diffusion pathway or utilizing infrared radiation layer to enhance surface radiative cooling. These strategies effectively reduce the operating temperature at the friction interface, thereby preventing thermal failure. Moreover, physical isolation helps avoid the negative impact of high thermal conductivity fillers on the surface triboelectric properties. Many studies have focused on the synergistic enhancement strategy of multiphase interface mixing to prepare composites [26]. Current research partly involves structural design from macroscopic perspective, including gradient structures, Janus structures, and others. However, TENGs based on macroscopic structural design often do not demonstrate superior cycling durability. Therefore, most studies focus on constructing complex structures from a microscopic perspective, such as at the micron or nanometer scale. These include biomimetic composite structures and porous skeleton composites. Within these microstructures, numerous interfaces form between the fillers and the matrix, which can act as deep traps to capture and stabilize triboelectric charges, thereby increasing surface charge density and output stability [161]. Additionally, the introduction of fillers may alter the dielectric constant and polarization capability of the composite, consequently influencing the triboelectric output.

**Fig. 7:**
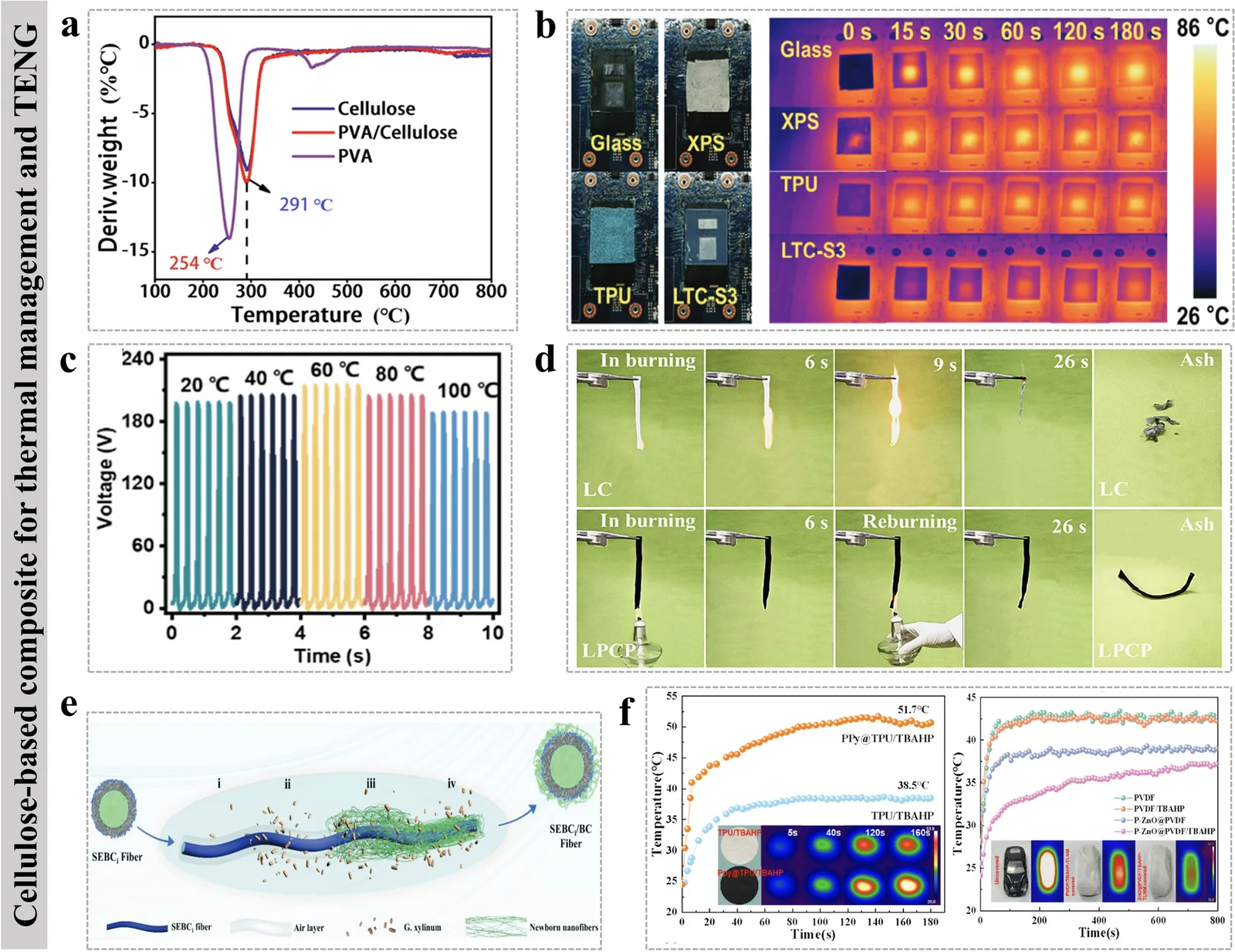
TM-TENG applications of cellulose-based composites. **a** DTG curves of folding PVA/cellulose composites [165]. Copyright 2026, Elsevier Ltd. **b** Photographs, infrared images variations of LTC-S3, glass, XPS, and TPU placed on the working CPU for 3 min [10]. Copyright 2024, Wiley–VCH. **c** Influence of thermal on voltage of the composites [169]. Copyright 2024, Elsevier Ltd. **d** Optical images of vertical combustion tests of LPCP-TENG and original LC fabric [170]. Copyright 2024, Elsevier Ltd. **e** Schematic diagram of bio-fabrication process [173]. Copyright 2023, Wiley–VCH. **f** Surface temperature variation curves and corresponding infrared thermogram under light irradiation of 1 kW m^−2^ (left). Surface temperature variation curves and corresponding infrared thermogram under light irradiation of 1 kW m^−2^ (right) [174]. Copyright 2025, Elsevier Ltd

Moreover, in high-temperature environments, cellulose-based TENGs may experience performance degradation due to thermionic emission effects. To enhance material performance, researchers are employing three optimization strategies, such as physical modification, chemical modification, and the preparation of composite conductive materials [26]. With large specific surface area and excellent mechanical properties, nanocellulose directly enhances charge generation and retention efficiency. When employed as a triboelectric electrode, nanocellulose demonstrates the potential to overcome limitations inherent in metal electrodes. Unlike metal electrodes which are susceptible to corrosion, oxidation, and limited flexibility, nanocellulose exhibits superior flexibility, and corrosion resistance [120]. Moreover, its electrical conductivity can be further improved through physical or chemical modifications. Leveraging the diverse physicochemical characteristics and significant application potential of materials, researchers are developing high-performance cellulose-based TENGs using various fabrication approaches. Electrospinning cellulose-based film is characterized by high specific surface area, intrinsic surface roughness, and multilayered porous architecture, effectively increasing the triboelectric contact area at the microscopic scale [162]. The enhancement leads to greater surface charge density and improved energy conversion efficiency in TENGs. Widely adopted in TENG construction, electrospinning cellulose-based film offers viable pathways toward self-powered solutions for wearable electronics, sensors, smart textiles, and related fields [163]. TENG consists of substrate materials, triboelectric layers, and electrode materials. The substrate provides structural support. Triboelectric material generates triboelectric charges. The electrode facilitates the induction of charges. Theoretically, any two materials with different electron affinities can be selected as triboelectric layers. In current research, cellulose-based composites are predominantly employed as both the triboelectric layers and the substrate/support materials for TENGs. Regarding electrode selection, metallic materials are commonly used in TENG devices due to their exceptional electrical conductivity [22]. However, metal electrodes are prone to corrosion and oxidation, and once damaged, they cannot be regenerated. These issues limit the long-term stability and sustainability of TENGs. By introducing conductive nanomaterials, the electrical conductivity of cellulose-based materials is significantly enhanced, enabling them to serve as effective electrode or triboelectric layer materials for TENGs [164]. Sun et al. [165] fabricated a non-polarized polyvinyl alcohol (PVA)/cellulose composite for application in high-output piezoelectric nanogenerator and TENG. The PVA/cellulose-based PENG demonstrates Isc of 2.08 mA, Voc of 0.91 V, and power output of 1.89 mW. It enables it to directly illuminate eight LEDs and successfully power a calculator. Furthermore, the PVA/cellulose-based TENG exhibits excellent thermal stability and mechanical properties (Fig. 7a). Hu et al. [10] fabricated a biomimetic transparent tough CNF-based composite aerogel with a layered nanostructure through vacuum-assisted self-assembly followed by ambient pressure drying. The aerogel exhibits an attractive combination of excellent thermal insulation and a broad operating temperature range (− 196–230 °C) (Fig. 7b). This work presents a promising pathway for developing transparent, tough, and porous materials for applications in energy conversion and harvesting, TM, sensors, and related fields. Overall, the type of composite is biodegradability, high strength, and ease of cleaning. It offered broad application prospects in energy harvesting and TM.

Cellulose-based TENG materials encompass (but are not limited to) paper, films, and cellulose textiles [166]. The primary fabrication methods for composites can be classified into wet spinning, melt spinning, coating, and similar techniques. Among solution spinning techniques, wet spinning stands out as the simplest and most efficient method [167]. The polymer solution is quantitatively extruded through a spinneret, after which the fine solution streams are solidified into fibers via a coagulation bath, hot air, or hot inert gas. As one of the most advanced manufacturing technologies, it offers advantages such as low cost, strong operability, high manufacturing efficiency, and excellent scalability. It has been widely applied in the field of fiber production. Wet spinning serves as an effective approach for producing fibers with diverse structures and properties through precise control of spinning conditions [168]. The material is dissolved in a solvent, extruded through a spinneret. It is introduced into a coagulation bath to form fibers. By adjusting parameters of the spinning solution, fibers with varying diameters and morphologies can be fabricated, thereby meeting the diverse requirements of cellulose-based TENGs. Wang et al. [169] grafted Ti_3_C_2_T_x_/MoS_2_ onto cellulose diacetate using tetrakis (ethoxysilyl) silane, followed by the fabrication of nanofiber films via electrospinning. When paired with negatively charged materials, the resulting device achieves an optimal balance between low pressure drop (52 Pa) and high filtration efficiency (98.72%). The Voc of the composite initially exhibits a slight increase with rising temperature, followed by a subsequent decline (Fig. 7c). This phenomenon may be attributed to the accelerated electron transfer rate and enhanced evaporation of water molecules induced by elevated temperatures, which consequently suppresses charge transfer. Furthermore, temperature elevation raises the electron energy levels of the material. It increased the probability of high-energy electrons escaping from potential wells, thereby enhancing the output performance. Yu et al. [170] fabricated a PPy/natural chitosan/phytic acid material on Lycra fabric, assembling a biodegradable and flame-retardant SE-TENG (LPCP-TENG). The resulting thermal-resistant LPCP-TENG generates Voc of 0.3 V at an operational frequency of 5 Hz under a contact pressure of 2 N. The composite exhibits limiting oxygen index (LOI) of 35.2% (Fig. 7d). With the continuous advancement of spinning technologies, the efficient production of wet-spun fibers with large surface areas, high surface roughness, and complex structures remains a significant challenge. The spinning process is prone to spinneret clogging, rendering the fabrication procedure time-consuming, labor-intensive, and costly, thus necessitating further optimization. The electrospinning process requires control over numerous parameters. Even minor variations in spinning conditions can lead to structural defects and performance inconsistencies in the resulting ultrafine fibers. Factors such as the viscosity and concentration of the spinning solution, as well as the pH, temperature, and concentration of the coagulation bath, all influence formation of films [167]. In summary, electrospinning and wet-spun technology exhibit broad applicability to diverse materials. By adjusting the composition of the spinning solution and process parameters, cellulose-based films with different materials, morphologies, and functions can be prepared [171].

Additionally, coating is a method for preparing shaped fibers by applying a solution mixture to the fibers and curing it through temperature, chemical, or light exposure [172]. This approach was applied in high-efficiency, low-carbon, and multifunctional integrated applications because of its simplicity of operation. Chen et al. [173] designed a superhydrophobic conductive bacterial cellulose (BC) fiber, comprising a core layer formed by twisting CNT-impregnated BC and a shell layer coated with a double silica structure (Fig. 7e). This configuration enables the generation of an exceptional Voc (~ 266.0 V). Nevertheless, coating techniques still present notable limitations. Moreover, the underlying principles governing microstructural evolution during the thermal drawing fiber formation process remain inadequately elucidated, thereby impeding the enhancement of TENG functionality. Li et al. [174] designed a multifunctional TENG that integrates bidirectional thermal regulation and hydrophobic protection. The device employs a thermoplastic TPU/tetrabutylammonium hexafluorophosphate dendritically structured nanofiber film. As the positive electrode, it could achieve efficient photothermal conversion (Fig. 7f). The TENG can flexibly switch between thermal regulation modes (cooling and heating) according to ambient temperature. The integration of cellulose composites with TENG, incorporating TM as a core functionality, creates a “self-powered, thermally-manageable, wearable/implantable intelligent system” with fundamental value. Looking ahead, researchers could endeavor to develop all-cellulose-based TENGs, comprising cellulose-derived substrates, triboelectric layers, electrodes, and encapsulation layers. Integrating these with environmentally benign TM materials would substantially alleviate the issue of electronic waste. The approach also represents a significant trend in the evolution of flexible electronics and sustainable technologies.

### Carbon Nanotube

**Fig. 8:**
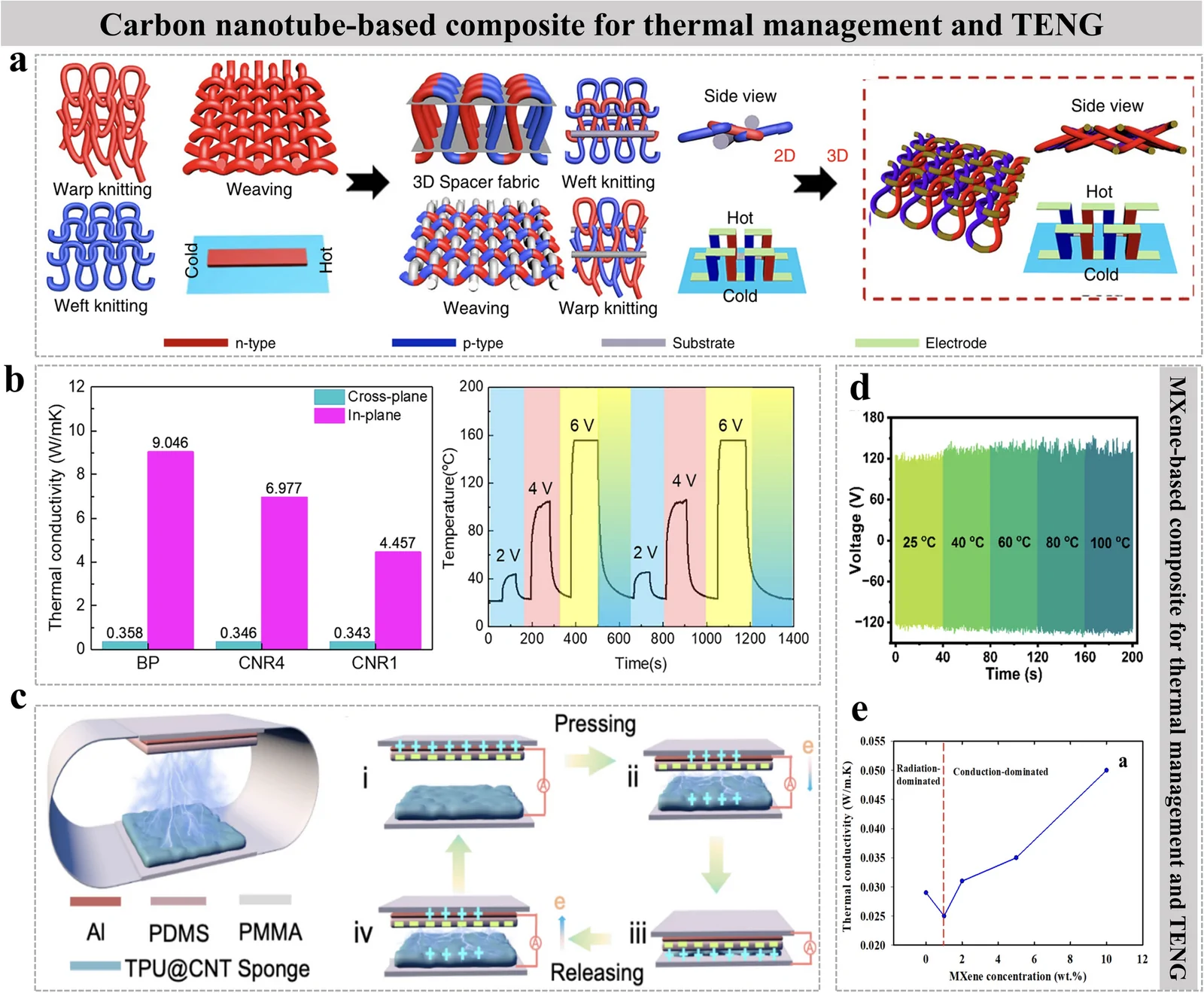
TM-TENG applications of CNT-based and MXene-based composites. **a** Comparison with other textile-based mode for TENGs [177]. Copyright 2020, Springer Nature. **b** Thermal conductivity of the composites (left). Heating cycles at applied voltages of 2, 4, and 6 V (right) [179]. Copyright 2022, Elsevier Ltd. **c** Schematic of the preparation of TENG device using the TPU@CNT sponge [180]. Copyright 2022, Elsevier Ltd. **d** Thermal stability response of optimized device with W-MX500 [94]. Copyright 2024, Elsevier Ltd. **e** Thermal conductivity curve of the 3D-printed CS/MXene aerogels [194]. Copyright 2025, Elsevier Ltd

CNTs have garnered significant attention due to their durability, flexibility, and outstanding mechanical and thermal properties [175]. CNTs exhibit exceptional thermal conductivity. When forming 3D thermal conduction networks within composites, it could efficiently conduct and dissipate thermal away from localized hot spots. CNT films or networks serve as ideal flexible and stretchable electrodes for TENGs [176]. Simultaneously, it can also function as highly efficient triboelectric layers with propensity for facile electron gain and loss. The incorporation of CNTs significantly enhances the strength, toughness, and fatigue resistance of the polymer matrix, which is crucial for the durability of wearable and flexible devices. Sun et al. [177] fabricated segmented thermoelectric yarns and corresponding textiles using CNT yarns. The resulting thermoelectric fabric achieves a peak power density of up to 70 mW m^−2^ under temperature difference of 44 K. The thermoelectric textile shows potential for applications in sensing, energy harvesting, and TM. The current thermoelectric yarns still fall short of meeting the requirements for industrial-scale loom weaving. However, CNTs are prone to agglomeration within the aerogel matrix, leading to the degradation of the aerogel scaffold. With increasing friction cycles, CNTs tend to detach, resulting in performance deterioration [178]. In comparison with other textile-based thermoelectric generator (TEG) configurations, the directly woven 3D thermoelectric units form self-supporting 3D structures driven by elastic forces, rather than relying on external substrates (Fig. 8a). Both mechanical aerogel and CNTs exhibit weak contact charging properties, necessitating further modification or composites to serve as electrodes for TENG applications. Fan et al. [179] developed a multifunctional natural rubber-toughened CNT composite paper (NR-BP) through vacuum filtration technique using CNT/NR hybrid dispersion. The NR-BP composite not only exhibits excellent EMI shielding properties but also demonstrates notable thermal conductivity. It was applied in Joule heating and flexible electrodes for TENGs (Fig. 8b, left). Moreover, the CNR4 maintains regular and stable temperature profiles under varying applied voltages, indicating remarkable cycling stability (Fig. 8b, right). This study enables the preparation of large-area samples exceeding A4 size through a simple vacuum filtration method, featuring the efficient and easily scalable process. Currently, much of the research in this field aims to precisely regulate interfacial interactions through covalent and non-covalent bonding strategies. The composites can reduce damage to CNT thermal conductivity while ensuring effective stress transfer and interfacial thermal conduction.

Under complex operating conditions, the stability of CNT networks and interfaces presents a key challenge. Wang et al. [180] fabricated a TENG by pairing a salt-templated CNT-decorated TPU@CNT sponge, which served as the electron-donor material. With a micro-structured PDMS film, it leveraged dual-contact electrification effect. Under 100 MΩ load, the TENG achieved Voc of ~ 252 V, Isc of ~ 38 μA, and power density of 1.642 W m^−2^. The surface convex–concave structures of the two tribo-layer materials are mutually corresponding. Initial charges are generated upon contact. During the compression stage, the deformation of the pore walls induces contact between the internally exposed CNTs and the TPU matrix. The contact triggers a secondary charge transfer, which significantly enhances the TENG’s output performance. The CNTs act as charge carriers, while the interfacial adhesion is strengthened by hydrogen bonding between their surface hydroxyl/carboxyl groups and the TPU chains [181]. The two aspects collectively contribute to the durable and robust performance of the TENG. The complementary microstructures on the opposing tribological surfaces enable initial charge generation upon contact. During compression, pore wall deformation forces the embedded CNTs to contact the TPU matrix, thereby triggering a secondary charge transfer that significantly boosts the TENG’s output. In this system, CNTs serve as charge carriers. Its surface hydroxyl and carboxyl groups enhance interfacial adhesion via hydrogen bonding with TPU chains. The synergy of these two mechanisms ensures the device’s overall durability and robust performance (Fig. 8c). The CNT-TENG generates pulsed alternating current characterized by high voltage and low frequency, posing a challenge for its efficient application in driving TM units. Although oxidation can enhance charge transfer efficiency, some studies indicate that it may concurrently degrade the material’s triboelectric properties, suggesting that its drawbacks could potentially outweigh the benefits. Particularly in materials like CNT and GO, oxidation tends to reduce electrical conductivity. It impaired the charge transport efficiency within the conduction layer.

CNT exhibits broad-spectrum, high-efficiency light absorption capabilities ranging from ultraviolet to infrared wavelengths. In the field of TM, the black coloration indeed presents a significant advantage. It implies a wide absorption spectrum and exceptionally high absorption rate within the visible band, enabling the capture of most sunlight and its conversion into thermal energy [182]. However, the selection of TM materials must never be based solely on color. MXene exhibit high electrical conductivity and promising thermal conductivity due to their strong in-plane bonding and metallic or semimetallic electronic structure [183]. It is not something that can be achieved with dark-colored materials alone. The choice of TM-TENG materials requires in-depth investigation of their core physical parameters, such as thermal conductivity, absorption/emission spectra, specific thermal capacity, and electrothermal efficiency. While color can serve as a preliminary visual indicator, it should never be used as a criterion for judging performance. Owing to the unique and excellent multi-angular performance in electrical conduction, thermal conduction, and mechanical properties, CNT composites hold profound potential for development in the integration of TM and TENG [184]. However, when high-thermal-conductivity fillers are poorly dispersed or used at excessive concentrations to enhance thermal pathways, they may form charge leakage channels within the triboelectric layer, thereby reducing its surface charge retention capability. In the future, precise manipulation of CNT arrangement and interfaces at the microscopic scale, coupled with the development of groundbreaking composite systems and integrated architectures, will fully utilize their theoretical potential. It will collectively drive the advancement of multifunctional, adaptive, and intelligent next-generation electronic systems.

### MXene

The emergence of 2D materials and extensive research on their mechanical, electrical, dielectric, and charge transfer properties [84, 185], have accelerated the development of 2D material-based TENG. It is crucial for building the next generation of mechanically robust TENG to explore innovative 2D materials with multifunctional attributes [186]. MXene represent a class of emerging 2D inorganic materials, broadly defined as a family of nanosheets comprising transition metal carbides, nitrides, and carbonitrides [187]. They are typically synthesized by selectively etching the A-layer elements from MAX phases (e.g., Ti_2_AlC, Ti_3_AlC_2_, Ti_4_AlC_3_). MXene exhibits rich surface chemistry, notable mechanical stability, hydrophilicity, high electrical conductivity (5,000 ≤ *σ* ≤ 10,000 S cm^−1^), and high charge carrier density [188]. These exceptional characteristics originate from their metal-like high free electron density and the lamellar structure of individual nanosheets. Furthermore, MXene can significantly enhance the dielectric constant of composites. The dielectric constant is a key parameter directly governing the surface charge density. The incorporation of MXene leads to a marked improvement in both charge density and maximum surface potential [189]. Charge density is a primary factor controlling power output. The addition of MXene effectively augments the overall electrical performance. The robust and versatile mechanical properties of MXene enable the design of flexible yet durable device architectures. When these attributes are synergistically combined, they contribute to a new generation of nanogenerators characterized by superior flexibility, transparency, cycling stability, and power output. It is noteworthy that Ti_3_C_2_T_*x*_ was the first MXene synthesized within this family of materials, as reported in the literature [190]. The majority research on MXene-based TENG devices has utilized Ti_3_C_2_. It is a material whose synthesis, properties, and applications have been extensively investigated. Triboelectric output parameters have been systematically studied using Ti_3_C_2_ surfaces modified with nitrogen and amino functional groups to evaluate their impact on TENG performance [187]. It is typically achieved by incorporating the chemically modified MXene fillers into their respective polymer matrices via composite fabrication methods. The surface potential of the triboelectric layer can be enhanced toward the desired positive or negative direction. Further intensification of the triboelectric effect can be achieved through electrode polarization of the composite film [191]. Owing to its distinctive surface chemistry, MXene has high electronegativity and rapid thermal dissipation performance. MXene significantly improves the ability of triboelectric materials to either gain or lose electrons, thereby substantially boosting the output performance of TENGs [192]. This characteristic makes MXene a promising candidate for use as a flexible electrode material in TENGs [193].

Furthermore, because of the presence of oxygen and abundant fluorine in terminal functional groups, MXene exhibits a stronger triboelectric negativity than PTFE. It positioned it as an ideal negative triboelectric material for TENG applications. Anwer et al. [94] developed a unique electrode design for a CS-TENG, which utilizes a multilayer Ti structure as the negative triboelectric layer. The innovative synthesis strategy creates a composite triboelectric layer composed of MXene/layered titanium dioxide superstructure/PVA. After systematic optimization of both triboelectric layers, the resulting TENG demonstrates outstanding performance, including Voc (~ 120 V), Isc (~ 25 μA), and Qsc (~ 5.66 W m^−2^). Furthermore, the device exhibits excellent thermal stability, maintaining its functionality across a temperature range from 25 to 100 °C (Fig. 8d). Jalali et al. [194] fabricated a composite aerogel via direct ink writing of 3D-printed CS/MXene ink. The aerogel exhibits significant performance enhancement. The Voc increases from 22 V for the pure CS aerogel to 110 V with the incorporation of 2 wt% MXene. The thermal conductivity of the composite aerogel initially decreases but subsequently increases with further increments in MXene concentration (Fig. 8e). The decrease in the thermal conductivity of the CS/MXene aerogel at low MXene loadings is attributed to insufficient interconnection of MXene particles. It disrupts the primary thermal conduction pathways and enhances phonon scattering. As the concentration increases, the formation of effective percolation channels and improved conductive networks enhance thermal transfer, leading to a subsequent rise in thermal conductivity. Furthermore, MXene can dampen thermal vibrations and reduce phonon scattering, which also contributes to the overall thermal conduction performance. This study highlights the significant potential of the printed aerogel for applications in energy harvesting, EMI shielding, and thermal insulation. The porous aerogel structure exhibits remarkable flexibility and compression resilience, enabling it to withstand repeated mechanical deformation. The innumerable micro- and nanoscale pores within the aerogel provide a vastly enlarged effective contact area for the contact-separation dynamics between triboelectric layers. As a superior thermal insulator, the aerogel ensures that within a heating system constructed from it, thermal is not readily dissipated. Consequently, aerogel-based TENG can effectively focus thermal within a targeted zone, significantly enhancing the efficiency and precision of TM [86]. Furthermore, MXene possesses intrinsic metal-like high electrical conductivity. When assembled into a 3D aerogel architecture, the porous network functions as highly efficient electrode. The unique characteristic enables precise and programmable control over the heating temperature through simple modulation of the TENG’s operational status [195].

### Liquid Metal

**Fig. 9:**
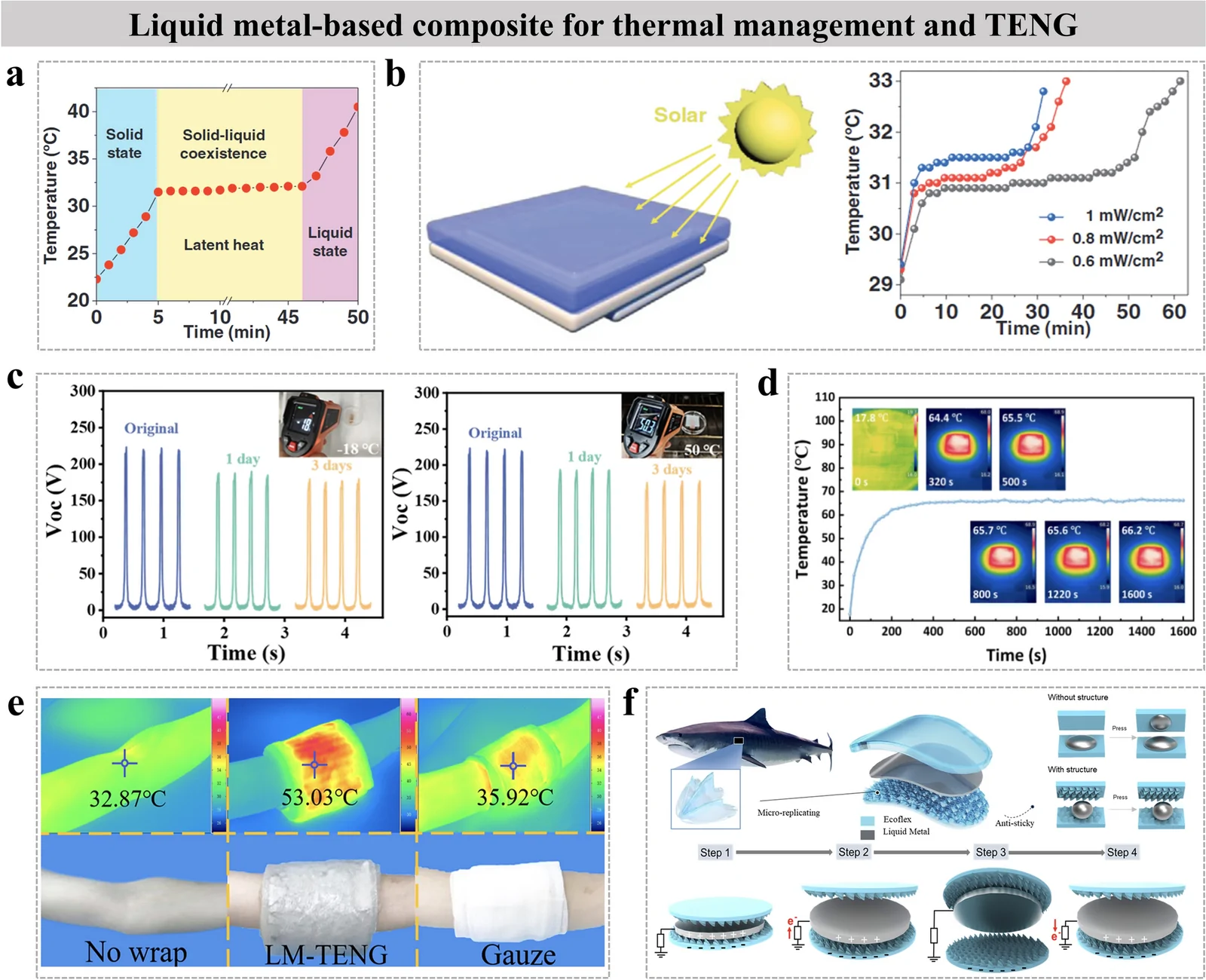
TM-TENG applications of LM-based composites. **a** Temperature changes of solid Galinstan in a constant 50 °C water bath [199]. Copyright 2021, Wiley–VCH. **b** A solar simulator was used to illuminate the TE-skin (left). The effect of illumination intensity on the temperature changes of TE-skin (right) [199]. Copyright 2021, Wiley–VCH. **c** Changes Voc of after 3 days of storing CPL-TENG at 18 and 50 °C [200]. Copyright 2023, Springer Nature. **d** Temperature stability of HPCPC at 1000 μW cm^−2^ light intensity [126]. Copyright 2025, Elsevier Ltd. **e** Thermal therapy effect of LM-TENG on the patient’s arm [202]. Copyright 2023, Elsevier Ltd. **f** Schematic structure of bioinspired shark skin-based gait sensor with liquid metal (up). Mechanism related to contact electrification and electrostatic induction for the bioinspired TENG (down) [205]. Copyright 2022, Elsevier Ltd

Liquid metal (LM) has been introduced into triboelectric materials, forming an unconventional category of LM-based TENGs [92]. It has electrical conductivity, deformability, and low toxicity. LM uniquely combines the deformation capabilities of a liquid with the characteristic properties of a metal, making it an ideal material for fabricating stretchable, foldable, and even self-healing electrodes [196]. When integrated with other materials, LM can not only enhance the composite’s performance but also mitigate its inherent limitations. A critical consideration in this integration process is achieving effective compatibility between the LM and the host matrix. Metallic particles exhibit excellent thermal conductivity, yet their high electrical conductivity can severely compromise the electrical insulation required for effective triboelectric layers. Conversely, ideal insulating materials often suffer from poor thermal conductivity. LM exhibits a dual functional role, serving effectively as both a triboelectric material and an electrode [197]. Its incorporation into TENGs offers a twofold application strategy. It can be utilized to enhance the electronegativity of the triboelectric layer. LM-based composite TENG could improve the electrical conductivity of the composite, thereby collectively boosting the overall output performance of the TENG [198]. Xiang et al. [199] reported a phase change material (PCM)-based temperature-regulating electronic skin (TE-skin) incorporating liquid metal, with a melting point tuned to within the comfortable range of human skin temperature. The TE-skin can dynamically regulate its thermal properties through phase transition in response to ambient temperature changes. Figure 9a displays the temperature profile of solid Galinstan immersed in a constant 50 °C water bath. It can be observed that the temperature remained constant at 31.5 °C for 40 min due to the phase change phenomenon. In daily scenarios, heating effects are often induced by sunlight. This study employed a solar simulator to investigate the thermal regulation capability of the TE-skin under light illumination. When light intensity increases from 0.6 to 1 mW cm^−2^, the equilibrium temperature rises, while the corresponding duration decreases (Fig. 9b). It indicates that higher solar irradiance increases the temperature of the entire TE device and accelerates internal phase transitions. This result is similar to the temperature rise observed in thin-film heaters. To test the thermal stability of this TENG, it was placed in low-temperature (− 18 °C) and high-temperature (50 °C) environments. Results showed that after 3 days of storage at 18 and 50 °C, respectively, the Voc of the TENG remained at 179.90 and 177.96 V (Fig. 9c). Due to the endothermic effect of gallium-based LM itself, LM-TENG exhibits corresponding phase change capabilities [200]. LM incorporated into TENG exhibits excellent electrical conductivity or enhances the material’s electronegativity without altering its inherent mechanical properties.

As leading global producer of LM, China benefits from the material’s significantly lower cost compared to alternatives, such as silver nanowires, GO, and MXene [201]. This cost advantage greatly facilitates the large-scale application of high-performance TENG. The LM-based TENG is a device that converts mechanical energy into electrical energy. It has already found application in harvesting low-frequency mechanical energy from various sources, including human motion, environmental vibrations, water droplets, and ocean waves. Liu et al. [126] developed multifunctional textile-based TENG, composed of PDA/polyaniline/CNT/ dodecyltrimethoxysilane (HPCPC-TENG). The HPCPC-TENG exhibits exceptional photothermal conversion performance, as illustrated in Fig. 9d. Furthermore, the TENG device constructed with the material achieves Voc of 200 V, Isc of 32 μA, and power density of 2.5 W m^−2^. The HPCPC composite reaches its saturation temperature after approximately 300 s of light exposure, with the surface temperature rising by about 48 °C. It ultimately stabilizes at approximately 65.5 °C and maintains this temperature steadily for about 1300 s. Gong et al. [202] prepared multifunctional LM-based TENG device. Under applied force and frequency of 40 N and 15 Hz, the LM-TENG achieves Voc of 22.29 V and power output of 55.16 µW across 9 MΩ load. Furthermore, the device demonstrates excellent electrothermal performance under 1.5 V. The composite surface temperature raised to 69.02 °C. When 10 V bias is applied, the smart patch attains the melting point of the LM and maintains a stable temperature of approximately 42.74 °C. It falls precisely within the optimal range for thermotherapy applications (Fig. 9e). Nevertheless, LM-TENG still faces challenges such as potential liquid metal leakage and limited output performance. For application in stretchable and soft electronics, LM must be precisely patterned. The primary techniques for LM patterning can be broadly categorized into three approaches, such as deposition through masks, imprinting methods, and direct patterning [203]. Mask-assisted deposition enables the creation of intricate patterns with micron-level or even higher resolution, making it suitable for applications with stringent precision requirements, such as integrated circuits. On the other hand, printing methods feature relatively simple processes and lower equipment costs [204]. As an additive manufacturing technique based on on-demand dispensing, they minimize material waste and allow for the printing of multilayered structures. Direct patterning eliminates the need for mask fabrication or plate engraving, significantly shortening the cycle from design to the composites, making it an ideal choice for achieving design-to-manufacturing integration. Yeh et al. [205] developed a self-powered, flexible wearable sensor based on LM-TENG. LM is encapsulated within an innovative shark skin-inspired microstructure and embedded in the surface of an Ecoflex substrate (Fig. 9f). The unique surface morphology of this sensor imparts hydrophobicity to the solid triboelectric layer while enabling highly sensitive real-time signal monitoring and long-term stability. When the micro-structured surface contacts with LM, negative and positive charges are generated on LM surfaces. To re-establish electrostatic equilibrium, free electrons flow from the ground through the external circuit toward LM. Upon complete separation, a reverse electron flow occurs as the micro-structured surface re-approaches the LM. However, elements such as gallium and indium are classified as rare metals, resulting in relatively high material costs. Although gallium-based alloys generally exhibit low toxicity, their long-term biosafety and comprehensive biocompatibility upon sustained contact with biological tissues require further thorough investigation [206]. In the future, breakthroughs in LM-TENG technology will rely on synergistic innovations in composite formulations, microfluidic technology, and system-level energy integration. The ultimate objective is to realize next-generation electronic systems that are genuinely self-powered and self-cooling, mirroring the efficient autonomy found in living organisms.

### Phase Change Materials

**Fig. 10:**
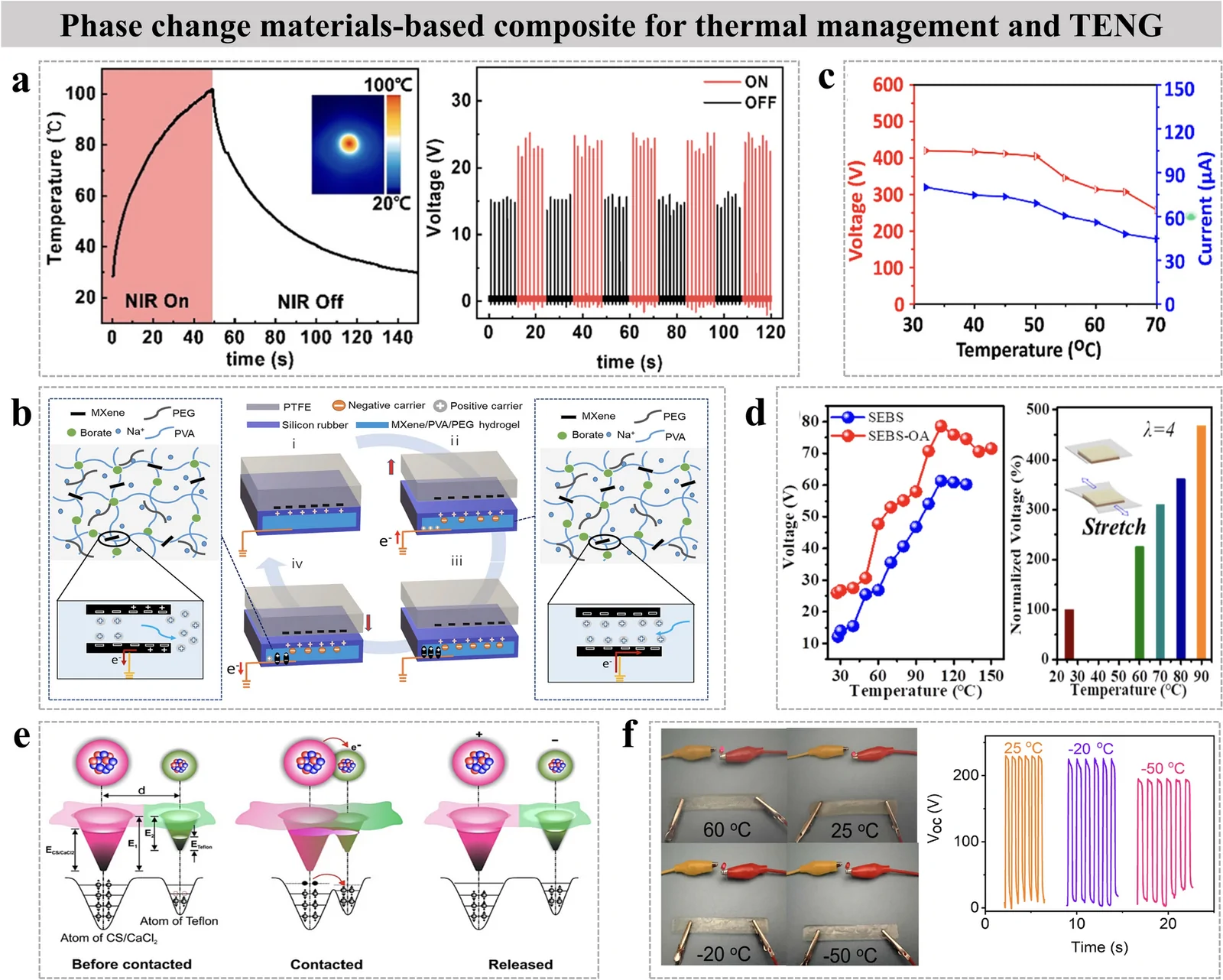
TM-TENG applications of PCM-based composites. **a** Temperature change processes of PRS with Fe_3_O_4_ NPs content of 7 wt% under NIR irradiation. The inset is the infrared image at 50 s [134]. Copyright 2024, Elsevier Ltd. **b** SVP mechanism illustrates how compressive stress induces a positive charge flow into the Ti_3_C_2_ MXene microchannel [214]. Copyright 2024, Elsevier Ltd. **c** Electrical output behavior of PVA/PEG-TENG under varying thermal environment [217]. Copyright 2025, Elsevier Ltd. **d** Thermal-related output voltage of SEBS and SEBS-OA-based TENGs (left). The corresponding output voltage of SEBS-OA-based TENG when the *λ* = 4 and at different operating temperatures (right) [217]. Copyright 2023, Elsevier Ltd. **e** Atomic-scale-electron-cloud-potential model diagram for the CS/CaCl_2_ and Teflon before, after release, and contact moment of electrification [220]. Copyright 2022, Elsevier Ltd. **f** Pictures of lighting LED bulbs at different extremes of temperatures (left) [221]. Copyright 2025, Elsevier Ltd

PCMs could store and release thermal energy through reversible phase transitions [73]. They can be classified based on their chemical composition, phase change mechanism, and transition temperature (*Tg*). According to chemical composition, PCMs are categorized into single-component PCMs (e.g., inorganic and organic PCMs) and composite PCMs [207]. However, the low thermal conductivity of organic PCMs limits their application in scenarios requiring rapid thermal transfer. During the process of absorbing thermal and transitioning from solid to liquid, PCMs are prone to leakage, which can pose environmental concerns [208]. Encapsulation of PCMs serves as an effective strategy to mitigate the mentioned issues. Microencapsulated phase change materials (MEPCMs) with a core–shell structure can be fabricated by coating a thin, protective film around the phase change particles [209]. Compared to pure PCMs, MEPCMs possess a larger specific surface area, which enhances thermal response speed and thermal transfer efficiency. It enabled more effective TES and temperature control. The fundamental functions of MEPCMs are categorized into temperature regulation and TES. The TES aims to utilize the stored thermal for practical applications [210]. The term “PCM/thermoelectric device” refers to a configuration where the PCM is positioned between the thermal source and the hot end of the thermoelectric device. Initially, the PCM exists in a solid state. As the temperature of the thermal source increases, the temperature of the PCM gradually rises and undergoes a phase transition from solid to liquid upon reaching its phase change temperature [211]. Owing to the capability of PCMs to store latent thermal during phase transition, substantial amount of thermal energy from the thermal source can be absorbed and stored within the PCM. Furthermore, the temperature of the PCM can remain stable near its melting point throughout the phase change process [212]. The liquid–solid TENG generates electrical power by harnessing energy from various water sources. Its operational principle is grounded in contact electrification and electrostatic induction, whereby a continuous electric current is generated through an external circuit as water repetitively contacts and separates from a solid surface. Yun et al. [134] proposed a self-powered photothermal detection system based on PR-TENG for monitoring light intensity and proximity. The temperature of the composite in the irradiated area increases from room temperature to the *T*m (~ 55 °C) of PW within 5 s. Solid-state PW melts into liquid, and upon deactivation of the light source with subsequent temperature decrease, it resolidifies back to the solid state (Fig. 10a, left). Due to the function of the composite as a triboelectric layer, alterations in its surface properties consequently influence the electrical output of the PR-TENG. Figure 10a, right illustrates the switching performance of the PR-TENG upon contact with a water droplet and its corresponding impact on the Voc. However, a critical factor for the technical viability lies in ensuring the robust integration of the PCM with the electrode material throughout repeated solid–liquid phase transitions, thereby preventing any leakage [213]. Current strategies to mitigate leakage primarily employ materials engineering approaches, such as microencapsulation and the construction of 3D porous support networks (e.g., aerogels), to address this challenge. Lai et al. [214] fabricated PVA/Ti_3_C_2_/PEG composite that significantly enhances the triboelectric output performance of the TENG. 3-chloropropyltrimethoxysilane and 1H,1H,2H,2H-perfluorooctyltrimethoxysilane were employed to modify the surface of MXene for the fabrication of the hydrogel-TENG (Fig. 10b). The resulting TENG demonstrates a notable Voc of approximately 212 V and exhibits high sensitivity to mechanical stimuli. It made it a suitable candidate for integration into motion sensors designed for monitoring human body movement. However, regarding PCMs as representative TM materials, the research focus within the TENG field does not appear to be primarily on the TES capabilities. The primary objective of integrating PCMs into TENGs is often to leverage their endothermic properties for device cooling. By preventing device overheating, PCMs indirectly ensure the reliable operation and extended service life of the TENG [134]. Although it is theoretically feasible to harvest the frictional thermal generated during TENG operation or environmental thermal energy, the thermal generated by friction in TENGs is relatively dispersed. Compared to dedicated TEG or photothermal conversion systems, the direct and efficient harvesting and conversion of thermal into other usable forms of energy currently faces potential efficiency challenges [215]. Consequently, researchers may therefore prioritize addressing the more immediate issue of device TM.

For thermoelectric-phase change coupled systems with the “PCM/thermoelectric device” configuration, relevant research has substantiated their application potential in waste thermal recovery. The incorporation of PCMs with high energy storage density will further amplify the technical advantages of this configuration. The conventional strategy for TM materials utilizes porous structures to reduce density for enhanced thermal insulation. It is constrained by the bottleneck in effectively modulating thermal diffusivity. By augmenting thermal capacity, TM-TENG materials enable the synergistic optimization of both thermal conductivity and thermal diffusivity, thereby offering a pioneering technical pathway to address the existing bottleneck in thermal modulation [199]. The introduction of PCMs into the positive electrode materials of TENGs signifies an evolution within this field, transitioning from solely mechanical energy harvesting toward intelligent, adaptive, and multifunctional integration. The incorporation of PCMs may impart complex effects on the flexibility, weight, and triboelectric output of TENGs [208]. The ability to absorb and release substantial latent thermal during phase transitions can effectively mitigate temperature fluctuations during TENG operation, consequently enhancing device longevity. Amini et al. [216] fabricated a high-performance TENG based on PEG/PVA. The introduction of PEG establishes additional polar functional groups (–OH), which form a robust hydrogen bonding network with PVA and create abundant sites for charge interactions. The investigation demonstrates a remarkable enhancement in triboelectric output, achieving performance levels of 426.52 V and 82.65 μA, respectively. As the temperature varies from 30 to 70 °C, both the Voc and Isc remain nearly constant up to 50 °C but begin to decline at elevated temperatures. The phenomenon is attributed to the thermomechanical properties of PVA and PEG. At high temperatures, PEG undergoes softening, which reduces the rigidity of the composite. Concurrently, PVA experiences thermal degradation and increased chain segment mobility above 50 °C, thereby accelerating charge dissipation and leading to a deterioration in triboelectric performance (Fig. 10c). Furthermore, the PEG/PVA-TENG could power smartwatches, LED arrays, and commercial capacitors. Zhao et al. [217] fabricated a skin-like flexible film composed of SEBS/fatty acid (SEBS/FA), which exhibits tunable surface triboelectric properties and enhanced triboelectric output at elevated temperatures. The composite film demonstrates an exceptional output efficiency of up to 302.32% at 110 °C, maintaining performance above 100% even at 150 °C (Fig. 10d). The SEBS/FA-based TENG proves capable of effectively harvesting mechanical energy in high-temperature environments, demonstrating sufficient power generation for commercial capacitors and LEDs. Compared to organic PCMs represented by PW and fatty acids (FA), inorganic salt hydrates (SHs) demonstrate significant advantages across several key performance metrics. In terms of latent thermal of phase transition, SHs typically possess a substantially higher latent thermal capacity, which translates to a greater thermal storage capability per unit volume or mass [218]. SHs exhibit superior thermal conductivity and inherent non-flammability, which significantly enhances rapid thermal transfer, improves utilization efficiency, and ensures greater safety in TES systems. As a class of high-performance PCMs, SHs demonstrate considerable application potential in the field of TES [219]. This technology currently remains predominantly in the exploratory stages of laboratory research. While directly relevant literature still limited, existing studies clearly reveal its developmental progress and prominent advantages. Notably, preliminary investigations have explored the application of calcium chloride dihydrate specifically in TENG systems. Calcium chloride dihydrate can store and release thermal through phase transitions (changes in its chemical bonding state). Charoonsuk et al. [220] proposed a straightforward ion-embedding strategy. To enhance the electrical output performance of TENGs, by introducing additional surface charges onto cationic CS biopolymer substrates. Through precise modulation of the film surface roughness, the optimized composite achieved peak electrical outputs of approximately 149 V for Voc and 15 μA for Isc. Under an external load resistance of 10 MΩ, the maximum power density of the TENG reached 400 μW cm^−2^. The energy band diagram is presented in Fig. 10e. It was constructed based on bandgap values determined from UV–Vis measurements. This schematic illustrates the transfer of additional charged unoccupied electrons within the CS/CaCl_2_ complex during the contact electrification process. Due to its higher anionic molar ratio, the incorporation of CaCl_2_-containing electrolytes generates higher energy levels and enables a greater number of electron trap states near the lowest unoccupied molecular orbital of CS. During contact electrification, positive charges on the CS/CaCl_2_ surface are neutralized through electron outflow, while electrons accumulate on the Teflon surface, conferring a negative charge until the surface potentials of the two contacting materials reach equilibrium. The configuration facilitates enhanced electron flow, resulting in superior output performance after the introduction of the CaCl_2_ solid polymer electrolyte. This work provides valuable insights for advancing TENG development. However, calcium chloride dihydrate is generally not regarded as an ideal solid–liquid PCM, unlike PW or calcium chloride hexahydrate. The application scenarios and technical approach of TM-TENG also differ from traditional latent thermal storage materials, with its operating mechanism leaning more toward thermochemical thermal storage. Zhu et al. [221] proposed a one-pot strategy within a green deep eutectic solvent system. This system utilizes zinc chloride hydrate as the hydrogen bond acceptor and acrylic acid (AA) as the donor. This approach facilitates the dissolution of cellulose by disrupting the intrachain hydrogen bonds, thereby enabling the construction of complex coacervation. The LED bulb remains illuminated under both heating at 60 °C and sub-zero temperature conditions, demonstrating its excellent suitability as a soft electrode for TENGs (Fig. 10f, left). The deep eutectic gel-based TENG (CE-TENG) demonstrates the capability to harvest mechanical energy and convert it into stable electrical signals across various extreme temperatures, exhibiting Voc of 220 V at 25 °C and 180 V at − 50 °C (Fig. 10f, right). The consistent ability of the CE-TENG to power LED bulbs under different temperature conditions underscores its exceptional temperature resistance and TM performance. This work achieves synergistic breakthroughs across multiple performance metrics, setting it apart from numerous previously reported gel materials and providing an ideal soft electrode and sensing medium for flexible electronic devices.

Future development will no longer be confined to PCMs. It will place greater emphasis on the efficient integration of phase change-TENG devices into specific application systems. The approach offers an attractive solution for addressing the TM bottlenecks in PCM-TENG systems and expanding their energy harvesting dimensions. In application scenarios with stringent TM requirements, devices and systems that combine efficient TES/temperature regulation with stable electrical/thermal output will hold distinct advantages. The deep integration of TENG-PCM systems with energy storage units, low-power edge computing nodes, and artificial intelligence algorithms will enable the construction of intelligent IoT systems capable of real-time decision-making.

### Metal Organic Frameworks

**Fig. 11:**
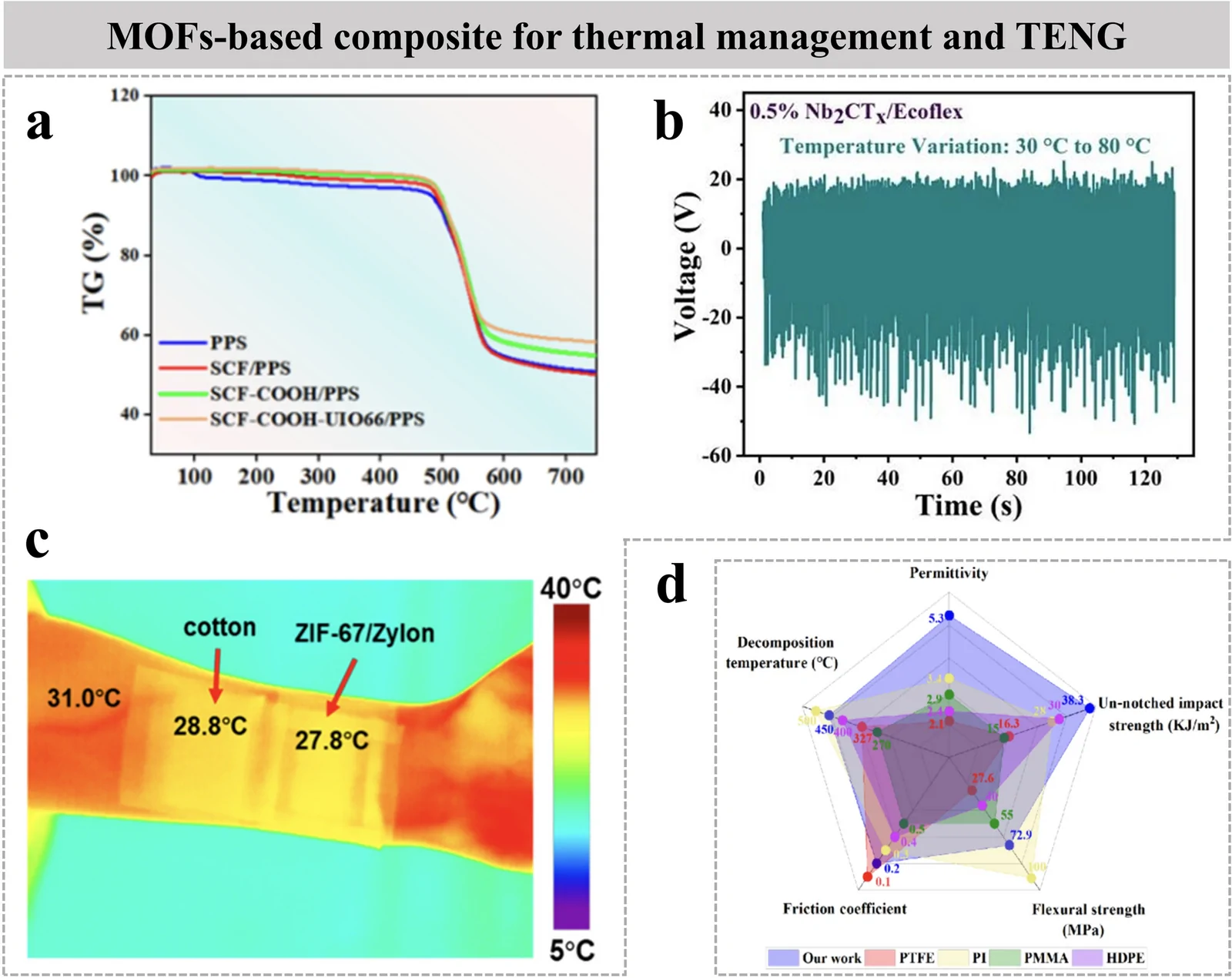
TM-TENG applications of MOF-based composites. **a** TG results of PPS composites [226]. Copyright 2024, Elsevier Ltd. **b** Stable Voc of MD-TENG under varied thermal environments [229]. Copyright 2025, Elsevier Ltd. **c** Infrared thermal imaging of ZIF-67/Zylon aerogel fabric and cotton fabric on human skin [137]. Copyright 2025, Elsevier Ltd. **d** Comparison of the properties of representative triboelectric polymers and LCP/PPS composites [235]. Copyright 2024, Elsevier Ltd

Metal–organic frameworks (MOFs) represent a class of 3D network complexes formed through the self-assembly of metal ions as nodes and organic ligands as linkers [222]. Initially proposed by Professor Yaghi and colleagues in 1995 [223], MOFs are an original category of porous crystalline materials constructed via coordination bonds between inorganic metal ions and organic ligands. While MOFs can crystallize into 3D structures, they can also form layered architectures. The presence of nanoscale porosity expansion and substantial internal surface area endows layered MOFs with exceptionally high specific surface areas [224]. These hierarchical MOFs can function as electron capture materials, making them suitable for application in triboelectric materials [225]. Compared to other porous materials, MOFs are widely utilized in TM, sensing, and TENG applications due to their distinct advantages, including high crystallinity and ease of chemical modification. Xu et al. [226] developed a TENG fabricated from a MOF-enhanced polyphenylene sulfide (PPS) composite and constructed on a short carbon fiber (SCF) substrate. Thermogravimetric analysis (TGA) was employed to characterize the SCF-COOH-UIO-66/PPS composite, revealing distinct melting, crystallization, and decomposition temperatures of 290.2, 445.9, and 233.5 °C, respectively (Fig. 11a). The composite exhibits exceptional thermal stability, high specific surface area, and a highly ordered porous structure. These characteristics collectively contribute to significantly enhanced thermal dissipation and thermal conductivity. The inherent porous architecture of MOFs, coupled with the interactions between metal ions and organic ligands, imparts a defined surface roughness to the material. During the cyclic contact and separation of the triboelectric layers, they are positive and negative electrostatic charges generated on the MOF surface. Additional charges are also induced on the pore surfaces due to the phenomenon of electrostatic induction [227]. The accumulated charge on the MOF surface increases the surface charge density of the friction material, thereby enhancing the output performance of the TENG [228]. Chittibabu et al. [229] fabricated a negative triboelectric layer using an MXene/Ecoflex composite (MD-TENG). The incorporation of MXene significantly enhances the dielectric properties, charge-trapping efficiency, and mechanical robustness of the nanocomposite. The TENG operates in a metal-dielectric CS-TENG to harvest energy from mechanical motion. The as-fabricated MD-TENG delivers a maximum power output of 15.35 μW, corresponding to a power density of 4.55 μW cm^−2^. During the temperature variation cycle (30–80 °C), the MD-TENG maintains stable Voc without any observable fluctuations or degradation across the entire range. The device demonstrates a consistently high-voltage output that is independent of operating temperature (Fig. 11b). This system represents the first integration of self-powered sensing with the assessment of sensory preferences in children with autism, providing a vital tool for noninvasive, home-based behavioral analysis.

Current research on composite electrodes integrating MOF materials with gels for TENGs primarily focuses on ZIF-type materials [230, 231]. However, the limited diversity of functional groups in their imidazolate ligands may result in insufficient interfacial bonding strength with the gel matrix. Conventional synthetic methodologies for MOFs mainly include hydrothermal, sonochemical, mechanochemical, and electrochemical approaches [232]. Hu et al. [137] fabricated a MOF-based composite aerogel consisting of 81 wt% ZIF-67. The composite demonstrates exceptional mechanical properties and outstanding thermal insulation performance (Fig. 11c). The ZIF-67/Zylon porous aerogel exhibits low thermal conductivity, coupled with excellent thermal retention characteristics as a fabric material. The aerogel fibers significantly reduce thermal conduction through the Knudsen effect, along with their abundant micropores and mesopores [233]. The flexible TENG was constructed using ZIF-67/Zylon hybrid aerogel fibers as the triboelectric layer. The device demonstrates exceptional electrical output, achieving Voc of 70.8 V and a power density of 108.6 mW m^−2^. In contrast, UIO-type MOF materials, containing abundant carboxylic acid groups, exhibit superior binding efficacy with ionic hydrogels [234]. Furthermore, conductive MOF materials can significantly enhance the current density in the transport layer through the synergistic interaction between metal centers and conductive ligands. Khandelwal et al. [225] investigated ZIF-7, ZIF-9, ZIF-11, and ZIF-12 as triboelectric materials for TENG applications. Among these, the ZIF-7-based TENG demonstrates the optimal output performance. Research on utilizing MOF materials as standalone TENG electrodes remains relatively limited, primarily due to their inherent weak electrical conductivity, which renders them less suitable for direct application as electrodes in TENG systems. Functionalization strategies can effectively enhance the polarity or modulate the electronegativity of MOF-based electrodes. The introduction of hydrophilic functional groups ensures superior interfacial compatibility between MOFs and other materials (such as hydroxyl and carboxyl groups) [226]. Furthermore, MOFs can serve as nanoscale “containers” for PCMs. Leveraging their ultrahigh specific surface area and tailorable pore structures, MOFs effectively confine molten PCMs through physical adsorption and capillary forces. It resolved the persistent issue of leakage during solid–liquid phase transitions in conventional PCM systems. However, the inherently low thermal conductivity of most MOFs restricts the overall thermal conductivity and charge–discharge rates of PCMs composites [134]. Even when compounded with highly thermally conductive materials to mitigate this limitation, the required processing introduces additional complexity and cost. Looking forward, the design of MOF composites that integrate high specific surface area with superior thermal and electrical conductivity will be crucial for overcoming existing performance bottlenecks.

### Others

Metal oxides possess intrinsically higher thermal conductivity than polymers [66]. Constructing 3D thermal conduction networks with them can enhance the overall thermal diffusion efficiency of composites. Particles are incorporated into polymer matrices to enhance TENG output by synergistically combining the advantages of both piezoelectric and triboelectric effects, such as zinc oxide (ZnO), strontium titanate (SrTiO_3_), zinc tin oxide (ZnSnO_3_), and barium titanate (BTO). Xu et al. [235] developed a composite incorporating ZnO-grafted CNT/RGO (AR-TENG). It significantly enhanced the interfacial bonding strength between PPS and liquid crystal polymer. The AR-TENG demonstrates reliable operational stability under elevated temperature and high humidity conditions, exhibiting moderate-to-high thermal stability that renders it suitable for a wide range of high-temperature applications (Fig. 11d). Surface modification of metal oxides further enhances the contact area between CNTs/GO nanocomposites and the matrix. The treatment promotes uniform dispersion of fillers throughout the overall system. Among these materials, BTO nanoparticles have attracted significant attention due to their combined piezoelectric and dielectric properties, as well as their cost-effectiveness. The incorporation of conductive fillers into triboelectric polymer matrices plays a critical role in enhancing the overall output performance of TENGs, as they provide micro-capacitors for storing additional electrons [236]. Porous metal oxide aerogels combine low thermal conductivity with high thermal emissivity, enabling them to block external thermal influx while enhancing thermal dissipation through infrared radiation. The dual-mode “thermal insulation–radiation” regulation makes them particularly suitable for thermal protection in TENGs under extreme temperature conditions [195]. It is noteworthy that excessively high filler content not only significantly increases the cost of composites but may also elevate material rigidity, thereby reducing triboelectric efficiency.

Covalent organic frameworks (COFs) are a class of crystalline porous organic materials constructed from light elements connected by robust covalent bonds. They exhibit characteristics such as high specific surface area, highly ordered structures, tunable pore channels, and designable functionalities, which endow them with significant potential in the fields of TM and TENGs. The combination of carbon atoms and other light elements via covalent interactions in COFs enables 2D-COFs to typically crystallize into layered structures. Consequently, the specific geometry of nodes and linkers in the topological diagram can be utilized to tailor the properties of layered COFs. Given that densely aligned π-columns and arrays on the framework can serve as pre-arranged pathways to facilitate charge transport, layered COFs offer an attractive structure–function platform for the fabrication of TENGs. Despite their considerable potential, research on COFs in the field of TM remains in its nascent stages. Key challenges currently include the generally low thermal conductivity of COF materials.

## Thermal Management Applications Based on TENGs

### Thermal Energy Harvesting

**Fig. 12:**
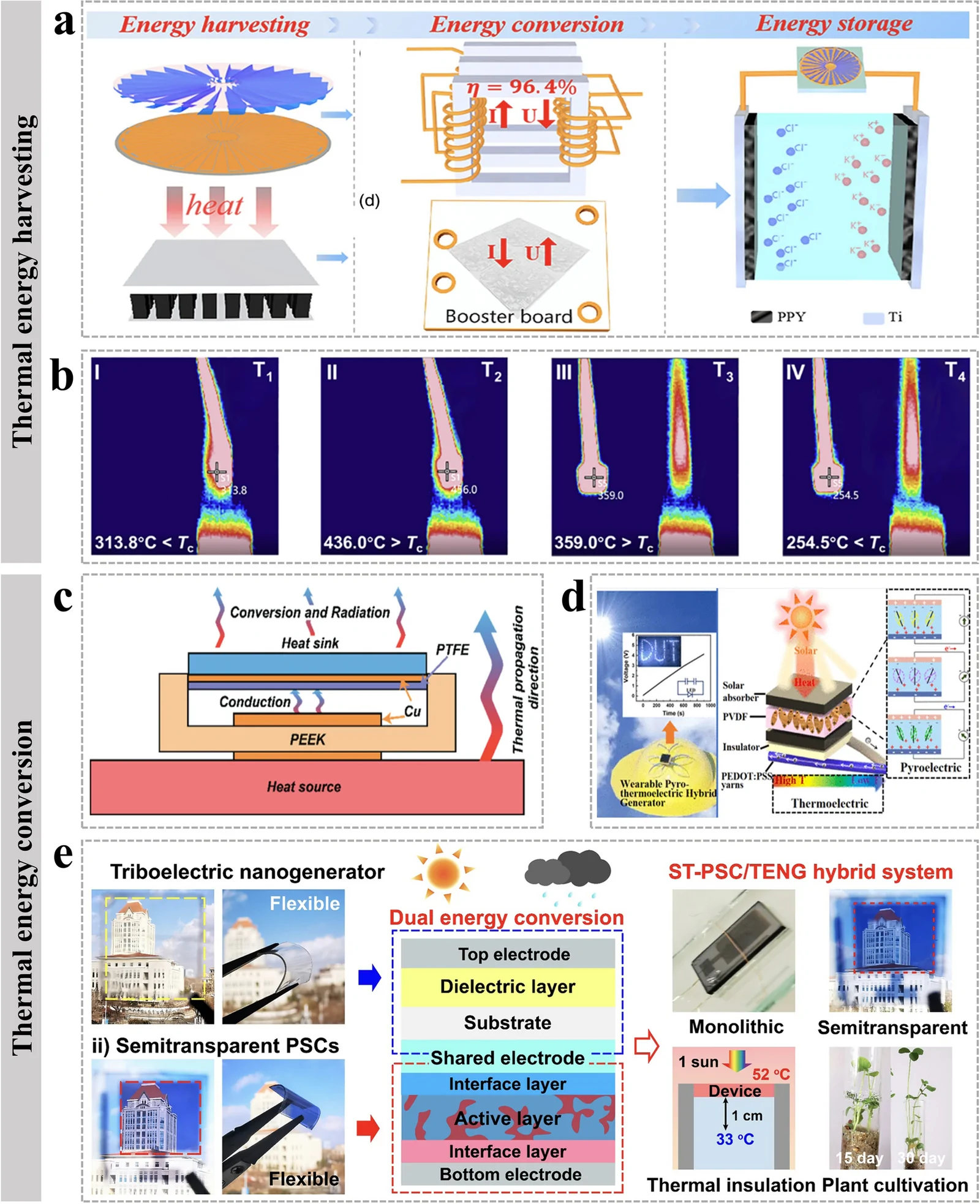
Thermal energy harvesting and conversion of the TENGs. **a** Schematic diagram of thermal transfer from AR-TENG to ThEG and energy storage in PPy-SC by hybrid device [239]. Copyright 2022, Wiley–VCH. **b** Thermal images of nickel blocks at different stages [240]. Copyright 2022, Elsevier Ltd. **c** Schematic diagram of main thermal transfer processes occurring in the TD-TENG [256]. Copyright 2022, Wiley–VCH. **d** Application diagram of a new wearable thermoelectric hybrid generator for solar energy collection [258]. Copyright 2022, American Chemical Society. **e** Schematic illustration and digital photographs of the ST-PSC/TENG hybrid systems developed [259]. Copyright 2022, Elsevier Ltd

Conventional TENG solely harvests mechanical energy, while thermal energy is often dissipated as waste thermal. Thermal energy exists in diverse forms across various environments [237]. However, devices currently employed for low-grade waste thermal harvesting typically rely on toxic or scarce materials, posing significant environmental and economic challenges. The inherent toxicity and limited availability of these materials hinder their widespread adoption. To advance toward sustainable and eco-friendly energy solutions, it is imperative to explore alternative materials and strategic approaches for TEGs that are both environmentally benign and abundantly available. To further enhance the thermoelectric utilization efficiency of composites, several hybrid energy harvesting units have been developed to capture thermal energy and convert it into electricity [238]. TENGs play a crucial role in thermal exchange and management systems between the human body and the environment. Consequently, the development of wearable electronic textiles for personal TM represents an effective approach to achieving individual thermal comfort and reducing building energy consumption [98]. Distributed low-grade waste thermal sources include solar thermal energy, residual thermal from electronic devices, and human body thermal. Significant challenges remain in extracting these valuable low-grade thermal energies using existing technologies, with constraints relating to material development costs, device maintenance expenses, system volume, and external temperature differentials [116]. Therefore, technologies capable of utilizing waste thermal energy through low-cost, flexible, and adaptable conversion methods are highly desirable. TENGs demonstrate broad application prospects in the field of distributed energy harvesting. Previous studies have provided detailed information on the current development status of the thermal energy harvesting in Table 2. Ou Yang et al. [239] designed an adaptive rotational TENG that integrates TEG with matching transformer circuit. The TENG facilitates friction reduction through the inherent elasticity of the dielectric layer, while the TEG enables the reuse of inevitable waste thermal via the Seebeck effect. Furthermore, the maximum output conversion efficiency of this TENG system reaches 96.4% when coupled with the specifically designed transformer circuit (Fig. 12a). The TENG is involved throughout the entire process of energy harvesting, conversion, and utilization. TENGs are characterized by diverse material selection, lightweight, low cost, simple structure, multiple operational modes, and other distinctive features. Wei et al. [240] proposed an energy conversion system for harvesting low-grade thermal energy based on the Curie effect and TENG technology. The system utilizes the phase transition phenomenon between ferromagnetic and paramagnetic states under alternating magnetic and thermal fields. The ferromagnetic material achieves autonomous reciprocating motion between cold and hot zones to drive TENG operation, thereby realizing the conversion from thermal energy to mechanical energy and finally to electrical energy. As the temperature at the bottom of the nickel block gradually increases, the magnetic moments within the heated region rotate due to internal thermal perturbations. The temperature recorded by the infrared thermal imaging equipment reached 436.0 °C (Fig. 12b). The nickel block continues its reciprocating oscillation until the temperature decreases below the Tc temperature. During continuous reciprocating motion, Qsc of 325 nC can be achieved. This TENG is widely used in various fields for thermal energy harvesting and conversion. This research represents the first instance of intelligently coupling the Curie effect in magnetic materials with the triboelectric effect in TENG devices, ingeniously harnessing the phase transition of ferromagnetic materials across thermal gradients to drive self-sustained reciprocating motion. The tri-level “thermal–mechanical-electrical” conversion mechanism constitutes huge breakthrough in energy harvesting systems. TM design must holistically consider the entire process of “thermal collection, thermal transfer, thermal conversion, and thermal utilization” [95]. Thermal energy harvesting technology transforms TM systems from mere energy-consuming units into potential energy-supplying units, making them an indispensable component within the system architecture. Thermal energy harvesting device serves as a built-in temperature sensor and energy source. It is the foundational first step in TM process [49]. By integrating the triboelectric layer with thermoelectric or PCMs, TENGs can simultaneously harvest both mechanical energy and ambient thermal energy. Material designers consider not only the triboelectric series but also holistically evaluate key parameters such as thermal conductivity, specific thermal capacity, phase *Tg*, and thermal stability. Such a comprehensive approach is essential for developing a new generation of highly efficient, robust, and intelligent TENG systems.

**Table 2:** Form, mode, output performance, and type of TM materials

Materials	Working mode	Form	Output voltage (V)	Output current	Power density/transferred charge	Type of thermal management	Refs
PANI/CNTs/PDA-Cotton fabric	TENG	Fabric	200	32 μA	2.5 W m^−2^	Thermal conversion	[126]
Gd-based Curie	TENG	Film	220	3.07 μA	806 nC	Thermal harvesting	[241]
BaTiO_3_@PVDF-HFP nanofibers	TENG	Film	482.5	42.2 μA	126.2 nC	Thermal activation	[242]
TOCNF/BNNS/MXene	TENG	Film	79.6	7.6 μA	30.7 nC	Thermal conduction	[26]
Silk fibroin/TiO_2_	TENG	Film	40	–	–	Thermal comfort/Radiative cooling	[243]
Multifunction porous	TENG	Film	89.4	0.88 μA	122.68 W m^−2^	Thermal insulation	[244]
B_4_C/PVDF	TENG	Film	155.4	7.9 μA	0.33 W m^−2^	Thermal conduction	[161]
WKF/PDMS/h-PVDF	TENG	Fabric	301.3	45.5 μA	1.37 mW m^−2^	Thermal resistance	[245]
PVDF/GO	TENG	Foam	550	11 μA	2.78 W m^−2^	Thermal conduction	[113]
Pulsating heat pipe/PTFE	TENG	3D generator	14	200 nA	1.05 μW m^−2^	Thermal-electric conversion	[246]
Ba(Cu_0.5_W_0.5_)O_3_-based ceramic Powder/PMDS	TENG	Film	127	3.16 μA	5.3 μW m^−2^	Thermal resistance	[247]
Windproof textile andtextile lining	TENG	Textile	66.98	1.81 μA	23.35 nC	Thermal insulation	[11]
Natural rubber/CNT/buckypaper	TENG	Paper	27	1.8 µA	15 nC	Thermal conduction	[179]
WKF/Ag@Mo_x_Fe_1-x_Se/MXene/PDMS	TENG	Film	220	23 µA	1.5 mW m^−2^	Thermal conversion	[248]
PDMS-BaTiO_3_ and PET	TENG-PENG	Film	40	2 µA	44.4 mW m^−2^	Thermal engine	[106]
Polyamide acid/PI/nanofiber	TENG	Film	185	36 µA	60 nC	Thermal driven	[249]
Aluminum nitride/PI@AIN	TENG	Film	502	–	–	Thermal dissipation	[250]
TPU/DMF	TENG	Film	740	–	–	Thermoelectric generator	[251]

### Thermal Energy Conversion

In contemporary society, global energy demand continues to rise at an annual rate of 2.3%. Substantial amounts of thermal energy (at temperatures below 200 °C) are being wasted due to the lack of efficient collection and conversion technologies [252]. The “invisible energy” in the environment is widely present in various scenarios, including waste thermal emissions from industrial processes, automotive exhaust, the infrared spectrum of solar radiation, geothermal gradient resources, and ambient thermal fields formed by diurnal temperature variations [60]. Following thermal energy collection, the phenomenon of converting thermal energy into other forms of energy naturally occurs. The thermoelectric conversion phenomenon refers to the direct transformation of a temperature gradient at the interface of two heterogeneous materials into electrical energy. It is a physical process that currently represents the most common form of thermal energy conversion [253]. As solid-state energy conversion systems, thermocouples enable the direct conversion of temperature differences into electricity through the Seebeck effect [254]. However, the energy conversion efficiency inherently faces an upper limit. It was constrained by the limitations of existing thermoelectric conversion technologies and the irreversible thermodynamic processes involved [255]. Efficiently converting such distributed and intermittent thermal energy into electricity remains a significant challenge in the field of energy utilization. The Joule heating conversion process in TENGs shares the same fundamental principle as in conventional high-power electrical devices but operates under distinct conditions. TENGs are inherently characterized by high voltage, low current, and high internal impedance. Current TENG designs often exhibit low current output and very large internal impedance, which includes the bulk resistance and surface resistance of the triboelectric layers, as well as the contact resistance at the friction interface. As charges transfer across surfaces or through interfaces, significant localized Joule thermal can be generated at high-resistance points within the composites [202]. It represents unique and critical thermal generation mechanism in TENGs, distinguishing them from other types of generators. Although generally limited, the thermal contribution becomes non-negligible when relatively high-resistance electrodes are used. Joule heating in TENGs primarily occurs at three locations: (a) the triboelectric contact interface, (b) within high-impedance triboelectric layers, and (c) at the junctions between electrodes or wiring. The generated thermal may activate deep charge traps in polymers, leading to accelerated release of stored triboelectric charges [75]. It reduces the surface charge density, which is a key factor contributing to the degradation of triboelectric output performance. To address these issues, mitigation strategies can be categorized into three approaches: reducing heat generation at the source, enhancing heat dissipation through structural design, and integrating TM materials such as PCMs. First, reducing thermal generation involves lowering the resistance of both triboelectric layers and electrodes. Enhancing thermal dissipation focuses on incorporating thermal conduction pathways and thermal dissipation structures. Both strategies are often implemented by doping polymers with highly conductive fillers such as CNTs or GO to fabricate triboelectric composites. It improves the thermal resistance and thermal stability of the composites, essentially enabling the material to withstand the thermal generated during normal operation. Many studies select polymers with high glass transition temperatures (*Tg*) and high thermal decomposition temperatures. Alternatively, developing high-temperature-resistant triboelectric materials based on ceramics or silicon carbide is another viable direction. Optimizing the interface between triboelectric layers and electrodes can also reduce both contact resistance and interfacial thermal resistance. In contrast, the integration of PCMs into TENG Joule TM systems represents a promising future direction for active monitoring and regulation [179]. By leveraging the thermal absorption characteristics of phase transitions, temperature-controlled adaptive circuits can be designed to adjust the load or operating frequency upon detecting abnormal output (caused by overheating). In summary, in addition to frictional losses during TENG operation, electrical losses (Joule heating) warrant equal attention.

The energy dissipation mechanism severely restricts the practical output performance of thermoelectric conversion systems. Breakthroughs in energy utilization can be achieved through the development of interfacial engineering and thermoelectric materials. To this end, global research teams are conducting in-depth studies across multiple technical pathways, including the development of new thermoelectric materials, enhancement of the pyroelectric effect, and optimization of thermomagnetic generators. Cátia Rodrigues et al. [238] proposed an energy production technology capable of converting low-grade thermal energy into electricity. By synergistically coupling the triboelectric and thermomagnetic effects, they have developed a device capable of generating power under temperature gradients approaching ambient conditions. The thermomagnetic effect enables the excitation of ferromagnetic materials to perform periodic and sustained motion under temperature differentials below 30 °C. The methodology presents a feasible thermal energy conversion strategy. It significantly expands the applicability of triboelectric systems in the domain of near-room-temperature thermal harvesting. Zeng et al. [256] fabricated the TENG termed thermally driven TENG (TD-TENG). It utilized a metallic beam with bistable dynamic characteristics to induce sustained mechanical oscillations, subsequently converting the kinematic energy into electrical output. This research systematically elucidated the internal thermal processes of the device. The TD-TENG demonstrates a power density of 323.9 mW m^−2^ at 59.5 °C, representing the highest recorded value among documented TENG-based thermal energy harvesters. The operational temperature range of the TD-TENG can be modulated by adjusting bimetallic parameters, thereby enabling adaptable and versatile functionality across a broad spectrum of thermal gradients. Figure 12c illustrates a cross-sectional view of the TD-TENG, wherein the primary thermal transfer mechanisms encompass thermal convection, conduction, and radiation. During thermal transmission, thermal resistance impedes the flow of thermal through components. It can be analogous to electrical resistance in charge transport, with temperature being conceptualized as an electrical potential. The TD-TENG demonstrates a simple and cost-effective approach to thermal energy conversion and harvesting. Given the abundance of thermal energy in natural environments, the development of wearable energy harvesters based on thermal conversion processes has attracted considerable attention [257]. Such systems could harness and convert metabolic thermal generated by the human body to supply direct current for electronic devices. Zhang et al. [258] made poly(3,4-ethylenedioxythiophene):poly(styrene sulfonate) thermoelectric yarns via a wet-spinning approach and integrated CNT/cotton fabric absorption components with PVDF films. The innovative wearable hybrid TEG was achieved with thermal adhesive tapes, and thermoelectric filaments for solar energy harvesting. Under solar irradiation of 1500 W m^−2^, the TEG charged two commercial capacitors to Voc of 3.7 V within 800 s, achieving a volumetric energy density of 67.8 μJ cm^−3^ for both devices. The result demonstrates the system’s effective capability to convert solar energy into usable thermal and electrical power (Fig. 12d).

**Fig. 13:**
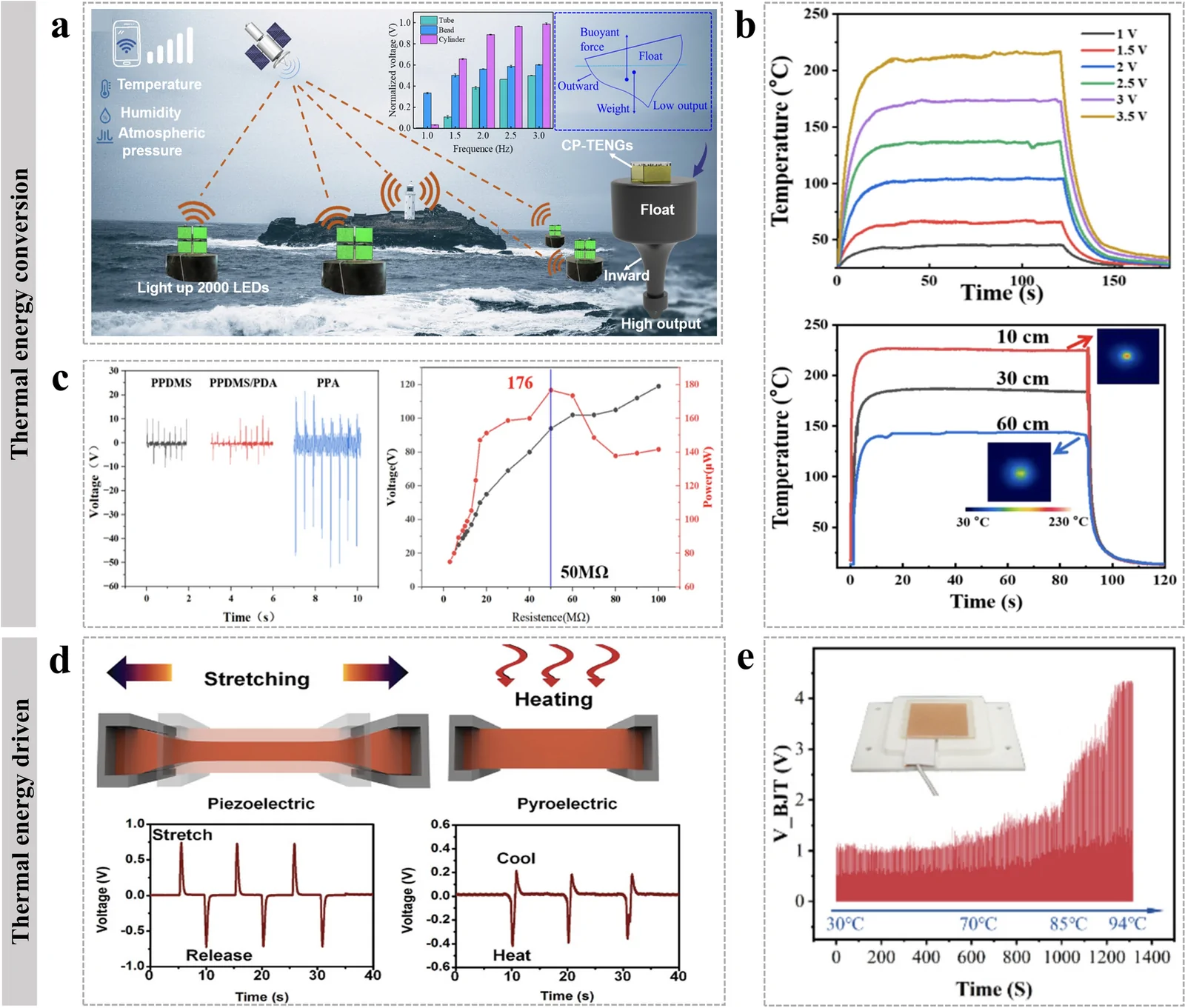
Thermal energy conversion and driven of the TENGs. **a** Schematic diagram of the wireless sea state monitoring system [262]. Copyright 2025, Elsevier Ltd. **b** Electric heating performance of the composite textile at different driving voltages (up). Temperature evolution curves of the composite textile with increasing heating time at different irradiated distances to NIR light (down) [265]. Copyright 2022, Elsevier Ltd. **c** Output performance of PPDMS, PDMS/PDA, and PPA films (left). Output voltage and power under different load resistance (right) [266]. Copyright 2025, Elsevier Ltd. **d** Piezoelectric output voltage from the HSNG under stretch-release condition, and pyroelectric output voltage under a thermal gradient [275]. Copyright 2014, Wiley–VCH. **e** Real-time ambient temperature monitoring using the S-TENG based on SMPs = 40 [276]. Copyright 2025, Wiley–VCH

The TENG achieves autonomous energy harvesting by converting ambient mechanical energy into electrical power. It has attracted significant research interest due to its distinctive advantages, including effective low-frequency mechanical energy harvesting, broad material compatibility, straightforward fabrication processes, and low manufacturing costs. In practical applications, TENGs can operate through various interfacial contact modes, including solid–solid, solid–liquid, liquid–liquid, and gas–liquid interactions [260]. These configurations enable surface electron transfer, thereby generating electricity. The solid–solid contact mode exhibits particularly high energy conversion efficiency. However, maintaining the necessary conditions for solid–solid triboelectrification requires continuous and rapid relative motion between the two contact layers while they remain in close physical contact. The operational paradigm imposes stringent demands on the robustness and durability of the contacting surfaces. In contrast, solid–liquid-based TENGs benefit from significantly reduced surface wear due to the lubricating effect of the liquid phase. It can operate for extended periods while maintaining favorable electrical output characteristics. Current research primarily focuses on harnessing the mechanical energy captured from ocean tides [93] and falling raindrops [261]. Liu et al. [259] developed a monolithic hybrid device through rational integration of high-performance semi-transparent polymer solar cells and liquid–solid TENGs. The TENG exhibits both high visible-light transparency and efficient thermal stability. Under 1 sun illumination, the hybrid device achieves a remarkable thermal conversion efficiency of 10.1%. It demonstrated outstanding photoelectric conversion performance with a maximum electrical power output of 101 W m^−2^ under clear weather conditions (Fig. 12e). Furthermore, the hybrid device can generate electrical energy through water droplet energy conversion, delivering a maximum power output of 2.62 W m^−2^. It enabled complementary green electricity production even during rainy conditions. This research can be used to effectively harvest and convert energy from waves, tides, and other water movements. Su et al. [262] proposed a novel cylindrical particle-based triboelectric nanogenerator (CP-TENG). The results demonstrated that the CP-TENG could power 2,000 serially connected LEDs (Fig. 13a). The system exhibited stable triboelectric output performance (~ 425 nC) within a temperature range of 25–65 °C. The CP-TENG shows significant potential for enabling remote wireless communication without the need for batteries.

Distributed environmental energy harvesting systems can integrate multiple energy conversion mechanisms, including thermoelectric and biomechanical energy conversion [263]. It utilized available energy sources within the deployment environment to achieve truly self-sustaining operation. However, individual micro-generators often suffer from functional limitations and low efficiency [264]. Current research predominantly focuses on integrating piezoelectric nanogenerators with TEGs. The selection of materials with excellent electrical and thermal conductivity presents a viable pathway toward enhanced energy conversion. Silver nanowires (AgNWs) have been extensively employed in thermal conversion studies. Liu et al. [265] presented a multifunctional flexible polymer-based wearable heater engineered by constructing a 3D AgNWs@MXene conductive network on fiber surfaces. It achieved efficient electrothermal and photothermal conversion performance through a straightforward alternating solution dip-coating technique. The AgNWs@MXene-modified polymeric textile heater attains a temperature of 215 °C under low voltage (3.5 V) operation and rapidly reaches 224 °C within 10 s under near-infrared laser irradiation (100 mW) (Fig. 13b). The AgNWs@MXene-TENG as the electrode, it can harvest energy from minute human movements and convert electrical signals into Voc (~ 62 V). Guan et al. [266] proposed an inventive TENG that utilizes a 0.1-mm-thick nylon film as the positive triboelectric material. The porous PDMS/MnCO_3_ composite modified with PDA and silver nanowires as the negative triboelectric material. The optimized TENG based on the PDMS/PDA/AgNWs (PPA) configuration achieves Voc of ~ 80 V, an effective output power density of 70.4 mW m^−2^. It maintains structural integrity over 10,000 contact-separation cycles without failure. At an internal resistance of approximately 50 MΩ, the TENG delivers a maximum power output of 176 μW and power density of 70.4 mW m^−2^. It represents the highest conversion efficiency attained during testing (Fig. 13c). The PPA/nylon-based TENG demonstrates significant potential for harvesting, converting, and storing clean energy. This research demonstrates outstanding comprehensive performance, environmental adaptability and structural innovation in the field of low-frequency micro-energy harvesting. In summary, thermal energy conversion technologies can be distinctly categorized into two major classes: direct thermal conversion and indirect thermal energy harvesting. Direct thermal conversion implies that thermal energy is transformed without any intermediate form. The conventional TEG serves as typical example of the category, whose conversion efficiency is directly governed by the material’s thermoelectric figure of merit and the magnitude of the sustained temperature gradient, which made it most effective under static and stable thermal gradients. In contrast, indirect thermal conversion involves the initial transformation of thermal energy into an intermediate form, such as mechanical energy. The energy is subsequently converted into electricity through the triboelectricity and electrostatic induction mechanisms of TENG. The fundamental distinction between these two approaches lies in their energy conversion pathways and the physical inputs upon which they rely.

### Thermal Driven

Low-grade thermal energy below 100 °C, derived from decentralized sources such as ambient environments, industrial waste thermal, and solar radiation, constitutes an abundant resource [267]. In recent years, actuators operating in thermal environments have garnered significant attention due to their rapid response to external stimulus, lightweight nature, and compact dimensions [268]. Through mechanisms such as thermal expansion and hydrolysis, thin-film actuators are capable of harnessing or recovering thermal energy from the environment [269]. Thermally driven actuation refers to the utilization of useful work or energy derived from thermal conversion processes to power devices, systems, or operational procedures. Unlike thermal energy conversion, the core focus of thermally driven actuation research lies in the end-use application of energy and the operational dynamics of systems. To date, most devices primarily rely on materials such as polymers, hydrogels, and composites. These systems exhibit constrained unidirectional and anisotropic motions, namely expansion—contraction and stationary—bending behaviors [270–272]. Currently, the major of research on TENG technology is confined to the harvesting and conversion of mechanical energy from the environment, thereby limiting its application scope. When stimulated by external thermal sources, thermoacoustic generators produce acoustic energy, thereby providing TENGs with a stable and sustained mechanical energy supply. By coupling the thermoacoustic engine with an acoustic-electric generator, the acoustic energy generated by the thermoacoustic engine can be converted into electrical energy. Zhu et al. [273] proposed an ingenious thermoacoustic-electric energy conversion pathway termed “thermoacoustically driven TENG”. The CS-TENG is coupled to the end of the resonance tube in a standing-wave thermoacoustic engine. In feasibility experiments investigating the application of TENG as an acoustic-to-electric conversion device within thermoacoustic power generation systems, the thermoacoustically driven TENG achieved Voc output of up to 10 V. The maximum output power reached 0.008 microwatts when connected to an external load resistance of 400 MΩ. In contrast, due to the high-frequency and small-amplitude vibration characteristics of piezoelectric transducers, thermoacoustic piezoelectric power generation technology. It is only suitable for microscale thermoacoustic power generation systems and exhibits relatively low acoustic-to-electric conversion efficiency [274]. Lee et al. [275] fabricated a fully stretchable and flexible hybrid piezoelectric–thermoelectric micro-generator (HSNG) and verified its stretchability along with the stability of piezoelectric–thermoelectric output after deformation. Figure 13d illustrates the piezoelectric Voc of the HSNG under stretching and release conditions, as well as the thermoelectric output under thermal gradients near the device during heating and cooling. When thermal was applied to the GO side of the HSNG, superior thermoelectric output performance was achieved. In contrast, only a minimal Voc was observed when the same temperature gradient was applied to the PDMS-CNT side of the device. The study demonstrates the feasibility of the hybrid micro-generator and its potential to contribute to advancements in areas such as wireless sensing and energy harvesting. However, research on the integration of TEG with TENG remains limited to date. Exploring novel thermal conversion approaches characterized by high reliability, low cost, simple structure, and high efficiency has emerged as a crucial direction for the development of thermally driven power generation technologies. Li et al. [276] developed a thermally adaptive TENG based on shape memory polymers. The electrical output of the SMP-based TENG exhibits a significant positive correlation with the thermal level of the composite. When the temperature exceeds the glass *T*g, the output increases in an approximately linear manner. The composite demonstrates the potential of this TENG for applications in thermally triggered switching and self-powered temperature sensing. In thermal sensing experiments, a planar S-TENG fabricated with SMPs-40 was gradually heated from room temperature to 94 °C. Both the Isc and the corresponding signal showed a continuous increase with rising temperature, reaching their peak values at approximately 90 °C (Fig. 13e). This study confirms the exceptional thermal sensing capability of the system. The response speed and magnitude of thermally driven deformation remain limited at this stage. The approach equips us with an ingenious capacity for actively managing thermal energy systems, rather than merely collecting energy passively. Future thermally driven TENG systems will be capable of more intelligently interpreting environmental thermal signals and self-generated electrical signals, thereby enabling more complex decision-making and control functionalities. By utilizing thermally responsive materials compatible with human body temperature, intelligent wearable devices for health monitoring and rehabilitation therapies can be developed. However, the device still needs for further in-depth research on thermal response rate, integration level, and long-term stability [277]. The direction of research undoubtedly offers an imaginative solution for the future development of intelligent wearable thermal-responsive devices, environmental thermal sensing, and advanced electronics.

### Thermal Conduction

**Fig. 14:**
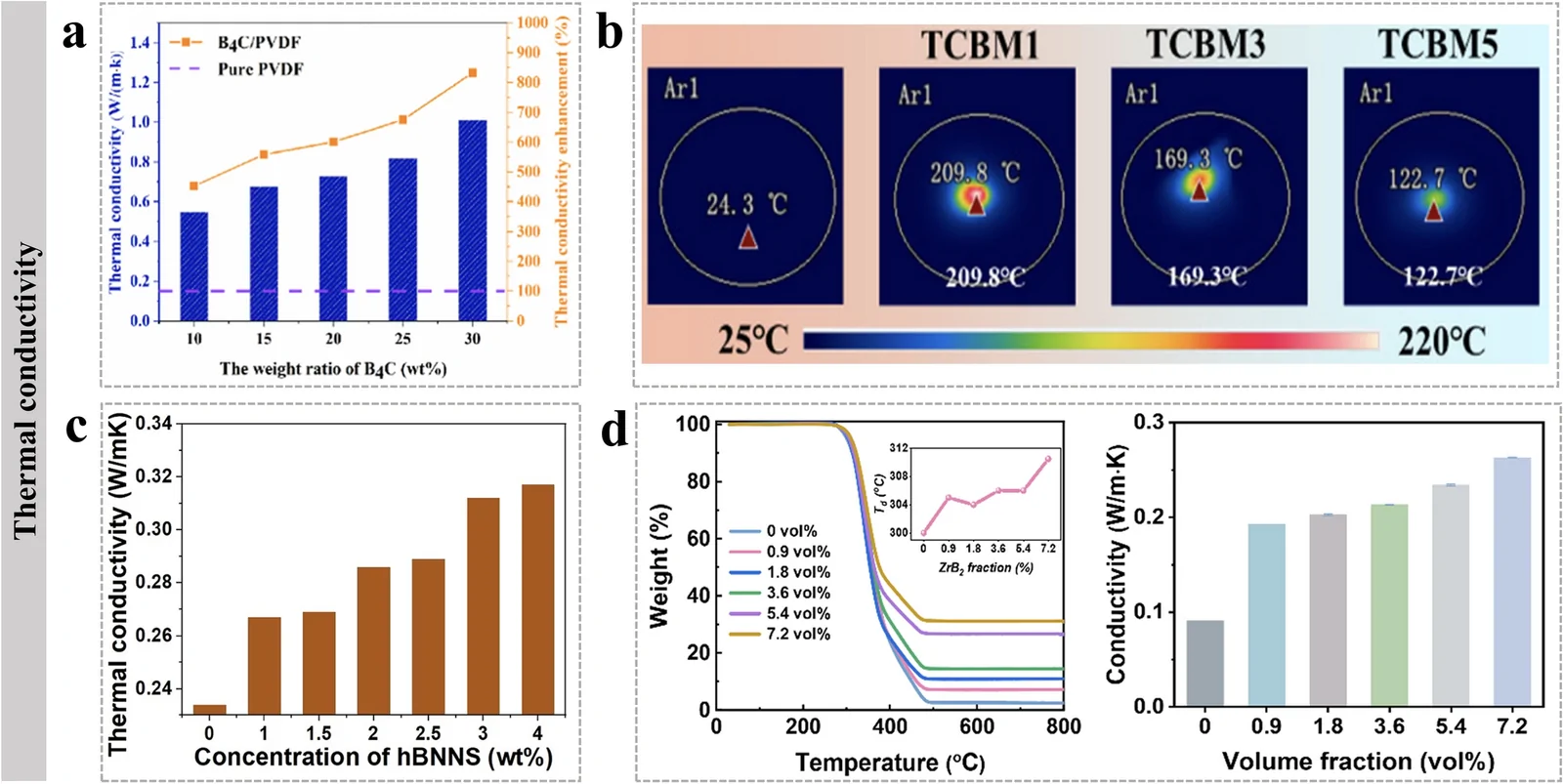
Thermal conduction of the TENGs. **a** Thermal conductivity of B_4_C/PVDF composites at room temperature [161]. Copyright 2024, Elsevier Ltd. **b** Infrared thermal image irradiated at a power density of 0.3 W cm^−2^ [26]. Copyright 2025, Elsevier Ltd. **c** Thermal conductivity of hBNNS/PVDF composites with different BNNS contents [285]. Copyright 2024, Elsevier Ltd. **d** TGA curves and the decomposition temperature (*T*_d_) of the PU/ZrB_2_ composites (left). Thermal conductivity of the PU/ZrB_2_ composites (right) [25]. Copyright 2024, Multidisciplinary Digital Publishing Institute

With the ongoing miniaturization and high integration of TENGs, the Joule heating effect generated by interfacial friction during operation has become increasingly pronounced. It leads to significant thermal accumulation within the device [161]. The phenomenon not only accelerates material aging but also compromises the output stability of the device in high-temperature environments. Consequently, the development of triboelectric composites with efficient thermal dissipation capabilities is of critical importance. To enhance wear resistance and thermal conductivity, various strategies have been proposed, such as incorporating lubricants into triboelectric materials, optimizing device structures, and employing doping techniques [278]. These approaches have effectively improved the durability and operational stability of TENG-based devices [279]. Currently, 2D materials represented by montmorillonite [280], hexagonal boron nitride (h-BN) [281], and black phosphorus [282] have garnered significant research attention. These materials typically exhibit mechanical flexibility, high specific surface area, and exceptional electrical/thermal conductivity as well as flame-retardant properties. It continuously promoted innovation in advanced materials and surface/interface chemistry. PVDF demonstrates outstanding chemical resistance, high-temperature tolerance, and favorable dielectric properties [283]. Owing to the electron-accepting tendency of fluorine atoms in its molecular chains during triboelectric processes, PVDF is commonly employed as a negative triboelectric material. Wang et al. [161] synthesized a B_4_C/PVDF composite, which exhibits thermal conductivity of 0.727 W m^−1^ K^−1^, representing 600% enhancement compared to pure PVDF film (Fig. 14a). Concurrently, the TENG demonstrates improved anti-corrosion properties and enhanced electrical output performance when integrated into the TENG device. It achieved Voc of 155.4 V, Isc of 7.9 μA, and maximum output power density of 0.33 W m^−2^. H-BN has been established as a robust solid lubricant, exhibiting remarkable lubricating properties [284]. Its 2D layered structure facilitates the formation of a protective lubrication film on contact surfaces during friction processes. Furthermore, h-BN nanosheets tend to curl into rod-like or spherical structures under tribological conditions. Lang et al. [26] fabricated the ternary composite comprising TOCNF, boron nitride nanosheets (BNNS), and MXene through a vacuum-assisted filtration process. The resulting composite film demonstrates exceptional thermal conductivity, reaching 16.72 W m^−1^ K^−1^. As the BNNS content increases, the thermal conductivity of the composite film progressively improves, accompanied by a significant reduction in temperature (Fig. 14b). It achieves Voc of 79.6 V, Isc of 7.6 μA, Qsc of 30.7 nC, and maximum power density of 272.5 mW m^−2^. By integrating the energy supply unit and sensing unit into a single system, an intelligent “sensing-power supply-actuation” integrated system has been constructed. The composite film-based TENG enables on-demand, real-time, and dynamic TM, significantly enhancing energy utilization efficiency and control precision. The synergistic effect of structural innovation and material optimization in this composite facilitates the transition of TENGs from laboratory prototypes to practical applications, effectively bridging the gap between theoretical potential and real-world utility. Zhao et al. [285] developed h-BN/PVDF composite negative triboelectric material with remarkable electrostatic performance and high thermal conductivity. The composite film exhibits 22.2% enhancement in thermal conductivity (Fig. 14c). The optimized TENG achieves peak Voc of 434 V, Isc of 53 μA, and power output of 4.84 mW. It could power an intelligent hygrometer and can directly illuminate 102 serially connected white LEDs. Wang et al. [25] incorporated zirconium diboride (ZrB_2_) into the TPU matrix to fabricate the composite. TGA revealed that the initial decomposition temperature (*T*d, at 95% mass retention) increased from 300 to 310 °C with the addition of ZrB_2_, indicating a significant enhancement in thermal stability (Fig. 14d, left). Concurrently, the composite achieved a high thermal conductivity of 0.26 W m^−1^ K^−1^, which was leveraged to develop a high-performance TENG (Fig. 14d, right). The resultant TENG exhibited outstanding electrical output, characterized by Voc of ~ 347.6 V, Isc of ~ 3.61 µA, and Qsc of ~ 142.4 nC. Furthermore, the device demonstrated excellent sensitivity. The current research constitutes a significant contribution toward advancing multifunctional TENGs for practical applications.

The persistent issue of degraded triboelectric output performance due to the accumulation of frictional thermal within TENGs remains a challenge. Designing porous structures in composites to maximize thermal dissipation area may compromise mechanical strength and reduce effective contact area, adversely affecting triboelectric charge generation. Similarly, achieving an optimal balance between the thermal conductivity of composites and the output performance of TENGs is an ongoing concern. The thermal resistance at the interface between the filler and the matrix, as well as at the interface between the device and the thermal sink. It is a critical factor affecting overall thermal conduction efficiency. In the future, TM is expected to significantly enhance the operational stability, durability, and environmental adaptability of TENGs, while it also expands their multifunctional applications. However, the challenge of integrating highly thermally conductive materials with TENGs requires further exploration. It is important to find the optimal balance between the loading level of high-thermal-conductivity fillers and the flexibility, mechanical strength, and cost of the TENG.

### Thermal Energy Storage

**Fig. 15:**
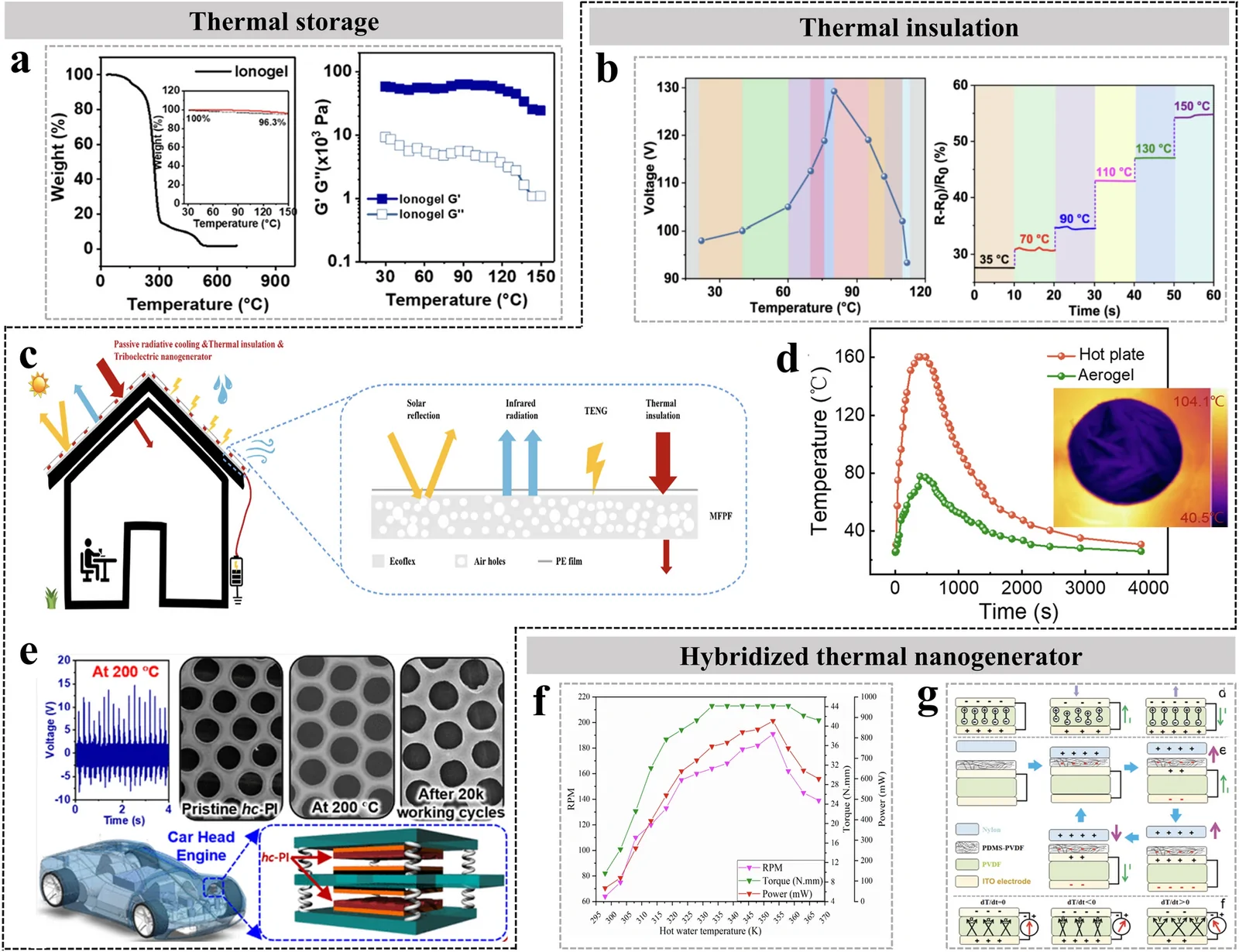
Thermal energy storage, insulation, and hybridized thermal nanogenerators. **a** TG curve of ion gel (left). *G*′ and *G*′′ of ion gel as a function of the temperature (right) [289]. Copyright 2021, Elsevier Ltd. **b** Fitting curve of Voc of FR-TENG at different temperatures (left). The resistance response of FR-TENG at different temperatures (right) [292]. Copyright 2022, Elsevier Ltd. **c** Application schematic illustration of the MFPF as a thermal-insulating radiative cooler and TENG and the mechanism diagram of the MFPF [244]. Copyright 2024, Elsevier Ltd. **d** Temperature–time curves of cellulose/CNT at 160 °C (Thermal infrared images of cellulose/CNT aerogel at 100 °C) [121]. Copyright 2025, Elsevier Ltd. **e** Schematic illustration of TENG with excellent thermal stability and enhanced electrification performance [64]. Copyright 2022, American Chemical Society. **f** Change in key performance parameters and torque with varying hot water temperature [295]. Copyright 2022, Multidisciplinary Digital Publishing Institute. **g** Working mechanisms of the PENG, TENG, and the PyENG [298]. Copyright 2015, Wiley–VCH

The relentless growth in global energy consumption has precipitated severe energy crises and exacerbated climate change challenges [286]. In response to these pressing issues, a lot of nations are increasingly shifting their focus toward renewable energy sources. These sources hold significant potential to power the world in a low-carbon and sustainable manner, such as wind, hydro, and solar power [287]. However, the inherent intermittency and volatility of renewable energy significantly impede their broader application. Given that approximately 80% of global energy consumption is thermal-related, TES has emerged as a prominent research focus to reconcile the mismatch between the fluctuating nature of energy supply and the continuous demand [288]. The capture and storage of decentralized ambient thermal energy, along with waste thermal generated by TENGs’ own friction, enable the spatiotemporal transfer and efficient utilization of energy by releasing it when needed (e.g., during windless or nighttime conditions). TES is a technology that utilizes PCMs to accumulate surplus thermal and release it at the required time or location for heating [213]. This approach effectively addresses spatiotemporal mismatches in thermal supply and demand, thereby enhancing the efficiency, stability, and reliability of thermal energy utilization. Dang et al. [289] synthesized a creative melt-processable hydroxypropyl cellulose composite. It presented a promising approach for fully melt-processable fabrication. The developed ion gel exhibits a unique combination of properties including electrical conductivity, self-healing capability, UV resistance, thermal stability, and complete recyclability. A multifunctional TENG was fabricated employing this ion gel as a functional electrode, demonstrating triboelectric output characteristics of 80 V, 2 µA, and 27 nC, respectively. The maximum power density achieved was 67.9 mW m^−2^ at a constant frequency of 3 Hz. Thermal stability tests revealed negligible weight loss of the ion gel when maintained at 150 °C in air atmosphere (Fig. 15a, left). Furthermore, rheological measurements showed that the storage modulus (*G′*) consistently remained superior to the loss modulus (*G*″), confirming the material’s elastic solid behavior (Fig. 15a, right). Polymer chains facilitate the formation of a more densely cross-linked network within the ion gel. Although TENGs can harvest ambient thermal energy, their electrical output is often intermittent and unstable. TES systems address this by storing thermal energy during periods of abundance and releasing it during deficits [290]. The mechanism ensures that the thermal source driving the TENG remains relatively stable and persistent, thereby enabling a more continuous electrical output. Materials such as PCMs maintain nearly constant temperature during the thermal storage and release cycles [291]. The integration of TENGs with such materials paves the way for the development of TM materials capable of generating electricity. The integration of TES systems ensures the continuous operation of TENGs even during intermittent thermal source availability or under significant environmental temperature fluctuations. This capability substantially enhances their applicability in harsh or remote environments, directly addressing a fundamental bottleneck in harvesting ambient thermal energy with TENG technology.

### Thermal Insulation

Flexible thermal insulation materials hold significant application value in building energy efficiency and extreme TM due to their deformable adaptability and efficient thermal protection properties [121]. However, they still face technical challenges such as insufficiently regulated thermal diffusivity and limited high-temperature stability. It is primarily because energy dissipation mechanisms severely constrain the practical output performance of thermoelectric conversion systems. To play a pivotal role in the pursuit of sustainable development, the performance of thermoelectric TENGs must be substantially enhanced. Therefore, materials characterized by high Seebeck coefficients, high electrical conductivity, and low thermal conductivity are frequently employed. The low thermal conductivity signifies exceptional thermal insulation properties [155]. Most studies demonstrate thermal insulation performance primarily through the measurement of thermal conductivity. In high-temperature environments, friction layer materials may undergo structural collapse, softening, degradation, or even combustion. These phenomena can lead to a sharp decline in output performance, ultimately resulting in functional failure of the device. Consequently, maintaining device stability and sustaining outstanding triboelectric output performance under high-temperature conditions have emerged as a critical research direction. Based on this foundation, high-performance thermal insulation materials (such as aerogels or BNNS) can be incorporated [26]. The insulating materials can construct a thermal protection barrier for TENGs, effectively blocking external high temperatures and ensuring their stable operation in demanding applications including thermal power generation and aerospace. Wang et al. [292] fabricated a ternary thermal insulation composite film comprising black phosphorus/phytic acid/CNF via solution blending. Compared to pure CNF film, the composite exhibits a 64.6% reduction in the thermal release rate and a 47.6% decrease in the total thermal released. The LOI of the composite film is elevated to 39.1%. A SE-TENG constructed based on this composite film not only exhibits biodegradability but also functions as a rapid-response thermal sensor for early fire warning. Furthermore, the thermal insulation TENG maintains outstanding triboelectric output performance across varied thermal environments (Fig. 15b). Wu et al. [244] developed a scalable porous film designed for thermal insulation, passive radiative cooling, and triboelectric power generation via straightforward emulsion polymerization method (Fig. 15c). The multifunctional porous film (MFPF) contains a substantial number of micropores and exhibits an ultralow thermal conductivity of 0.05 W m^−1^ K^−1^. Under direct sunlight, the composite film achieves a measurable temperature reduction of approximately 4.5 °C. The MFPF-based TENG reaches a maximum power density of 0.03 W m^−2^, a triboelectric effect primarily attributed to the strong electronegativity of the Ecoflex component. When the temperature of the electrode layer becomes excessively high, it can trigger the thermionic emission effect, causing charges to leak through thermal emission rather than following the intended circuit path. The phenomenon significantly degrades the TENG’s output performance and charge retention capability. An effective thermal insulation layer can protect the electrodes, maintaining them at a lower temperature to ensure efficient charge output. In wearable device applications, the thermal insulation layer serves to prevent skin irritation caused by thermal loss, thereby safeguarding human health [11, 244]. Furthermore, the TENG integrated with a thermally insulating electrode can function as an intelligent skin for robots. The sophisticated design enables simultaneous tactile and pressure perception through contact electrification, while providing critical protection for internal electronic components against extreme external temperatures.

The superior thermal insulation performance of foamed materials fundamentally stems from their meticulously engineered multiphase composite structure, which comprises coexisting solid and gaseous phases with highly refined porosity [194]. The introduction of foaming into thermoplastic matrices represents a transformative advancement with broad-ranging benefits. Foam and aerogel structures are renowned for their lightweight characteristics, exhibiting exceptional specific impact strength and enhanced toughness [178]. Within these foamed architectures, the solid phase is partitioned into numerous thin cell walls and struts, creating a discrete network characterized by “point contacts” or “surface contacts.” The unique configuration forces thermal flow to propagate along these tortuous and elongated solid pathways. It drastically reduced the effective cross-sectional area for thermal conduction [60]. Consequently, the solid-phase contribution to thermal conductivity becomes extremely low. These materials provide exceptionally high thermal resistance while maintaining ultralow density [61]. It implies that equivalent or superior insulation performance can be achieved with minimal material mass compared to dense alternatives, which is a critical advantage for weight-sensitive sectors such as aerospace and transportation. Furthermore, the foaming process introduces potential for multifunctional performance, including capabilities for energy harvesting and integrated TM applications [293]. Chen et al. [121] proposed a highly elastic, lightweight, and high-performance aerogel-based TENG. The cellulose/CNT/PVDF-trifluoroethylene (PVDF-TrFE) composite aerogel was prepared via a straightforward and effective freeze-drying strategy. The resulting AA-TENG demonstrates exceptional elasticity and outstanding thermal insulation properties (Fig. 15d). As the β-phase content in the PVDF-TrFE composite aerogel increases (reaching 88.95%), the triboelectric output performance is significantly enhanced by 57%. The use of thermally functional materials as the substrate or encapsulation layer for TENGs can impart integrated TM capabilities to the device without requiring additional TM modules. Unlike the protective effect of thermal insulation, the high-temperature resistance of composites refers to their ability to maintain physical, chemical, and mechanical properties without significant degradation or failure. The property focuses on the maximum temperature that the material itself can withstand. Bui et al. [64] proposed a triboelectric material that utilizes surface-patterned, high-temperature-resistant thermoplastics to mitigate typical limitations of TENGs. This customizable honeycomb-structured polyimide (PI) was fabricated via a simple method. The composite film shows excellent thermally stable and flexible thermoplastic properties of PI. It combined with the open-ended honeycomb pattern array enables the unique structure to effectively enhance the charge excitation efficiency of the honeycomb TENG (hc-TENG) (Fig. 15e). The hc-TENG achieves an output power density of 1.05 W m^−2^, which is 22 times higher than that of a conventional TENG assembled with flat PI. The composite can withstand temperatures as high as 200 °C without damage to its hexagonal honeycomb pore array. Besides, owing to the dimensional and structural stability, the hc-TENG maintains stable operation at the high-temperature environment.

Overall, research scholars widely concur that the key to broadening the operational temperature range of TENG materials lies in the chemical modification of tribological materials [276]. Enhancing high-temperature performance can be achieved either by incorporating thermally stable polymer molecules into the tribological materials or by modifying the surface structure of polymer-based friction materials to increase the contact area. Common thermal-resistant polymers are categorized into functional materials such as thermally conductive polymers, thermal insulation composites, and PCMs [64]. Among these, materials with excellent thermal conductivity include aluminum oxide, h-BN, aluminum nitride, and carbon-based materials. Carbon-based materials encompassed GO and carbon fibers [175]. Thermal insulation materials typically function by leveraging inherent porous structures or low thermal conductivity to impede thermal transfer. Material forms encompass aerogels, foams, and similar structures. Commonly utilized PCMs for TM include paraffins, FA, alcohols, hydrated salts, among others. The lower the thermal conductivity of a TM material, the greater its ability to impede thermal flow, the superior its thermal insulation performance [183]. Thus, the composites exhibit exceptionally low thermal conductivity, which contributes to both their insulation properties and high-temperature resistance. In practical high-end applications, where simultaneous excellence in thermal insulation and thermal resistance is often required, it can only be achieved through meticulous material design. The thermal resistance of a material with a specific thickness directly reflects the insulation effectiveness of that material layer under practical conditions [202]. Higher thickness and thermal resistance correspond to superior insulation performance. Materials with greater thickness can maintain their strength, stiffness, and elasticity at elevated temperatures, thereby sustaining stable CS-TENG or LS-TENG. Overall, in the field of TENGs, thermal insulation and high-temperature resistance are two complementary and indispensable key attributes [155]. The integration of insulating materials with TENGs is evolving from simple physical combination to a deep-level fusion of functional synergy and material-level innovation, aligning with the ongoing trend toward higher integration in electronic devices.

### Others

The efficient utilization of currently widely wasted low-grade thermal energy, such as industrial waste thermal, body thermal, and environmental temperature gradients. It can provide key technological support for building a sustainable society. The process of converting thermal energy into mechanical and electrical energy involves more than single TM system. The collection, utilization, management, and storage of thermal energy are often integrated within a unified system. In the operation of TENGs, their relevance to thermal energy is particularly strong. Therefore, designing TENG materials with efficient TM capabilities is imperative. Beyond the before mentioned TM-TENG applications, several niche or emerging use cases warrant a brief overview to fully capture the technology’s potential.

Thermomagnetic generators inherently produce motion (e.g., magnetic attraction and repulsion) under thermal driven [238]. It can be directly utilized to power TENGs, thereby establishing a “thermal-magnetic-mechanical–electrical” conversion chain. When a material is heated beyond its Curie temperature, it transitions from a ferromagnetic to a paramagnetic state. Within an applied magnetic field, it induced force variations (or abrupt changes in magnetic flux). The mechanism can ultimately drive the generator to produce an electrical current. Essentially, this process enables the direct conversion of thermal energy into mechanical or electrical energy, representing a specialized application within the broader domain of thermal energy conversion technologies [290]. Takahashi et al. [294] designed a disk-shaped rotor-based thermomagnetic generator. Driven by a thermal field, the device utilizes the magnetic phase transition of temperature-sensitive ferromagnetic materials and the dynamic magnetic flux formed by permanent magnets. Under load conditions, the TENG can generate sustained rotational output. The disk-shaped configuration enables a maximum output power of up to 6.0 W while maintaining the same volume of magnetic material (eddy current damping losses amount to merely 0.04 W). Kim et al. [295] developed a cylindrical thermomagnetic engine-based TENG. The device generates a maximum mechanical power of 1.1 W under a temperature difference of 45 °C between the hot and cold sources. As the thermal gradient increases, the force differential between gadolinium blocks adjacent to the permanent magnet is enhanced. The results in stronger magnetic attraction, thereby producing higher rotational speed and torque (Fig. 15f). Future micro-energy technologies will no longer rely on single device but will evolve into hybrid systems that synergistically integrate multiple effects to maximize energy harvesting from complex environments.

Among the various waste thermal recovery technologies, pyroelectric generators are garnering increasing attention due to their unique operational mechanism and structural advantages. The pyroelectric effect is exclusively sensitive to temperature fluctuations and remains unresponsive to stable thermal fields [296]. Notably, many pyroelectric materials also exhibit excellent triboelectric properties. Pyroelectric materials can be engineered into bilayer or multilayer architectures. Within such structures, a single material layer can generate a pyroelectric current in response to either temperature fluctuations or applied mechanical stress [21]. Pyroelectricity describes the generation of electric charge in polar materials via time-dependent temperature variations [16]. When a freestanding pyroelectric material approaches the 2D crystalline limit, it can produce charge through lattice polarization changes induced by temperature variations. The thermal-to-electrical energy conversion process can be effectively integrated with TENG [297]. The characteristic renders pyroelectric generators particularly well suited for thermal energy utilization scenarios involving intermittent thermal sources, low-temperature differentials, and low thermal flux densities. The application domains encompass environments with fluctuating temperatures, waste thermal recovery, and the harvesting of thermal energy from temperature variations on human or device surfaces. Wang et al. [298] proposed a hybrid nanogenerator with a multilayered architecture of PVDF nanowire-PDMS composite film/indium tin oxide electrode/PVDF composite film electrode. This device could harvest mechanical and thermal energy individually or simultaneously by leveraging the synergistic interplay of piezoelectric, triboelectric, and pyroelectric effects. Figure 15g displays the working mechanism of the hybridized nanogenerator. Furthermore, pyroelectric devices typically feature simple structures, controllable dimensions, and ease of integration, endowing them with significant potential for miniaturization and multi-field coupling design [296]. Owing to these attributes, pyroelectric generators demonstrate promising application prospects in fields such as flexible electronics, self-powered sensors, and intelligent materials. The hybrid nanogenerator, capable of simultaneously capturing mechanical and thermal energy, enables maximal utilization of ambient environmental energy. Thereby, it significantly enhanced the operational endurance and reliability of the system. The integration of pyroelectric generators with TENGs represents an inevitable trend in the evolution of micro-nanoenergy technologies [74]. Its fundamental significance lies in the synergistic exploitation of multi-physical fields and effects to achieve maximized harvesting and utilization of scattered ambient energy. The development prospect of this approach is to propel next-generation self-powered systems, intelligent sensing, and IoT nodes toward greater efficiency, miniaturization, and intelligence.

## Conclusion, Challenges, and Future Perspectives

**Fig. 16:**
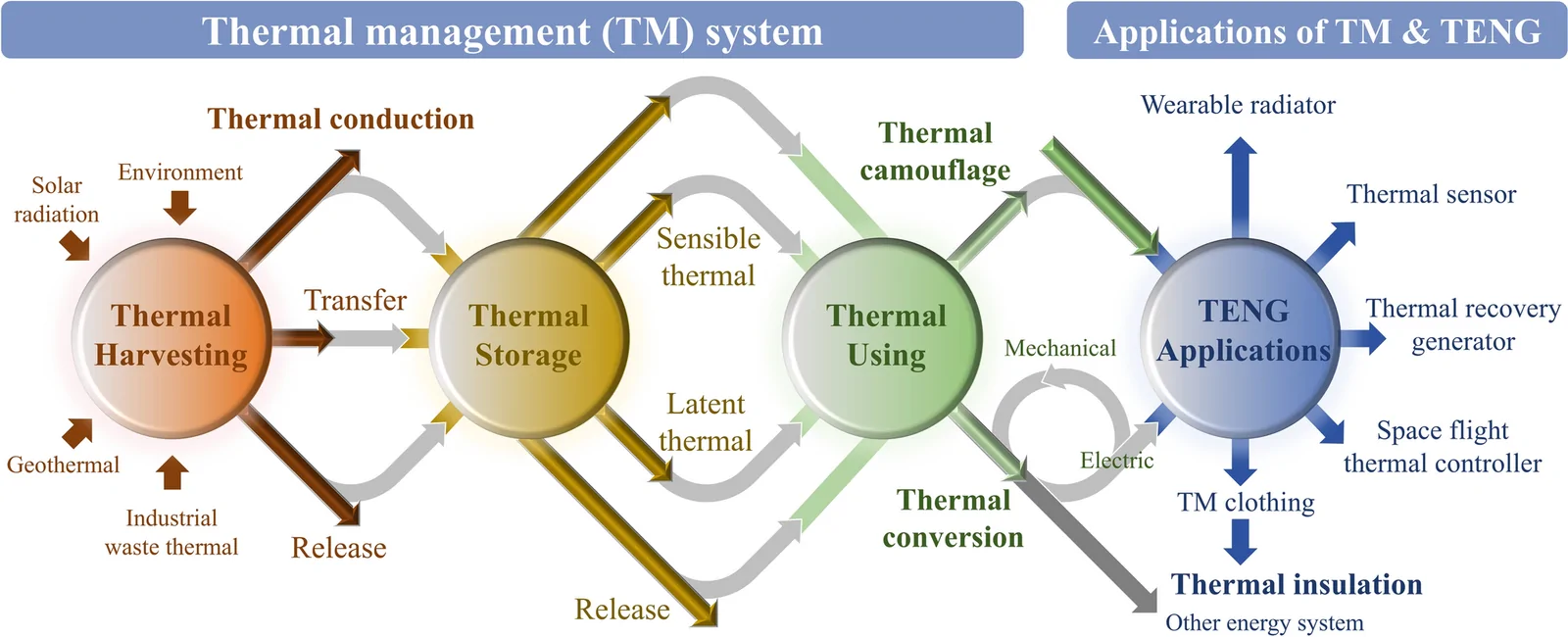
Connection between the TM system and TENG and its application

In summary, this review provides a concise introduction to the TM and representative directions of TENG, along with their applications across various fields. We have placed significant emphasis on the importance of TM materials in energy harvesting. The performance of TM materials not only broadens the application fields of TENGs but also enriches the material library available for TENGs. The review systematically elucidates the diverse roles played by prominent TM materials in enhancing the performance of TENGs, such as GO, MXene, CNT, MOFs, and PCMs. TM-based TENGs are extensively applied in areas including thermal energy harvesting, thermal energy conversion, TES, thermally driven systems, and thermally conductive materials (Fig. 16). Each application is discussed in detail, providing insights into the benefits and challenges of using TM-TENG. Despite the progress made in demonstrating the various applications of TM-TENG, there is still a vast opportunity for their continued advancement in the coming years. However, several challenges need to be addressed before they can be widely adopted. The challenges include the long-term stability of TM-TENGs, low output powers, manufacturing issues, material degradation over time, efficiency in varying environmental conditions, need for advanced materials and designs of TENG, and integration with existing energy technologies.

1. *Simplifying the preparation process of TM-TENG* Integrating TENGs with TM modules in a highly efficient and reliable manner involves significant complexity in terms of structure and manufacturing processes. To enable large-scale production of such energy management materials, it is essential to avoid laboratory-scale preparation methods, such as freeze-drying and vacuum filtration. Instead, processes like chemical vapor deposition can be employed to promote uniform dispersion of nanomaterials. Current research on the nanostructures of these TM-TENG materials remains limited. Nanowires, nanorods, and nanoparticles could offer larger surface areas, improve charge transport properties, and enhance triboelectric interactions. It can boost the overall performance of the devices. The continued advancement in this field requires a clear theoretical foundation for TENGs from both macroscopic and microscopic perspectives to guide the optimization of electrode and triboelectric layer materials. TM-TENG electrode materials hold potential for applications in areas such as wearable devices and self-powered medical equipment.
2. *Exploring other TM-TENG materials* The growing diversity of complex applications presents increasingly stringent demands for the performance of triboelectric materials. The polarity of functional groups significantly influences the output performance of these materials. Through modification and composite formation, the resulting structural configurations of triboelectric layers or electrode materials are often highly complex. The featuring surfaces have diverse functional groups. Under such conditions, the coexistence of functional groups with varying triboelectric polarities can impact overall triboelectric performance. Nevertheless, the presence of these functional groups renders biopolymers amenable to chemical modification. In terms of material selection, PCMs are widely utilized for their ability to sense, store, and transfer thermal energy. Chemically stable materials such as PW and FA, when encapsulated within porous polymer matrices, effectively address leakage issues while enhancing the mechanical strength of the composite. The high production costs of highly thermally conductive materials such as GO and MXene constrain their application in developing low-cost multi-mode TENG. While large-scale production is feasible for academic purposes through established nanomanufacturing techniques, further research is required to tailor these materials to specific target applications. Greater attention should also be directed toward the doping and functional of GO produced via laboratory-scale methods, which would facilitate its integration into broader TM-TENG composites to achieve multifunctionality. Leveraging their state transitions during phase change, these materials can alter the contact conditions and triboelectric output performance of TENGs, also serving as deformable triboelectric layers. It should develop triboelectric layer materials with enhanced wear resistance, reduced humidity sensitivity, and superior thermal/electrical properties. Future research on the integration of thermal insulation materials with TENGs is likely to focus more on developing intelligent material systems capable of dynamic responses (e.g., thermal-triggered switching) and self-healing functionalities. It could simultaneously drive down costs to facilitate the large-scale practical application of these multifunctional integrated devices. Furthermore, thermally responsive materials can undergo deformations that directly serve as mechanical energy sources to drive TENG operation when stimulated by thermal sources. It included thermally induced shape memory polymers and thermo-responsive hydrogels. A primary advantage of biopolymers lies in their exceptional biocompatibility, non-toxicity, and biodegradability. In the field of wearable TENG applications, biomass-based hydrogel thermal stimulus materials are frequently employed as substrates, fulfilling functions akin to artificial skin. Looking ahead, the future development trajectory points toward the rapid screening of optimal solutions from vast material combinations, thereby enabling the design of ideal materials that integrate high thermal conductivity, robust mechanical properties, and reliable stability.
3. *Improving the output power of TM-TENG* TENGs generally exhibit a relatively low output power density, making them unsuitable for directly driving high-power TM devices. Operating on the principles of contact electrification and electrostatic induction, TENGs possess inherently high internal impedance. The fundamental characteristic results in an output profile marked by high Voc but extremely low Isc. Furthermore, several factors collectively constrain the ultimate triboelectric power output, including mechanical energy dissipation during the friction process, incomplete charge transfer, surface charge dissipation, and impedance losses within the circuit. As a result of these limitations, the energy harvested by TENGs is typically sufficient only for powering microwatt to milliwatt-level sensors or micro-systems. The operational performance of TENGs is also highly susceptible to external environmental conditions, such as humidity, temperature, contact area, and impact distance. It is important to note that optimal testing conditions vary for different materials. Larger areas and higher impact frequencies do not universally guarantee better performance. Researchers must therefore continuously refine and adjust parameters based on specific practical scenarios. For instance, insufficient contact or inadequate applied pressure can lead to an actual contact area significantly smaller than the apparent area, substantially degrading the output performance. A larger contact-separation distance in TENGs generally leads to higher electrical output. However, the separation process may also introduce more complex dynamics and accelerated wear. Differences in the thermal expansion coefficients of various materials can cause variations in the gap distance of CS-TENG, thereby influencing their performance. Under environmental factors, the operational capability of TENGs under high-temperature conditions falls short of the demands imposed by rapidly advancing technologies. Due to thermionic emission effects, electrons transferred to the surface during contact electrification are released into the vacuum, resulting in the inability of TENGs to maintain effective electrical output at elevated temperatures. High-temperature working environments further exacerbate polymer wear and material transfer, causing loss of mechanical stability, which inevitably leads to further reduction in the effective output power of TENG. Moreover, given the sensitivity of TENGs to humidity, structural design for thermal energy harvesting systems should specifically incorporate protective measures to mitigate the impact of environmental moisture on TENG output performance. When transitioning from small-scale laboratory conditions to real-world applications, complexities such as dust and temperature variations also affect long-term stability. Mechanical excitations in practical environments are often random, low frequency, and low amplitude. It is significantly different from the ideal, high-frequency, and regular excitations typical in laboratory settings. Furthermore, ensuring identical contact area and pressure for each cycle is challenging, leading to unstable and unpredictable output, which is particularly disadvantageous for harvesting energy from irregular mechanical motions. To overcome the limitation of surface charge density, researchers can employ advanced principles such as charge pumping and charge shuttling to achieve reciprocating motion of charges within conductive domains. Employing non-contact or SE-TENG designs, TENGs based on air gaps or inductive electrification can operate without complete reliance on physical contact, thereby mitigating mechanical wear and reducing sensitivity to pressure. Furthermore, by incorporating micro-pillar arrays, porous sponge structures, or LM elements, these systems can achieve extensive surface contact even under low pressure through structural deformation. Through multilayer stacked designs and mechanically enhanced structures (such as springs, pendulums, and gear-driven mechanisms), the spatial utilization and energy conversion efficiency of TENGs can be significantly improved. It made them especially suitable for collecting low-frequency wave energy [299]. Developing robust TM-TENG, humidity, and thermal-resistive materials is necessary for improving durability and stable performance under extreme conditions. It is worth noting that TENG is not intended as a replacement for conventional high-power power supplies. But it rather should be regarded as a distributed energy solution tailored for the future “micro-energy world.” Translating wearable TM-TENGs from laboratory concepts into practical applications now faces core challenge that has shifted from mere performance optimization to meeting the systems engineering requirements of human compatibility, while maintaining an operating temperature within the human comfort range is the fundamental condition achievable by most TENGs. It represents only one dimension of safety. More critical, yet often overlooked, are the material’s inherent flexibility compatibility and its biosafety. Biosafety demands that TM-TENG materials should be systematically evaluated for long-term skin contact irritation, electromagnetic biological effects, and potential cytotoxicity, among other factors.
4. *Improving the stability of TENG devices* During the fabrication of composites, TENGs are prone to performance degradation due to wear of the triboelectric materials under prolonged operation. It relies on contact electrification for operation. When employed in thermal switching applications, a major challenge lies in achieving rapid, reversible, and low-energy-consumption transitions between thermal states. Current TENG-triggered thermal switches still require significant improvements in response time and cycling endurance. One promising approach involves the development of the emergent composites by embedding wear-resistant nanofillers into a polymer matrix, such as CNT or BN. Furthermore, self-healing materials are capable of autonomously repairing microcracks after damage, thereby significantly extending the service life of the devices. The application of superhydrophobic and corrosion-resistant surface coatings on TENGs can fundamentally isolate the core components from the effects of moisture and chemical corrosive agents. The development of efficient, robust, and flexible encapsulation strategies to completely isolate the core functional layers of TENGs from harsh external environments represents a critical engineering imperative for achieving long-term operational stability. Additionally, PCMs can be utilized for TM to mitigate the impact of temperature fluctuations on device performance. In terms of structural design, significant efforts should be devoted to developing non-contact or soft-contact configurations to physically avoid or mitigate direct friction and wear, such as SE-TENG and FT-TENG. Comprehensive stability research is an indispensable prerequisite for TENGs to penetrate broader application domains and high-value markets. In critical fields such as implantable medical devices, aerospace, and health TM monitoring, extremely high demands are placed on the long-term stability and absolute reliability of these devices. Furthermore, a paramount objective lies in the development of more efficient power management circuits. These circuits are essential for harvesting and storing the high-voltage, pulsed electrical energy generated by TENGs, subsequently converting it into stable, continuous, low-voltage direct current suitable for powering electronic devices. Integrating TENGs with other types of energy harvesters to form hybrid energy harvesting systems represents another critical direction, such as piezoelectric, electromagnetic, or TEGs. These hybrid systems leverage the complementary characteristics of different mechanisms to ensure a persistent power supply across diverse environmental conditions. For applications like intelligent TM, simple logic circuits or microcontrollers can be incorporated. These components dynamically adjust TM strategies in response to the output signals from TENGs, enabling adaptive and efficient system operation. In summary, in-depth investigation into TENG stability represents not only a compelling scientific endeavor but also an essential pathway toward transforming TENGs into mature and reliably practical technology. Currently, the cycling endurance of TENGs is generally maintained at a level exceeding 10,000 contact cycles, while high-performance materials can achieve cycle counts ranging from ten thousand to several hundred thousand. This progression is imperative for TENGs to evolve into a robust and commercially viable technology. Future breakthroughs in TM materials for TENGs will rely on interdisciplinary collaboration across materials science, mechanics, chemistry, electronic engineering, and artificial intelligence. Such synergistic innovation is essential to ultimately achieve long-term, stable, and reliable operation of TENGs under diverse complex environments, thereby truly ushering in a new era of broad industrial application.
5. *Exploring multifunctional applications* Materials for TM and TENGs are advancing rapidly toward intelligent, adaptive, and multifunctional integration. Combining them represents not merely an innovation in materials science, but a system-level revolution. Currently, TM-TENGs have been applied in fields such as intelligent wearables and electronic skin, energy and environmental monitoring, aerospace and extreme environments, as well as smart buildings and the IoT. Several cutting-edge intelligent applications are now under exploration. By integrating TENGs with TM materials within fibers, smart materials capable of simultaneous power generation and temperature regulation can be produced. Such adaptive thermal-regulating textiles can be realized utilizing the properties of PCM, thereby expanding the application scope of wearable TM-TENG materials. For specialized garments (such as those for soldiers or firefighters), the outer layer employs TENGs to harvest kinetic energy from movement. While the inner layer incorporates PCMs to absorb excess body thermal. The electricity generated can power built-in communication devices or sensors, achieving energy self-circulation and personal TM. Furthermore, self-managing energy systems for implantable medical devices increasingly rely on TM-TENGs. TENGs composed of biodegradable materials and biocompatible hydrogels, which equipped with TM capabilities. It can ensure that the devices do not cause tissue damage due to localized overheating during operation in vivo. The integration of TM-TENGs is driving the evolution of smart materials into multifunctional systems. Its core value lies in addressing the stability and functionality bottlenecks of TENGs in complex environments, while opening new application scenarios in areas such as wearable technology, the IoT, biomedical devices, and advanced manufacturing.
6. *Applying TM-TENG to practical electronic device applications* In the laboratory, TM-TENG devices must ensure functional reliability, which is demonstrated by stable and predictable performance within the target operating temperature range or across varying environmental conditions. In terms of structural design, excellent mechanical properties are required to enable the TM materials to effectively dissipate or buffer internal stresses generated by frictional contact, device deformation, and uneven thermal expansion. Crucially, composite materials must undergo durability testing to demonstrate how effective TM reduces the operating temperature of the device and slows the rate of material aging. Overall, current durability testing for TENG materials is generally required to achieve at least 10,000 cycles. Furthermore, in practical applications, the integration of modules and interfaces is critical for the efficient transfer of energy and signals. Efficiently connecting TM-TENGs with power management circuits, energy storage units, and microprocessors involves key considerations such as impedance matching, power flow management, and understanding how the TM strategy influences the overall thermal distribution of the module.

With the continuous evolution of the new era, researchers are integrating artificial intelligence (AI) and machine learning with TENG devices, providing effective solutions for processing the vast amounts of data generated by humans, machines, and the environment. The synergistic interaction between AI and TENG devices creates new opportunities for optimizing and enhancing data-intensive applications. By leveraging the unique characteristics of TM-TENGs, these systems can achieve broader implementation across multiple sectors, including healthcare, textiles, military, and manufacturing. The integration of TENGs with TM systems represents a significant paradigm shift from passive TM toward active, self-powered, and intelligent thermal regulation. Future research should aim to more precisely decouple the multiple effects introduced by TM strategies, such as stress modulation, alterations in dielectric properties, and wear behavior of friction layers. We call for the establishment of stricter experimental controls and theoretical models. It is essential to clarify the net benefits attributable to TM, rather than attributing improvements simplistically to the mere addition of a single material. We believe that, through synergistic innovation in new materials, novel structures, and advanced system design, TENGs are expected to overcome existing bottlenecks and emerge as reliable distributed energy sources for the IoT, wearable devices, and intelligent systems. Although TM-TENG technology is still in the early stages of transitioning from laboratory research to industrialization, it faced core challenges in energy efficiency, durability, and integration. The unique advantages of TM-TENG as a representative of the new generation of “micro-energy” technologies endow it with immense potential in fields such as wearable electronics, energy-efficient buildings, and advanced electronic cooling.
